# Supplementary material for: Off-season RSV epidemics in Australia after easing of COVID-19 restrictions
Source: Nat Commun. 2022 May 24;13:2884. doi: 10.1038/s41467-022-30485-3 (PMC9130497; doi:10.1038/s41467-022-30485-3)
Supplement: Supplementary file 1 — Supplementary Information [file 41467_2022_30485_MOESM1_ESM.pdf]

Off-season RSV epidemics in Australia after easing of COVID-19 restrictions

John-Sebastian Eden[1,2]*, Chisha Sikazwe[3,4]*, Ruopeng Xie[5,6]*, Yi-Mo Deng[7,8], Sheena G. Sullivan[7,9], Alice Michie[4], Avram Levy[3], Elena Cutmore[1,2], Christopher C Blyth[3,10-12], Philip N Britton[2,13], Nigel Crawford[14-16], Xiaomin Dong[7,8], Dominic E. Dwyer[2,17], Kimberly M. Edwards[5,6], Bethany A. Horsburgh[1,2], David Foley[3], Karina Kennedy[18], Cara Minney-Smith[3], David Speers[3,10], Rachel L. Tulloch[1,2], Edward C. Holmes[2], Vijaykrishna Dhanasekaran[5,6#], David W. Smith[3,10#], Jen Kok[17#] & Ian G. Barr[7,8#] and the Australian RSV study group[^]

1. Centre for Virus Research, Westmead Institute for Medical Research, Westmead, NSW 2145, Australia
2. Sydney Institute for Infectious Diseases, Sydney Medical School, The University of Sydney, Sydney, NSW 2006, Australia
3. PathWest Laboratory Medicine WA, Department of Microbiology, Nedlands, WA 6009, Australia
4. School of Biomedical Sciences, The University of Western Australia, Crawley, WA 6009, Australia
5. School of Public Health, LKS Faculty of Medicine, The University of Hong Kong, Hong Kong, China
6. HKU-Pasteur Research Pole, School of Public Health, LKS Faculty of Medicine, The University of Hong Kong, Hong Kong, China
7. WHO Collaborating Centre for Reference and Research on Influenza, Royal Melbourne Hospital, at the Peter Doherty Institute for Infection and Immunity, Melbourne, Victoria 3000, Australia
8. Department of Microbiology and Immunology, University of Melbourne, at the Peter Doherty Institute for Infection and Immunity, Melbourne, Victoria 3000, Australia
9. Department of Infectious Diseases, University of Melbourne, at the Peter Doherty Institute for Infection and Immunity, Melbourne, Victoria 3000, Australia
10. School of Medicine, The University of Western Australia, Crawley, WA 6009, Australia
11. Department of Infectious Diseases, Perth Children's Hospital, Nedlands, WA 6009, Australia
12. Wesfarmers Centre of Vaccines and Infectious Diseases, Telethon Kids Institute, Nedlands, WA 6009, Australia
13. Departments of Infectious Diseases and Microbiology, The Children's Hospital at Westmead, Westmead, NSW 2145, Australia
14. Murdoch Children's Research Institute, Melbourne, Victoria 3000, Australia
15. Department of Paediatrics, University of Melbourne & Royal Children's Hospital, Melbourne, Victoria 3000, Australia
16. Immunisation Service, Royal Children's Hospital, Melbourne, Victoria 3000, Australia
17. NSW Health Pathology - Institute for Clinical Pathology and Medical Research, Westmead Hospital, Westmead, NSW 2145, Australia
18. Departments of Clinical Microbiology and Infectious Diseases, Canberra Hospital, Garran, ACT 2605, Australia

*These authors contributed equally

[#]These authors jointly supervised this work

[^]A list of authors and their affiliations appears at the end of the main manuscript

Supplementary information

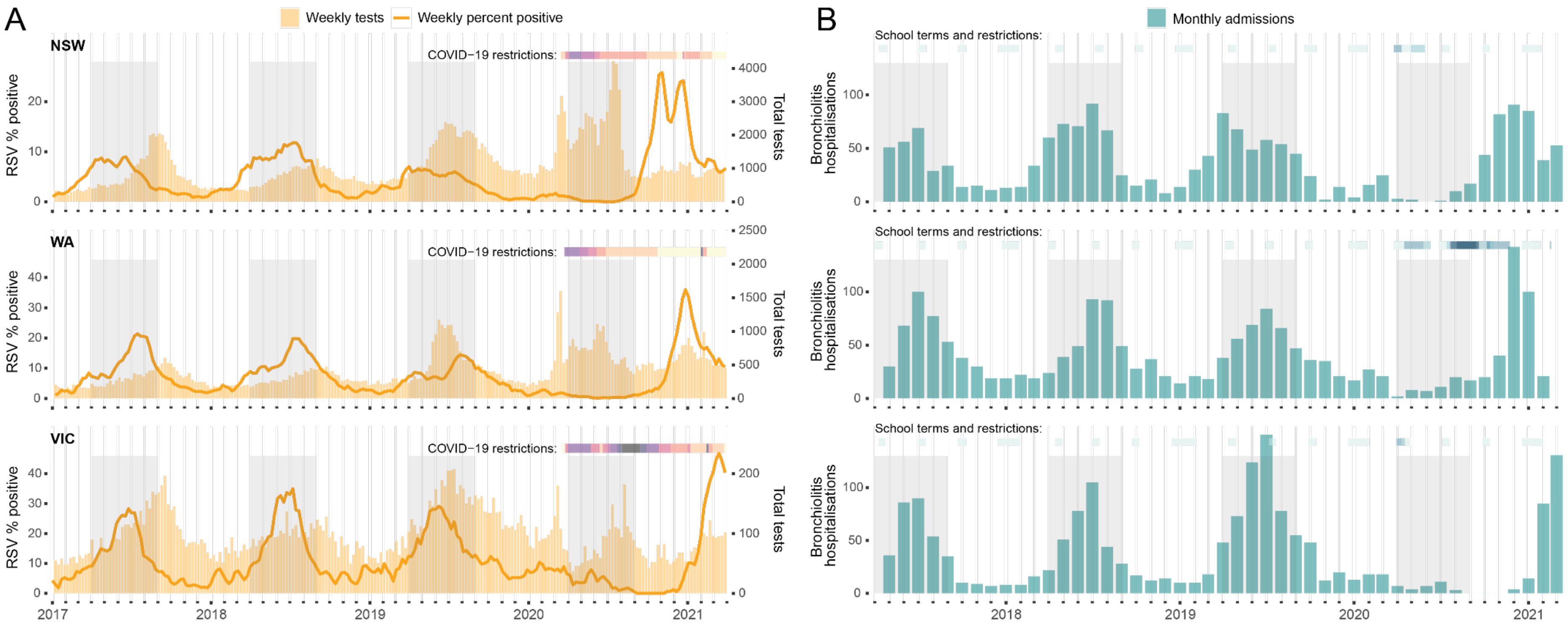

Supplementary Figure 1. Time series showing laboratory testing data (A) and RSV bronchiolitis admissions (B) for three Australian states – New South Wales (NSW), Western Australia (WA) and Victoria (VIC). Bars at the top of each state indicate COVID-19 restrictions or school holidays/restrictions for each state. For pandemic restrictions, darker colours indicate more severe restrictions. For schools, shading indicates school holiday periods, which darker shading indicates the period during which schools were closed or parents were encouraged to keep their children home. Grey shaded regions indicate the period 1st April to 1st September, which coincides with usual activity of RSV and other respiratory viruses.

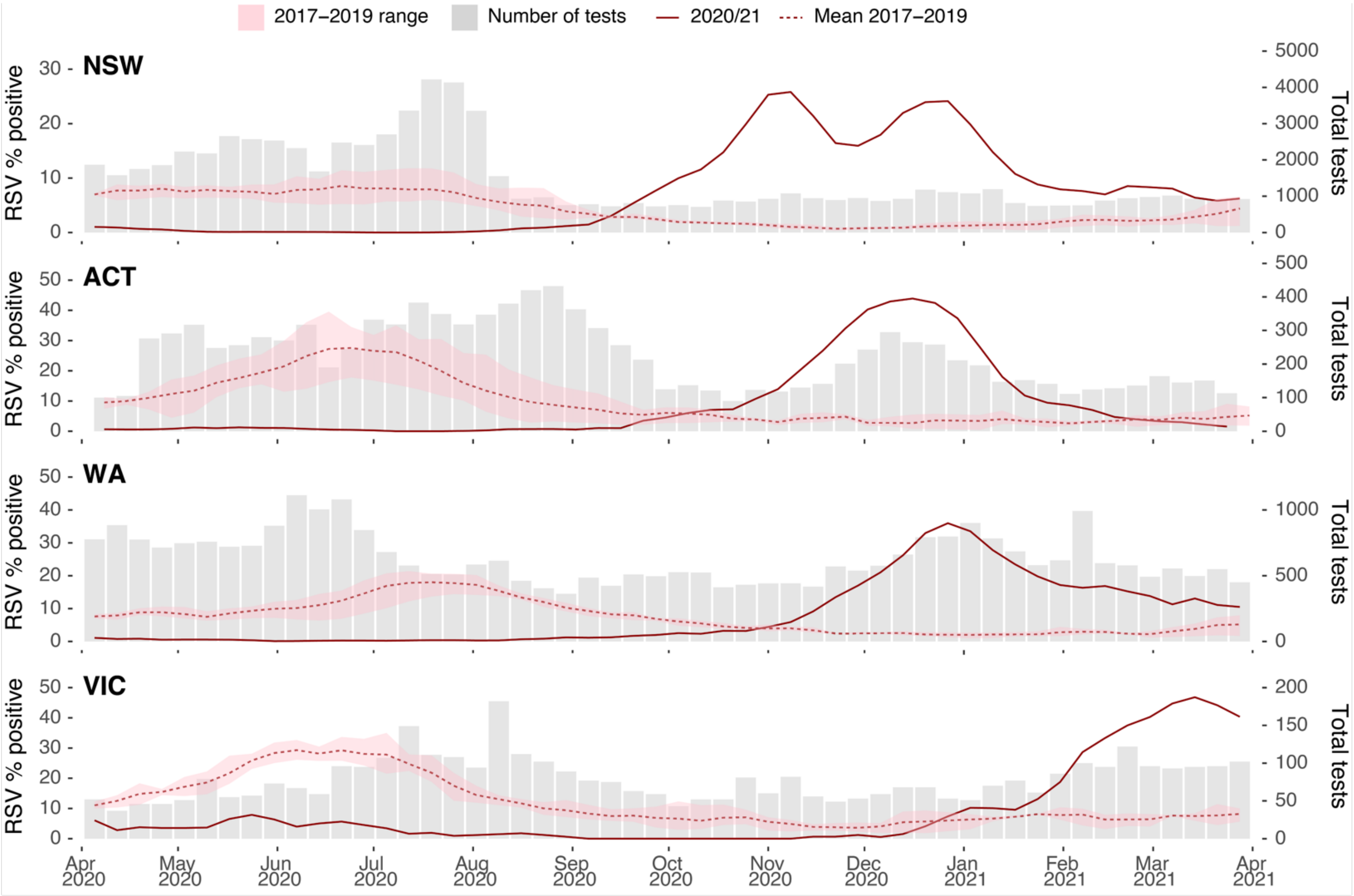

Supplementary Figure 2. Laboratory RSV detections in Australian states and territories – New South Wales (NSW), Western Australia (WA), Victoria (VIC) and Australian Capital Territory (ACT). Laboratory testing for RSV in 2020 as weekly percent positive (red line, left y-axis) and as total number of tests performed (grey bars, right y-axis). In each panel, the dashed red line represents mean monthly RSV percent positive over the three previous seasons, and corresponding red shading represents the range (minimum and maximum weekly percent positive).

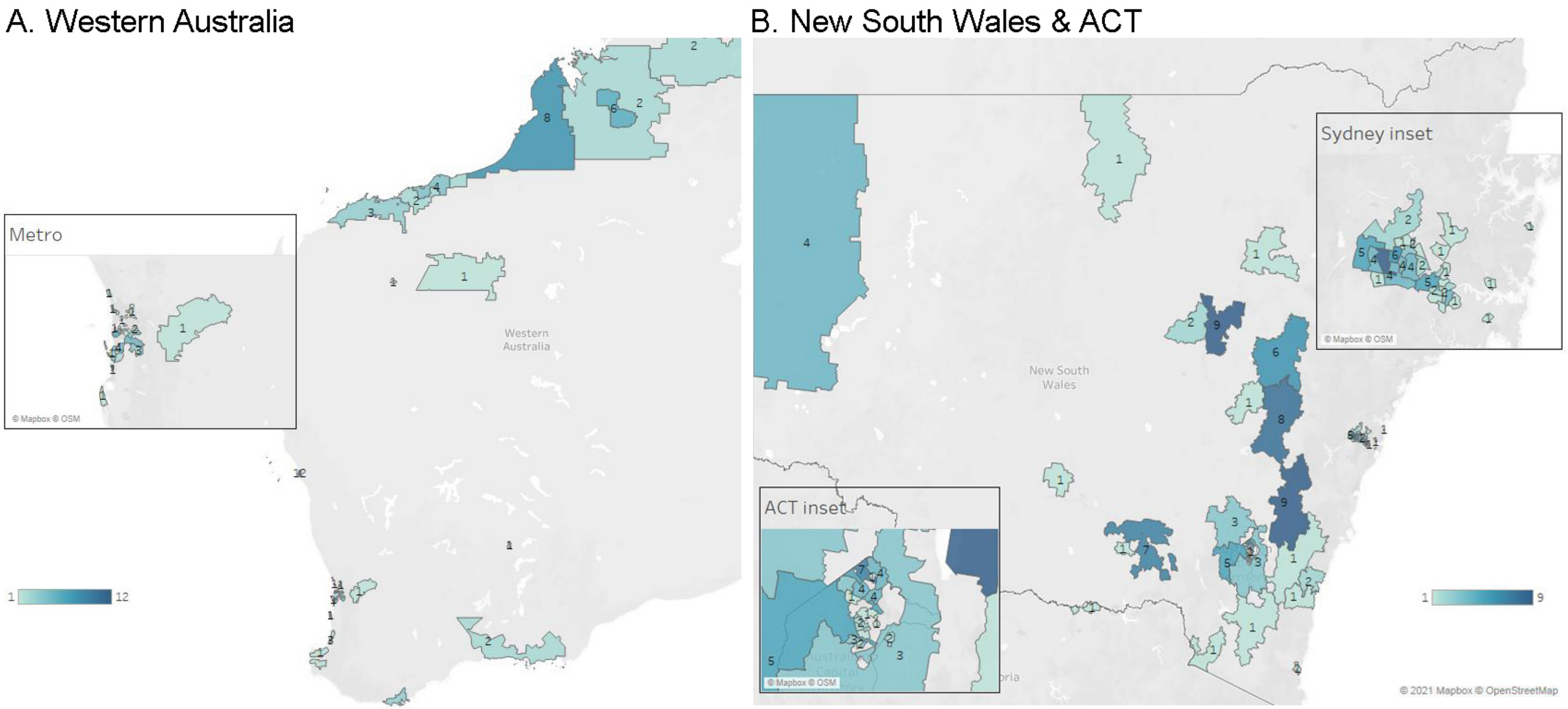

Supplementary Figure 3. Geographic distribution of RSV genome sequences in Western Australia (WA) and New South Wales (NSW) and Australian Capital Territory (ACT), Australia. The number of RSV genomes and their sampling location have been mapped according to postcode and coloured according to the keys provided for each state and territory. Population dense metropolitan areas, including Perth in WA, Sydney in NSW, and Canberra in the ACT are displayed as focused insets. OpenStreetMap® is open data and licensed under the Open Data Commons Open Database License (ODbL).

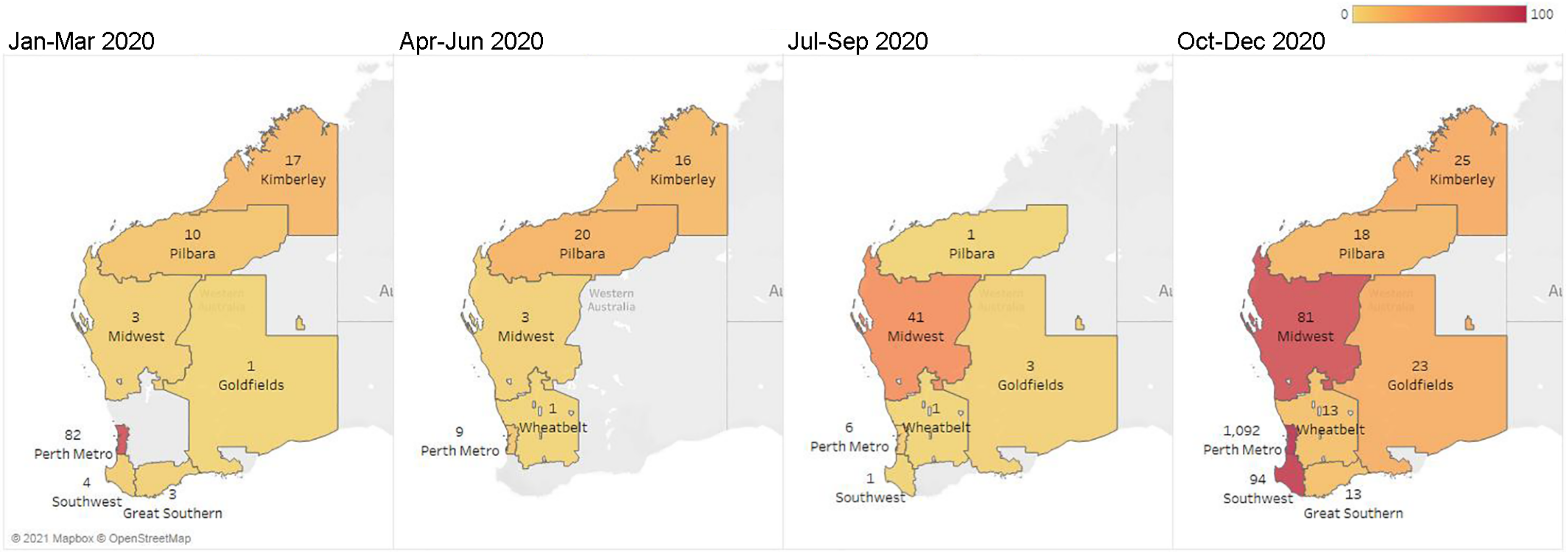

Supplementary Figure 4. Quarterly stratified sampling of total number of PCR positive RSV cases that underwent testing at PathWest Laboratory Medicine, Western Australia during 2020. Sampling locations were defined by region and coloured according to the total number of RSV cases within each region per quarter, with the darker colours being representative of increased case numbers. OpenStreetMap® is open data and licensed under the Open Data Commons Open Database License (ODbL).

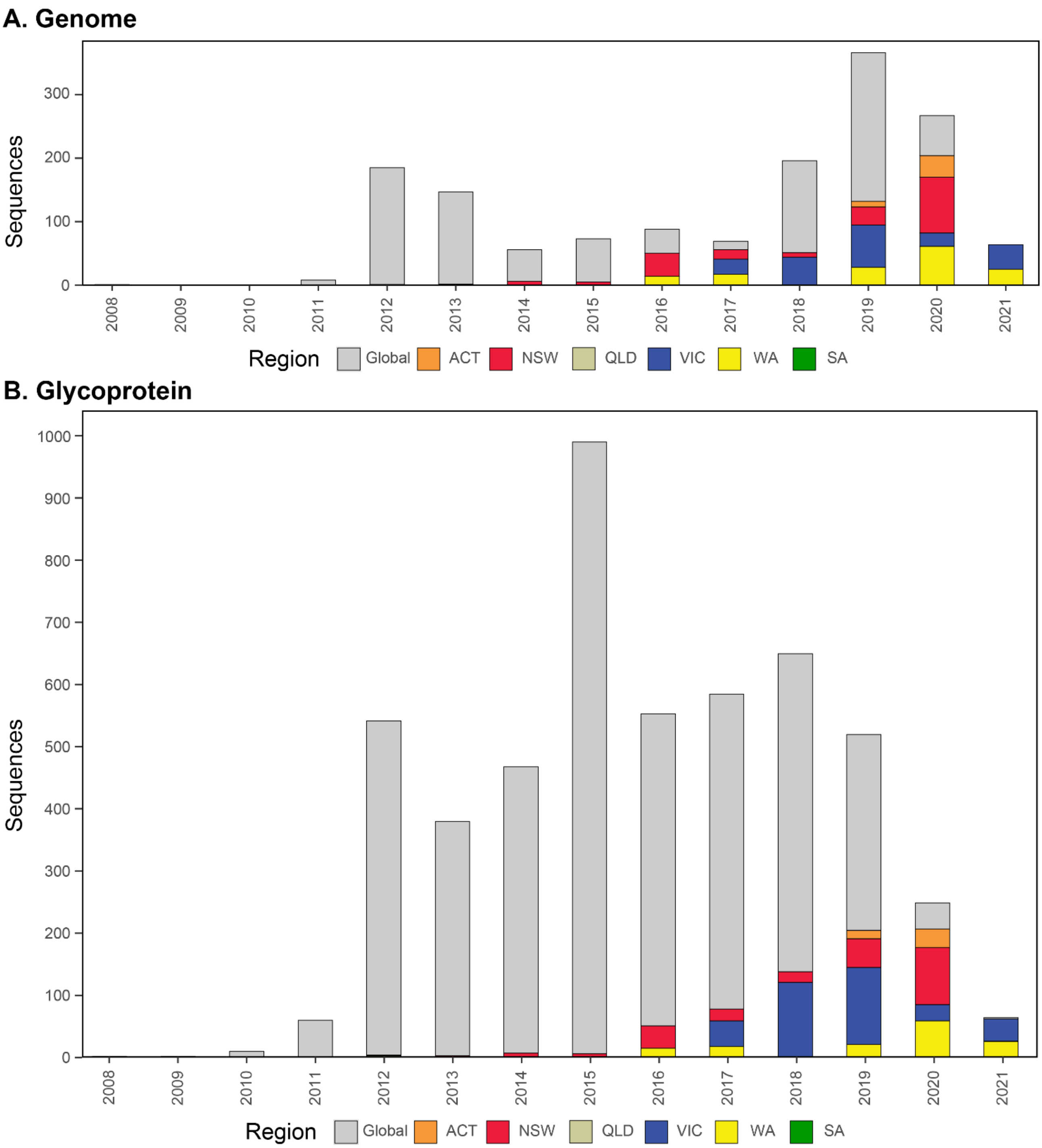

Supplementary Figure 5. Geographic and temporal sampling distributions for genomic analysis. These plots show the number of sequences (y-axis) by collection year (x-axis) for the genome (panel A) and glycoprotein (panel B) analysis presented in Figures 2 & 3, respectively. Australian states - New South Wales (NSW), Australia Capital Territory (ACT), Western Australia (WA) and Victoria (VIC), South Australia (SA) and Queensland (QLD) - and globally-derived sequences are coloured according to the keys provided.

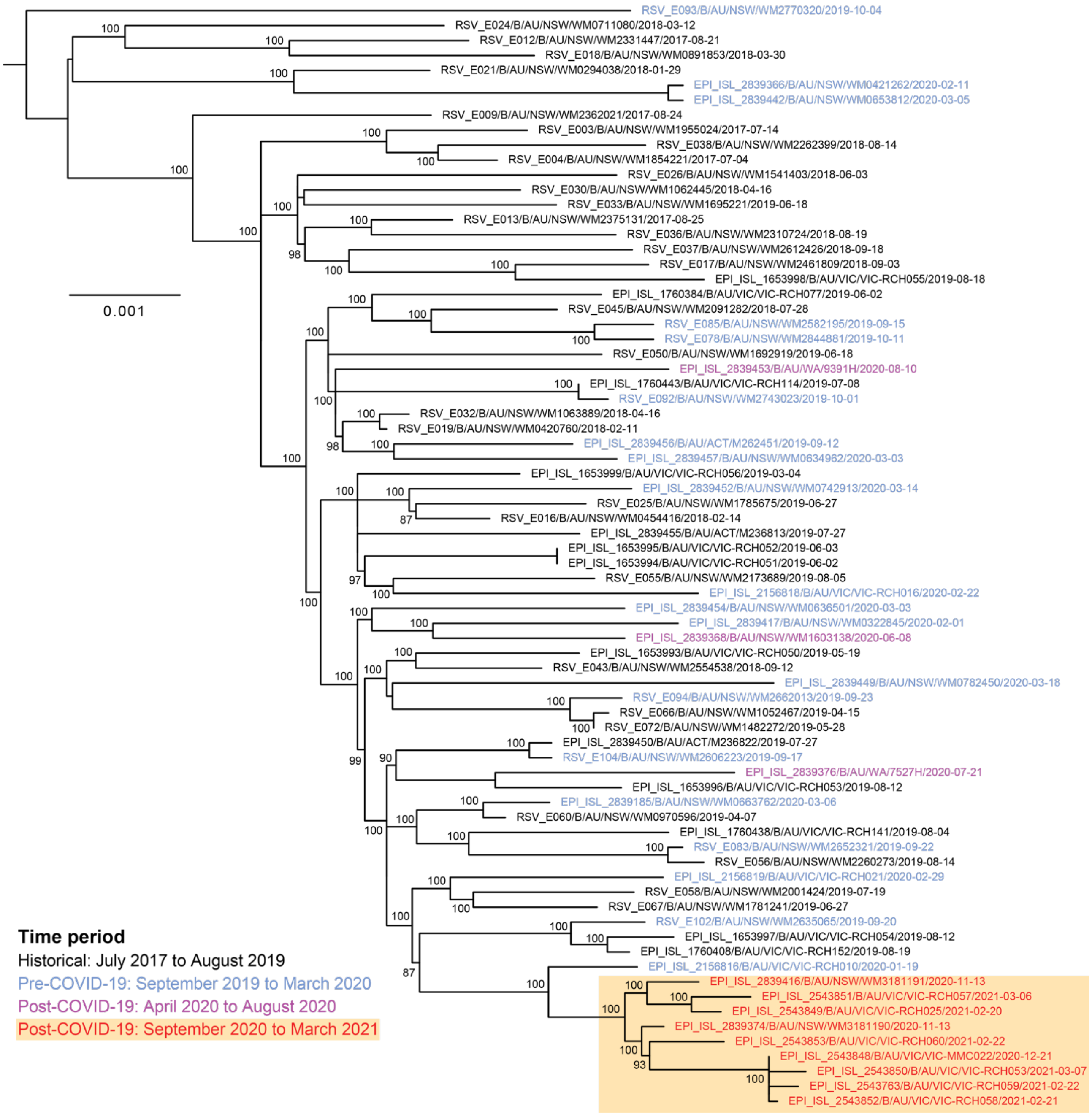

Supplementary Figure 6. Phylogenetic analysis of RSV-B diversity in Australia since mid-2017. RSV-B genome sequences generated in this study (n=74) were aligned and analysed phylogenetically using PhyML. The sequence labels show the accession, subtype, location, strain and collection date, and have been coloured according to the period they were collected as per the key provided. Values at nodes indicate bootstrap support values >70%. Branches are proportional to the number of nucleotide substitutions per site. The clade highlighted at the bottom represents a monophyletic lineage that emerged during the outbreak period of late 2020 to early 2021.

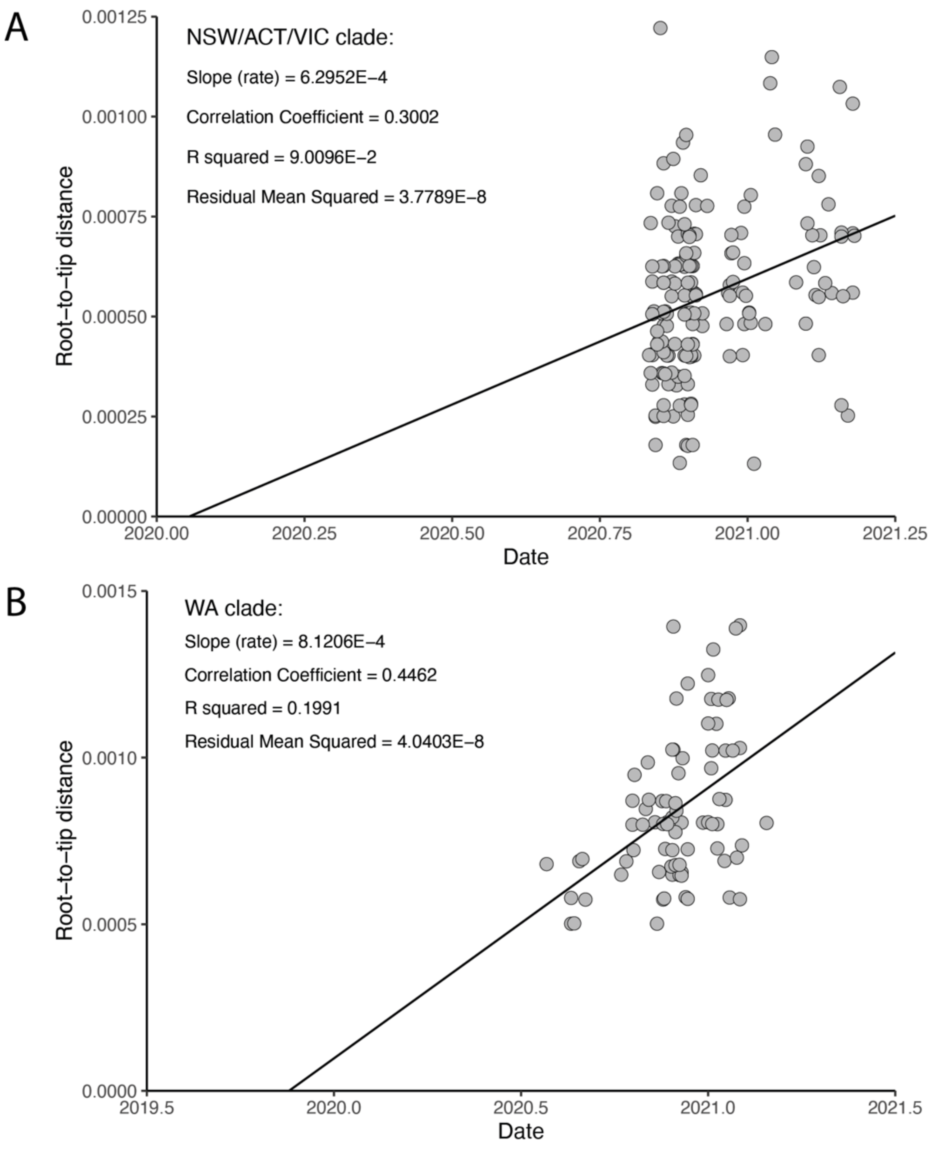

Supplementary Figure 7. Temporal signal in NSW/ACT/VIC and WA clades. Maximum likelihood phylogenies were prepared for each clade and root-to-tip distance (y-axis) was compared against sample collection date (x-axis) by linear-regression with each point representing one sequence in the phylogeny. NSW = New South Wales, ACT = Australian Capital Territory, VIC = Victoria and WA = Western Australia.

86 **Supplementary Table 1. Accession numbers for sequences generated in this study**

GenBank	GISAID	Virus	Location*	Collection
OM857140	EPI_ISL_2835616	hRSV/A/Australia/1514/2021	WA	2021-01-18
OM857141	EPI_ISL_2839389	hRSV/A/Australia/4005237/2020	WA	2020-10-13
OM857142	EPI_ISL_2839387	hRSV/A/Australia/1003657/2020	WA	2020-11-01
OM857143	EPI_ISL_2839381	hRSV/A/Australia/2002105/2020	WA	2020-11-22
OM857144	EPI_ISL_2839390	hRSV/A/Australia/6009368/2020	WA	2020-11-26
OM857145	EPI_ISL_2839391	hRSV/A/Australia/7007045/2020	WA	2020-11-27
OM857146	EPI_ISL_2839383	hRSV/A/Australia/3002009/2020	WA	2020-12-12
OM857147	EPI_ISL_2839388	hRSV/A/Australia/3002022/2020	WA	2020-12-12
OM857148	EPI_ISL_2839392	hRSV/A/Australia/9002215/2020	WA	2020-11-19
OM857149	EPI_ISL_2839378	hRSV/A/Australia/5005469/2021	WA	2021-01-05
OM857150	EPI_ISL_2839398	hRSV/A/Australia/3073/2020	WA	2020-11-20
OM857151	EPI_ISL_2839405	hRSV/A/Australia/4009301/2020	WA	2020-12-04
OM857152	EPI_ISL_2839406	hRSV/A/Australia/6003147/2020	WA	2020-12-06
OM857153	EPI_ISL_2839410	hRSV/A/Australia/8010547/2020	WA	2020-11-18
OM857154	EPI_ISL_2839443	hRSV/A/Australia/3904/2020	WA	2020-12-10
OM857155	EPI_ISL_2839446	hRSV/A/Australia/4004352/2020	WA	2020-11-14
OM857156	EPI_ISL_2839447	hRSV/A/Australia/6002331/2020	WA	2020-12-06
OM857157	EPI_ISL_2839441	hRSV/A/Australia/6003904/2020	WA	2020-12-06
OM857158	EPI_ISL_2839412	hRSV/A/Australia/7006882/2020	WA	2020-11-27
OM857159	EPI_ISL_2839414	hRSV/A/Australia/8001840/2020	WA	2020-10-08
OM857160	EPI_ISL_2839440	hRSV/A/Australia/4011388/2020	WA	2020-11-04
OM857161	EPI_ISL_2839430	hRSV/A/Australia/3001519/2020	WA	2020-11-03
OM857162	EPI_ISL_2839448	hRSV/A/Australia/9005740/2020	WA	2020-10-29
OM857163	EPI_ISL_2839432	hRSV/A/Australia/5584/2020	WA	2020-11-30
OM857164	EPI_ISL_2839418	hRSV/A/Australia/1003117/2020	WA	2020-11-10
OM857165	EPI_ISL_2839179	hRSV/A/Australia/1006644/2020	WA	2020-11-21
OM857166	EPI_ISL_2839431	hRSV/A/Australia/2002690/2020	WA	2020-11-22
OM857167	EPI_ISL_2839399	hRSV/A/Australia/3471/2020	WA	2020-10-20
OM857168	EPI_ISL_2839409	hRSV/A/Australia/7003912/2020	WA	2020-11-17
OM857169	EPI_ISL_2839415	hRSV/A/Australia/8002008/2020	WA	2020-11-27
OM857170	EPI_ISL_2839407	hRSV/A/Australia/9002436/2020	WA	2020-11-28
OM857171	EPI_ISL_2839439	hRSV/A/Australia/3012220/2020	WA	2020-12-03
OM857172	EPI_ISL_2839364	hRSV/A/Australia/9005762/2020	WA	2020-10-19
OM857173	EPI_ISL_2839172	hRSV/A/Australia/9005783/2020	WA	2020-10-19
OM857174	EPI_ISL_2839438	hRSV/A/Australia/1011044/2020	WA	2020-12-01
OM857175	EPI_ISL_2839444	hRSV/A/Australia/1002541/2021	WA	2021-01-01
OM857176	EPI_ISL_2543773	hRSV/A/Australia/MMC045/2020	VIC	2020-12-27
OM857177	EPI_ISL_2543777	hRSV/A/Australia/MMC055/2021	VIC	2021-01-10
OM857178	EPI_ISL_2839386	hRSV/A/Australia/2051/2021	WA	2021-01-29
OM857179	EPI_ISL_2839183	hRSV/A/Australia/6492G/2020	WA	2020-08-20
OM857180	EPI_ISL_2839413	hRSV/A/Australia/7494P/2020	WA	2020-09-03
OM857181	EPI_ISL_2839433	hRSV/A/Australia/8003496/2021	WA	2021-01-17

OM857182	EPI_ISL_2839385	hRSV/A/Australia/7680J/2020	WA	2020-08-23
OM857183	EPI_ISL_2839195	hRSV/A/Australia/8005369/2021	WA	2021-01-18
OM857184	EPI_ISL_2839429	hRSV/A/Australia/5003133/2021	WA	2021-01-04
OM857185	EPI_ISL_2839423	hRSV/A/Australia/3004170/2021	WA	2021-01-21
OM857186	EPI_ISL_2839199	hRSV/A/Australia/1643/2021	WA	2021-01-10
OM857187	EPI_ISL_2839170	hRSV/A/Australia/6545C/2020	WA	2020-08-20
OM857188	EPI_ISL_2839445	hRSV/A/Australia/3002027/2021	WA	2021-01-22
OM857189	EPI_ISL_2839411	hRSV/A/Australia/1008347/2021	WA	2021-02-01
OM857190	EPI_ISL_2839171	hRSV/A/Australia/3010918/2021	WA	2021-02-03
OM857191	EPI_ISL_2839402	hRSV/A/Australia/3004/2021	WA	2021-01-09
OM857192	EPI_ISL_2839408	hRSV/A/Australia/2001479/2020	WA	2020-10-21
OM857193	EPI_ISL_2839400	hRSV/A/Australia/3002650/2021	WA	2021-01-01
OM857194	EPI_ISL_2839404	hRSV/A/Australia/3002687/2021	WA	2021-01-01
OM857195	EPI_ISL_2839403	hRSV/A/Australia/1010209/2021	WA	2021-01-11
OM857196	EPI_ISL_2839401	hRSV/A/Australia/5009934/2021	WA	2021-01-04
OM857197	EPI_ISL_2839397	hRSV/A/Australia/9009560/2021	WA	2021-01-19
OM857198	EPI_ISL_2839379	hRSV/A/Australia/2003588/2020	WA	2020-12-12
OM857199	EPI_ISL_2839384	hRSV/A/Australia/8001314/2020	WA	2020-11-27
OM857200	EPI_ISL_2839372	hRSV/A/Australia/8006635/2020	WA	2020-11-28
OM857201	EPI_ISL_2839176	hRSV/A/Australia/6002591/2021	WA	2021-01-25
OM857202	EPI_ISL_2839181	hRSV/A/Australia/5516M/2020	WA	2020-07-27
OM857203	EPI_ISL_2839182	hRSV/A/Australia/7199H/2020	WA	2020-08-31
OM857204	EPI_ISL_2839184	hRSV/A/Australia/1007437/2021	WA	2021-02-01
OM857205	EPI_ISL_2839177	hRSV/A/Australia/6002451/2021	WA	2021-01-05
OM857206	EPI_ISL_2839174	hRSV/A/Australia/3160/2020	WA	2020-11-30
OM857207	EPI_ISL_2839194	hRSV/A/Australia/4010993/2020	WA	2020-12-04
OM857208	EPI_ISL_2839192	hRSV/A/Australia/7006894/2020	WA	2020-12-07
OM857209	EPI_ISL_2839175	hRSV/A/Australia/6992X/2020	WA	2020-08-28
OM857210	EPI_ISL_2839370	hRSV/A/Australia/1004203/2020	WA	2020-11-30
OM857211	EPI_ISL_2839421	hRSV/A/Australia/8006798/2021	WA	2021-01-28
OM857212	EPI_ISL_2839173	hRSV/A/Australia/6012043/2021	WA	2021-01-06
OM857213	EPI_ISL_2839300	hRSV/A/Australia/N122610/2020	ACT	2020-11-23
OM857214	EPI_ISL_2839351	hRSV/A/Australia/P510179/2020	ACT	2020-11-19
OM857215	EPI_ISL_2839213	hRSV/A/Australia/P511402/2020	ACT	2020-11-27
OM857216	EPI_ISL_2839275	HRSV/A/Australia/WM3081573/2020	NSW	2020-11-03
OM857217	EPI_ISL_2839290	HRSV/A/Australia/WM3200400/2020	NSW	2020-11-15
OM857218	EPI_ISL_2839264	HRSV/A/Australia/WM3090116/2020	NSW	2020-11-04
OM857219	EPI_ISL_2839256	HRSV/A/Australia/WM3160671/2020	NSW	2020-11-11
OM857220	EPI_ISL_2839277	HRSV/A/Australia/WM3120229/2020	NSW	2020-11-07
OM857221	EPI_ISL_2839279	HRSV/A/Australia/WM3140467/2020	NSW	2020-11-09
OM857222	EPI_ISL_2839288	HRSV/A/Australia/WM3150812/2020	NSW	2020-11-10
OM857223	EPI_ISL_2839266	HRSV/A/Australia/WM3202125/2020	NSW	2020-11-15
OM857224	EPI_ISL_2839208	HRSV/A/Australia/WM3071668/2020	NSW	2020-11-02
OM857225	EPI_ISL_2839235	HRSV/A/Australia/WM3181215/2020	NSW	2020-11-13

OM857226	EPI_ISL_2839237	HRSV/A/Australia/WM3260993/2020	NSW	2020-11-21
OM857227	EPI_ISL_2839354	hRSV/A/Australia/P514660/2020	ACT	2020-11-20
OM857228	EPI_ISL_2839310	hRSV/A/Australia/P520872/2020	ACT	2020-11-27
OM857229	EPI_ISL_2839215	hRSV/A/Australia/P527755/2020	ACT	2020-11-28
OM857230	EPI_ISL_2839332	HRSV/A/Australia/WM3111229/2020	NSW	2020-11-06
OM857231	EPI_ISL_2839285	HRSV/A/Australia/WM3300592/2020	NSW	2020-11-25
OM857232	EPI_ISL_2839221	HRSV/A/Australia/WM3142234/2020	NSW	2020-11-09
OM857233	EPI_ISL_2839334	HRSV/A/Australia/WM3152749/2020	NSW	2020-11-10
OM857234	EPI_ISL_2839328	HRSV/A/Australia/WM3160376/2020	NSW	2020-11-11
OM857235	EPI_ISL_2839248	HRSV/A/Australia/WM3171598/2020	NSW	2020-11-12
OM857236	EPI_ISL_2839250	HRSV/A/Australia/WM3220266/2020	NSW	2020-11-17
OM857237	EPI_ISL_2839252	HRSV/A/Australia/WM3220890/2020	NSW	2020-11-17
OM857238	EPI_ISL_2839226	HRSV/A/Australia/WM3253207/2020	NSW	2020-11-20
OM857239	EPI_ISL_2839296	hRSV/A/Australia/P511810/2020	ACT	2020-11-27
OM857240	EPI_ISL_2839227	hRSV/A/Australia/P527750/2020	ACT	2020-11-28
OM857241	EPI_ISL_2839207	HRSV/A/Australia/WM3145009/2020	NSW	2020-11-09
OM857242	EPI_ISL_2839339	HRSV/A/Australia/WM3281572/2020	NSW	2020-11-23
OM857243	EPI_ISL_2839242	HRSV/A/Australia/WM3301231/2020	NSW	2020-11-25
OM857244	EPI_ISL_2839188	HRSV/A/Australia/WM3073568/2020	NSW	2020-11-02
OM857245	EPI_ISL_2839206	HRSV/A/Australia/WM3150792/2020	NSW	2020-11-10
OM857246	EPI_ISL_2839204	HRSV/A/Australia/WM3151215/2020	NSW	2020-11-10
OM857247	EPI_ISL_2839205	HRSV/A/Australia/WM3301586/2020	NSW	2020-11-25
OM857248	EPI_ISL_2839202	HRSV/A/Australia/WM3281795/2020	NSW	2020-11-23
OM857249	EPI_ISL_2839294	hRSV/A/Australia/P514373/2020	ACT	2020-11-23
OM857250	EPI_ISL_2839198	HRSV/A/Australia/WM3303286/2020	NSW	2020-11-25
OM857251	EPI_ISL_2839356	hRSV/A/Australia/P511794/2020	ACT	2020-11-27
OM857252	EPI_ISL_2839312	hRSV/A/Australia/P536277/2020	ACT	2020-12-04
OM857253	EPI_ISL_2839324	HRSV/A/Australia/WM3341974/2020	NSW	2020-11-29
OM857254	EPI_ISL_2839362	HRSV/A/Australia/WM3152673/2020	NSW	2020-11-10
OM857255	EPI_ISL_2543764	hRSV/A/Australia/VIC-MMC007/2021	VIC	2021-01-02
OM857256	EPI_ISL_2543771	hRSV/A/Australia/VIC-MMC038/2020	VIC	2020-12-23
OM857257	EPI_ISL_2543784	hRSV/A/Australia/VIC-RCH008/2021	VIC	2021-01-31
OM857258	EPI_ISL_2543772	hRSV/A/Australia/VIC-MMC039/2020	VIC	2020-12-23
OM857259	EPI_ISL_2839326	HRSV/A/Australia/WM3114268/2020	NSW	2020-11-06
OM857260	EPI_ISL_2839360	HRSV/A/Australia/WM3341608/2020	NSW	2020-11-29
OM857261	EPI_ISL_2839322	HRSV/A/Australia/WM3293488/2020	NSW	2020-11-24
OM857262	EPI_ISL_2543770	hRSV/A/Australia/VIC-MMC035/2020	VIC	2020-12-22
OM857263	EPI_ISL_2543786	hRSV/A/Australia/VIC-RCH010/2021	VIC	2021-02-06
OM857264	EPI_ISL_2543800	hRSV/A/Australia/VIC-RCH035/2021	VIC	2021-02-28
OM857265	EPI_ISL_2839189	HRSV/A/Australia/WM3080072/2020	NSW	2020-11-03
OM857266	EPI_ISL_2839419	HRSV/A/Australia/WM3151529/2020	NSW	2020-11-10
OM857267	EPI_ISL_1653939	hRSV/A/Australia/VIC-RCH001/2021	VIC	2021-01-02
OM857268	EPI_ISL_2839425	hRSV/A/Australia/P506175/2020	ACT	2020-11-17
OM857269	EPI_ISL_2839427	hRSV/A/Australia/P527732/2020	ACT	2020-11-28

OM857270	EPI_ISL_2839302	hRSV/A/Australia/P507350/2020	ACT	2020-11-20
OM857271	EPI_ISL_2839231	hRSV/A/Australia/P508521/2020	ACT	2020-11-19
OM857272	EPI_ISL_2839228	hRSV/A/Australia/P511846/2020	ACT	2020-11-20
OM857273	EPI_ISL_2839304	hRSV/A/Australia/P514323/2020	ACT	2020-11-23
OM857274	EPI_ISL_2839232	hRSV/A/Australia/P525519/2020	ACT	2020-11-30
OM857275	EPI_ISL_2839298	hRSV/A/Australia/P526371/2020	ACT	2020-11-30
OM857276	EPI_ISL_2839273	HRSV/A/Australia/WM3351169/2020	NSW	2020-11-30
OM857277	EPI_ISL_2839217	hRSV/A/Australia/P522722/2020	ACT	2020-11-29
OM857278	EPI_ISL_2839214	hRSV/A/Australia/P527905/2020	ACT	2020-11-25
OM857279	EPI_ISL_2839229	hRSV/A/Australia/P510167/2020	ACT	2020-11-23
OM857280	EPI_ISL_2839292	HRSV/A/Australia/WM3280848/2020	NSW	2020-11-23
OM857281	EPI_ISL_2839347	HRSV/A/Australia/WM3312123/2020	NSW	2020-11-26
OM857282	EPI_ISL_2839216	hRSV/A/Australia/P525045/2020	ACT	2020-11-30
OM857283	EPI_ISL_2839233	hRSV/A/Australia/P515852/2020	ACT	2020-11-28
OM857284	EPI_ISL_2839230	hRSV/A/Australia/P520806/2020	ACT	2020-11-28
OM857285	EPI_ISL_2839306	hRSV/A/Australia/P517150/2020	ACT	2020-11-20
OM857286	EPI_ISL_2839308	hRSV/A/Australia/P507573/2020	ACT	2020-11-19
OM857287	EPI_ISL_2839424	hRSV/A/Australia/P520545/2020	ACT	2020-11-29
OM857288	EPI_ISL_2839218	hRSV/A/Australia/P533369/2020	ACT	2020-12-03
OM857289	EPI_ISL_2839234	hRSV/A/Australia/P538685/2020	ACT	2020-12-07
OM857290	EPI_ISL_1653938	hRSV/A/Australia/VIC-VIDRL001/2020	VIC	2020-12-22
OM857291	EPI_ISL_2839190	hRSV/A/Australia/P520445/2020	ACT	2020-11-26
OM857292	EPI_ISL_2839281	HRSV/A/Australia/WM3171321/2020	NSW	2020-11-12
OM857293	EPI_ISL_2839258	HRSV/A/Australia/WM3221617/2020	NSW	2020-11-17
OM857294	EPI_ISL_2839197	hRSV/A/Australia/P535659/2020	ACT	2020-12-04
OM857295	EPI_ISL_2839196	HRSV/A/Australia/WM3151688/2020	NSW	2020-11-10
OM857296	EPI_ISL_2839260	HRSV/A/Australia/WM3060152/2020	NSW	2020-11-01
OM857297	EPI_ISL_2839254	HRSV/A/Australia/WM3081844/2020	NSW	2020-11-03
OM857298	EPI_ISL_2839244	HRSV/A/Australia/WM3302850/2020	NSW	2020-11-25
OM857299	EPI_ISL_2839320	HRSV/A/Australia/WM3084781/2020	NSW	2020-11-03
OM857300	EPI_ISL_2839343	HRSV/A/Australia/WM3221870/2020	NSW	2020-11-17
OM857301	EPI_ISL_2839318	HRSV/A/Australia/WM3100311/2020	NSW	2020-11-05
OM857302	EPI_ISL_2839341	HRSV/A/Australia/WM3101273/2020	NSW	2020-11-05
OM857303	EPI_ISL_2839236	HRSV/A/Australia/WM3212551/2020	NSW	2020-11-16
OM857304	EPI_ISL_2839345	HRSV/A/Australia/WM3292889/2020	NSW	2020-11-24
OM857305	EPI_ISL_2839316	HRSV/A/Australia/WM3301616/2020	NSW	2020-11-25
OM857306	EPI_ISL_2839358	HRSV/A/Australia/WM3101170/2020	NSW	2020-11-05
OM857307	EPI_ISL_2839330	HRSV/A/Australia/WM3331626/2020	NSW	2020-11-28
OM857308	EPI_ISL_2839337	HRSV/A/Australia/WM3151664/2020	NSW	2020-11-10
OM857309	EPI_ISL_2839222	HRSV/A/Australia/WM3180278/2020	NSW	2020-11-13
OM857310	EPI_ISL_2839225	HRSV/A/Australia/WM3301224/2020	NSW	2020-11-25
OM857311	EPI_ISL_2839209	HRSV/A/Australia/WM3231345/2020	NSW	2020-11-18
OM857312	EPI_ISL_2839210	HRSV/A/Australia/WM3321046/2020	NSW	2020-11-27
OM857313	EPI_ISL_2839269	HRSV/A/Australia/WM3280473/2020	NSW	2020-11-23

OM857314	EPI_ISL_2839271	HRSV/A/Australia/WM3341337/2020	NSW	2020-11-29
OM857315	EPI_ISL_2839246	HRSV/A/Australia/WM3180542/2020	NSW	2020-11-13
OM857316	EPI_ISL_2839224	HRSV/A/Australia/WM3202528/2020	NSW	2020-11-15
OM857317	EPI_ISL_2839313	HRSV/A/Australia/WM3291066/2020	NSW	2020-11-24
OM857318	EPI_ISL_2839239	HRSV/A/Australia/WM3141851/2020	NSW	2020-11-09
OM857319	EPI_ISL_2839219	HRSV/A/Australia/WM3151206/2020	NSW	2020-11-10
OM857320	EPI_ISL_2839223	HRSV/A/Australia/WM3323284/2020	NSW	2020-11-27
OM857321	EPI_ISL_2839262	HRSV/A/Australia/WM3312926/2020	NSW	2020-11-26
OM857322	EPI_ISL_2839220	HRSV/A/Australia/WM3201552/2020	NSW	2020-11-15
OM857323	EPI_ISL_2839349	HRSV/A/Australia/WM3324484/2020	NSW	2020-11-27
OM857324	EPI_ISL_2839203	HRSV/A/Australia/WM3321824/2020	NSW	2020-11-27
OM857325	EPI_ISL_2839283	HRSV/A/Australia/WM3322984/2020	NSW	2020-11-27
OM857326	EPI_ISL_2839187	HRSV/A/Australia/WM3071478/2020	NSW	2020-11-02
OM857327	EPI_ISL_2839186	HRSV/A/Australia/WM3071531/2020	NSW	2020-11-02
OM857328	EPI_ISL_2839420	HRSV/A/Australia/WM3174399/2020	NSW	2020-11-12
OM857329	EPI_ISL_2839191	HRSV/A/Australia/WM3151642/2020	NSW	2020-11-10
OM857330	EPI_ISL_1653941	hRSV/A/Australia/VIC-RCH002/2020	VIC	2020-12-20
OM857331	EPI_ISL_1653944	hRSV/A/Australia/VIC-VIDRL003/2020	VIC	2020-12-28
OM857332	EPI_ISL_1653948	hRSV/A/Australia/VIC-VIDRL005/2020	VIC	2020-12-29
OM857333	EPI_ISL_1653946	hRSV/A/Australia/VIC-VIDRL004/2020	VIC	2020-12-29
OM857334	EPI_ISL_2811729	hRSV/A/Australia/VIC-RCH007/2020	VIC	2020-11-26
OM857335	EPI_ISL_2543765	hRSV/A/Australia/VIC-MMC014/2020	VIC	2020-12-19
OM857336	EPI_ISL_2543787	hRSV/A/Australia/VIC-RCH011/2021	VIC	2021-02-06
OM857337	EPI_ISL_2543797	hRSV/A/Australia/VIC-RCH030/2021	VIC	2021-02-22
OM857338	EPI_ISL_2543789	hRSV/A/Australia/VIC-RCH013/2021	VIC	2021-02-12
OM857339	EPI_ISL_2543774	hRSV/A/Australia/VIC-MMC053/2020	VIC	2020-12-30
OM857340	EPI_ISL_2543780	hRSV/A/Australia/VIC-MMC065/2021	VIC	2021-01-12
OM857341	EPI_ISL_2543805	hRSV/A/Australia/VIC-RCH048/2021	VIC	2021-03-07
OM857342	EPI_ISL_2543807	hRSV/A/Australia/VIC-RCH051/2021	VIC	2021-03-08
OM857343	EPI_ISL_2543767	hRSV/A/Australia/VIC-MMC027/2020	VIC	2020-12-21
OM857344	EPI_ISL_2543778	hRSV/A/Australia/VIC-MMC064/2020	VIC	2020-12-31
OM857345	EPI_ISL_2543795	hRSV/A/Australia/VIC-RCH022/2021	VIC	2021-02-15
OM857346	EPI_ISL_2543790	hRSV/A/Australia/VIC-RCH016/2021	VIC	2021-02-11
OM857347	EPI_ISL_2543804	hRSV/A/Australia/VIC-RCH047/2021	VIC	2021-03-04
OM857348	EPI_ISL_2839428	HRSV/A/Australia/WM3291678/2020	NSW	2020-11-24
OM857349	EPI_ISL_2839422	HRSV/A/Australia/WM3110533/2020	NSW	2020-11-06
OM857350	EPI_ISL_2543775	hRSV/A/Australia/VIC-MMC054/2020	VIC	2020-12-30
OM857351	EPI_ISL_2543762	hRSV/A/Australia/VIC-MMC073/2021	VIC	2021-01-13
OM857352	EPI_ISL_2543783	hRSV/A/Australia/VIC-RCH007/2021	VIC	2021-01-16
OM857353	EPI_ISL_2543796	hRSV/A/Australia/VIC-RCH026/2021	VIC	2021-02-20
OM857354	EPI_ISL_2543798	hRSV/A/Australia/VIC-RCH031/2021	VIC	2021-02-27
OM857355	EPI_ISL_2543769	hRSV/A/Australia/VIC-MMC033/2020	VIC	2020-12-21
OM857356	EPI_ISL_2543785	hRSV/A/Australia/VIC-RCH009/2021	VIC	2021-02-07
OM857357	EPI_ISL_2839212	HRSV/A/Australia/WM3080869/2020	NSW	2020-11-03

OM857358	EPI_ISL_2839211	HRSV/A/Australia/WM3291444/2020	NSW	2020-11-24
OM857359	EPI_ISL_2543766	hRSV/A/Australia/VIC-MMC024/2020	VIC	2020-12-21
OM857360	EPI_ISL_2543781	hRSV/A/Australia/VIC-MMC099/2021	VIC	2021-01-18
OM857361	EPI_ISL_2839451	hRSV/A/Australia/P522837/2020	ACT	2020-11-28
OM857362	EPI_ISL_2839201	HRSV/A/Australia/WM3131917/2020	NSW	2020-11-08
OM857363	EPI_ISL_1653942	hRSV/A/Australia/VIC-RCH002/2021	VIC	2021-01-03
OM857364	EPI_ISL_2839200	HRSV/A/Australia/WM3210899/2020	NSW	2020-11-16
OM857365	EPI_ISL_2839178	hRSV/A/Australia/P513256/2020	ACT	2020-11-22
OM857366	EPI_ISL_2839395	hRSV/A/Australia/1013898/2021	WA	2021-02-01
OM857367	EPI_ISL_2839396	hRSV/A/Australia/2010230/2020	WA	2020-11-12
OM857368	EPI_ISL_2839376	hRSV/B/Australia/7527H/2019	WA	1905-07-11
OM857369	EPI_ISL_2839455	hRSV/B/Australia/M236813/2019	ACT	2019-07-27
OM857370	EPI_ISL_2839452	HRSV/B/Australia/WM0742913/2020	NSW	2020-03-14
OM857371	EPI_ISL_2839456	hRSV/B/Australia/M262451/2019	ACT	2019-09-12
OM857372	EPI_ISL_2839457	HRSV/B/Australia/WM0634962/2020	NSW	2020-03-03
OM857373	EPI_ISL_2839450	hRSV/B/Australia/M236822/2019	ACT	2019-07-27
OM857374	EPI_ISL_2839437	HRSV/B/Australia/WM0331623/2020	NSW	2020-02-02
OM857375	EPI_ISL_2839185	HRSV/B/Australia/WM0663762/2020	NSW	2020-03-06
OM857376	EPI_ISL_2839393	HRSV/B/Australia/WM0715241/2020	NSW	2020-03-11
OM857377	EPI_ISL_2839394	HRSV/B/Australia/WM0573055/2020	NSW	2020-02-26
OM857378	EPI_ISL_2839435	HRSV/B/Australia/WM0711659/2020	NSW	2020-03-11
OM857379	EPI_ISL_2839180	HRSV/B/Australia/WM0531461/2020	NSW	2020-02-22
OM857380	EPI_ISL_2839368	HRSV/B/Australia/WM1603138/2020	NSW	2020-06-08
OM857381	EPI_ISL_2839454	HRSV/B/Australia/WM0636501/2020	NSW	2020-03-03
OM857382	EPI_ISL_2543849	hRSV/B/Australia/VIC-RCH025/2021	VIC	2021-02-20
OM857383	EPI_ISL_2543851	hRSV/B/Australia/VIC-RCH057/2021	VIC	2021-03-06
OM857384	EPI_ISL_2543853	hRSV/B/Australia/VIC-RCH060/2021	VIC	2021-02-22
OM857385	EPI_ISL_2543850	hRSV/B/Australia/VIC-RCH053/2021	VIC	2021-03-07
OM857386	EPI_ISL_2543763	hRSV/B/Australia/VIC-RCH059/2021	VIC	2021-02-22
OM857387	EPI_ISL_2543852	hRSV/B/Australia/VIC-RCH058/2021	VIC	2021-02-21
OM857388	EPI_ISL_2839374	HRSV/B/Australia/WM3181190/2020	NSW	2020-11-13
OM857389	EPI_ISL_2839416	HRSV/B/Australia/WM3181191/2020	NSW	2020-11-13
OM857390	EPI_ISL_2839417	HRSV/B/Australia/WM0322845/2020	NSW	2020-02-01
OM857391	EPI_ISL_2839436	HRSV/B/Australia/WM0543490/2020	NSW	2020-02-23
OM857392	EPI_ISL_2839453	hRSV/B/Australia/9391H/2019	WA	1905-07-11
OM857393	EPI_ISL_2839449	HRSV/B/Australia/WM0782450/2020	NSW	2020-03-18
OM857394	EPI_ISL_2839434	hRSV/B/Australia/M248046/2019	ACT	2019-08-08
OM857395	EPI_ISL_2839193	HRSV/B/Australia/WM0420543/2020	NSW	2020-02-11
OM857396	EPI_ISL_2839366	HRSV/B/Australia/WM0421262/2020	NSW	2020-02-11
OM857397	EPI_ISL_2839442	HRSV/B/Australia/WM0653812/2020	NSW	2020-03-05

*Locations include the Australian Capital Territory (ACT), New South Wales (NSW), Western Australia (WA) and Victoria (VIC).

Supplementary Table 2. Contrast-FEL results with p value < 0.05. Here, hypothesis testing is performed on a site-specific basis using the Likelihood Ratio Test to estimate if non-synonymous substitution rates (dN) along a test branch differs to the dN of reference branches, relative to synonymous substitution rates (dS).

Clade	Gene	Gene position	Site	Partition	alpha	beta (background)	beta (Foreground)	subs (Foreground)	P-value (overall)	Q-value (overall)	Permutation p-value	Total branch length
NSW/ACT/VIC	NS1	9	9	1	0	0	175.687	1	0.001	1	1	0.59
NSW/ACT/VIC	NS2	3	142	1	1.729	0.489	109.44	1	0.005	1	0.05	1.121
NSW/ACT/VIC	G	225	1440	1	1.744	1.42	96.981	1	0.014	1	1	1.67
NSW/ACT/VIC	G	263	1478	1	0	0	331.571	1	0	1	1	1.114
NSW/ACT/VIC	G	265	1480	1	0	1.168	96.068	1	0.014	1	0.05	1.058
NSW/ACT/VIC	G	273	1488	1	0	5.23	143.528	1	0.04	1	1	3.776
NSW/ACT/VIC	G	277	1492	1	8.718	1.312	108.1	1	0.015	1	1	3.437
NSW/ACT/VIC	G	291	1506	1	3.521	0.551	108.494	1	0.006	1	0.2	1.619
NSW/ACT/VIC	G	297	1512	1	0	1.941	143.588	1	0.013	1	0.083	1.705
NSW/ACT/VIC	G	316	1531	1	1.682	0.973	108.226	1	0.009	1	1	1.41
NSW/ACT/VIC	L	755	3058	1	0	0	98.148	1	0.001	0.926	0.2	0.33
WA	N	104	367	1	5.2	0	188.347	1	0.001	1	0.5	1.974
WA	SH	51	1202	1	0	0.49	120.968	1	0.005	1	1	0.715
WA	G	80	1295	1	2.939	0.484	105.505	1	0.005	1	0.2	1.417
WA	G	129	1344	1	0	0	113.439	1	0.001	0.968	1	0.381
WA	G	174	1389	1	0	0	103.368	1	0.001	1	1	0.347

Supplementary Table 3. Results (three replicates) of RELAX analysis for NSW/ACT/VIC and WA clades, which provides alternative model testing using a Likelihood Ratio Test for relaxation or intensification of selection pressure along test branches

Clade	Type	Reference ω_1	Test ω_1	Reference ω_2	Test ω_2	Reference ω_3	Test ω_3	K	P-value	Likelihood ratio
NSW/ACT/ VIC	Intensification	0.00 (3.88%)	0.00 (3.88%)	0.02 (89.99%)	0.00 (89.99%)	1.68 (6.13%)	4.35 (6.13%)	2.82	3.57E-14	57.39
	Intensification	0.00 (66.96%)	0.00 (66.96%)	0.00 (25.17%)	0.00 (25.17%)	1.47 (7.88%)	3.39 (7.88%)	3.15	5.27E-14	56.62
	Intensification	0.04 (90.07%)	0.00 (90.07%)	0.08 (7.33%)	0.00 (7.33%)	2.77 (2.60%)	10.3 (2.60%)	2.28	1.22E-14	59.50
WA	Intensification	0.00 (8.87%)	0.00 (8.87%)	0.10 (90.87%)	0.00 (90.87%)	8.62 (0.26%)	168 (0.26%)	2.38	4.44E-16	66.11
	Intensification	0.00 (18.44%)	0.00 (18.44%)	0.12 (81.30%)	0.01 (81.30%)	8.83 (0.26%)	170 (0.26%)	2.36	4.44E-16	66.29
	Intensification	0.00 (14.82%)	0.00 (14.82%)	0.11 (84.93%)	0.01 (84.93%)	9.17 (0.25%)	172 (0.25%)	2.32	3.33E-16	66.53

Supplementary Table 4. SLAC, SNAP and Contrast-FEL results for NSW/ACT/VIC and WA clades

Clade	SLAC		SNAP		Contrast-FEL	
	dN/dS	Relative rate[b]	dN/dS	Relative rate[b]	dN/dS	Relative rate[b]
NSW/ACT/VIC	0.282	1.373	0.133	1.218	0.266	1.396
WA	0.411	1.697	0.238	1.541	0.378	1.687
Reference[a]	0.134	-	0.062	-	0.116 (0.117)[c]	-

[a]sub-sampled global dataset comprising genomes from major clades.
[b]the ratio of evolution rate of outbreak sequences (NSW/ACT/VIC and WA) versus non-outbreak sequences according to the method proposed by Smith G J D *et al.* (available from https://www.nature.com/articles/nature08182). In this study, the number of non-synonymous sites is ~3.8 times as much as the number of synonymous sites, which was calculated by SNAP.
[c]dN/dS of reference is 0.116 (0.117) when selecting NSW/ACT/VIC (WA) clades as foreground and subsampled global dataset as background.
