# Supplementary material for: Off-season RSV epidemics in Australia after easing of COVID-19 restrictions
Source: Nat Commun. 2022 May 24;13:2884. doi: 10.1038/s41467-022-30485-3 (PMC9130497; doi:10.1038/s41467-022-30485-3)
Supplement: Supplementary file 3 — Description of Additional Supplementary Files [file 41467_2022_30485_MOESM3_ESM.pdf]

Description of Additional Supplementary Files

Supplementary Data 1

Sample list with GenBank & GISAID accession numbers, source locations and collection dates for study samples.

Supplementary Data 2

Sample list with GenBank & GISAID accession numbers, source locations and collection dates for reference sequences sourced from public databases.

Supplementary Data 3

Acknowledgement table for sequences source from GISAID EpiRSV database.
