# Supplementary material for: Off-season RSV epidemics in Australia after easing of COVID-19 restrictions
Source: Nat Commun. 2022 May 24;13:2884. doi: 10.1038/s41467-022-30485-3 (PMC9130497; doi:10.1038/s41467-022-30485-3)
Supplement: Supplementary file 6 — Supplementary Data 3 [file 41467_2022_30485_MOESM6_ESM.pdf]

Description of Additional Supplementary File

Supplementary Data 3

Acknowledgement table for sequences source from GISAID EpiRSV database.

We gratefully acknowledge the following Authors from the Originating laboratories responsible for obtaining the specimens, as well as the Submitting laboratories where the genome data were generated and shared via GISAID, on which this research is based.

All Submitters of data may be contacted directly via www.gisaid.org

Authors are sorted alphabetically.

Accession ID	Originating Laboratory	Submitting Laboratory	Authors
EPI_ISL_1074025, EPI_ISL_1074026, EPI_ISL_1074027, EPI_ISL_1074028, EPI_ISL_1074029, EPI_ISL_1074030, EPI_ISL_1074031, EPI_ISL_1074032, EPI_ISL_1074033, EPI_ISL_1074034, EPI_ISL_1074035, EPI_ISL_1074036, EPI_ISL_1074037, EPI_ISL_1074038, EPI_ISL_1074039, EPI_ISL_1074040, EPI_ISL_1074041, EPI_ISL_1074042, EPI_ISL_1074043, EPI_ISL_1074044, EPI_ISL_1074045, EPI_ISL_1074046, EPI_ISL_1074047, EPI_ISL_1074048, EPI_ISL_1074049, EPI_ISL_1074050, EPI_ISL_1074051, EPI_ISL_1074052, EPI_ISL_1074053, EPI_ISL_1074054, EPI_ISL_1074055, EPI_ISL_1074056, EPI_ISL_1074057, EPI_ISL_1074058, EPI_ISL_1074059, EPI_ISL_1074060, EPI_ISL_1074061, EPI_ISL_1074062, EPI_ISL_1074063, EPI_ISL_1074064, EPI_ISL_1074065, EPI_ISL_1074066, EPI_ISL_1074067, EPI_ISL_1074068, EPI_ISL_1074069, EPI_ISL_1074070, EPI_ISL_1074071, EPI_ISL_1074072, EPI_ISL_1074073, EPI_ISL_1074074, EPI_ISL_1074075, EPI_ISL_1074076, EPI_ISL_1074077, EPI_ISL_1074078, EPI_ISL_1074079, EPI_ISL_1074080, EPI_ISL_1074081, EPI_ISL_1074082, EPI_ISL_1074083, EPI_ISL_1074084, EPI_ISL_1074085, EPI_ISL_1074086, EPI_ISL_1074087, EPI_ISL_1074088, EPI_ISL_1074089, EPI_ISL_1074090, EPI_ISL_1074091, EPI_ISL_1074092, EPI_ISL_1074093, EPI_ISL_1074094, EPI_ISL_1074095, EPI_ISL_1074096, EPI_ISL_1074097, EPI_ISL_1074098, EPI_ISL_1074099, EPI_ISL_1074100, EPI_ISL_1074101, EPI_ISL_1074102, EPI_ISL_1074103, EPI_ISL_1074104, EPI_ISL_1074105, EPI_ISL_1074106, EPI_ISL_1074107, EPI_ISL_1074108, EPI_ISL_1074109, EPI_ISL_1074110, EPI_ISL_1074111, EPI_ISL_1074112, EPI_ISL_1074113, EPI_ISL_1074114, EPI_ISL_1074115, EPI_ISL_1074116, EPI_ISL_1074117, EPI_ISL_1074118, EPI_ISL_1074119, EPI_ISL_1074120, EPI_ISL_1074121, EPI_ISL_1074122, EPI_ISL_1074123, EPI_ISL_1074124, EPI_ISL_1074125, EPI_ISL_1074126, EPI_ISL_1074127, EPI_ISL_1074128, EPI_ISL_1074129, EPI_ISL_1074130, EPI_ISL_1074131, EPI_ISL_1074132, EPI_ISL_1074133, EPI_ISL_1074134, EPI_ISL_1074135, EPI_ISL_1074136, EPI_ISL_1074137, EPI_ISL_1074138, EPI_ISL_1074139, EPI_ISL_1074140, EPI_ISL_1074141, EPI_ISL_1074142, EPI_ISL_1074143, EPI_ISL_1074144, EPI_ISL_1074145, EPI_ISL_1074146, EPI_ISL_1074147, EPI_ISL_1074148, EPI_ISL_1074149, EPI_ISL_1074150, EPI_ISL_1074151, EPI_ISL_1074152, EPI_ISL_1074153, EPI_ISL_1074154, EPI_ISL_1074155, EPI_ISL_1074156, EPI_ISL_1074157, EPI_ISL_1074158, EPI_ISL_1074159, EPI_ISL_1074160, EPI_ISL_1074161, EPI_ISL_1074162, EPI_ISL_1074163, EPI_ISL_1074164, EPI_ISL_1074165, EPI_ISL_1074166, EPI_ISL_1074167, EPI_ISL_1074168, EPI_ISL_1074169, EPI_ISL_1074170, EPI_ISL_1074171, EPI_ISL_1074172, EPI_ISL_1074173, EPI_ISL_1074174, EPI_ISL_1074175, EPI_ISL_1074176, EPI_ISL_1074177, EPI_ISL_1074178, EPI_ISL_1074179, EPI_ISL_1074180, EPI_ISL_1074181, EPI_ISL_1074182, EPI_ISL_1074183, EPI_ISL_1074184, EPI_ISL_1074185, EPI_ISL_1074186, EPI_ISL_1074187, EPI_ISL_1074188, EPI_ISL_1074189, EPI_ISL_1074190, EPI_ISL_1074191, EPI_ISL_1074192, EPI_ISL_1074193, EPI_ISL_1074194, EPI_ISL_1074195, EPI_ISL_1074196, EPI_ISL_1074197, EPI_ISL_1074198, EPI_ISL_1074199, EPI_ISL_1074200, EPI_ISL_1074201, EPI_ISL_1074202, EPI_ISL_1074203, EPI_ISL_1074205, EPI_ISL_1074206, EPI_ISL_1074207, EPI_ISL_1074208, EPI_ISL_1074209, EPI_ISL_1074210, EPI_ISL_1074211, EPI_ISL_1074212, EPI_ISL_1074213, EPI_ISL_1074214, EPI_ISL_1074215, EPI_ISL_1074216, EPI_ISL_1074217, EPI_ISL_1074218, EPI_ISL_1074219, EPI_ISL_1074220, EPI_ISL_1074221, EPI_ISL_1074222, EPI_ISL_1074223, EPI_ISL_1074224, EPI_ISL_1074225, EPI_ISL_1074226, EPI_ISL_1074227, EPI_ISL_1074228, EPI_ISL_1074229, EPI_ISL_1074230, EPI_ISL_1074231, EPI_ISL_1074232, EPI_ISL_1074233, EPI_ISL_1074234, EPI_ISL_1074235, EPI_ISL_1074236, EPI_ISL_1074237, EPI_ISL_1074238, EPI_ISL_1074239, EPI_ISL_1074240, EPI_ISL_1074241, EPI_ISL_1074242, EPI_ISL_1074243, EPI_ISL_1074244, EPI_ISL_1074245, EPI_ISL_1074246, EPI_ISL_1074247, EPI_ISL_1074248, EPI_ISL_1074249, EPI_ISL_1074250, EPI_ISL_1074251, EPI_ISL_1074252, EPI_ISL_1074253, EPI_ISL_1074254, EPI_ISL_1074255, EPI_ISL_1074256, EPI_ISL_1074257, EPI_ISL_1074258, EPI_ISL_1074259, EPI_ISL_1074260, EPI_ISL_1074261, EPI_ISL_1074262, EPI_ISL_1074263, EPI_ISL_1074264, EPI_ISL_1074265, EPI_ISL_1074266, EPI_ISL_1074267, EPI_ISL_1074268, EPI_ISL_1074269, EPI_ISL_1074270, EPI_ISL_1074271, EPI_ISL_1074272, EPI_ISL_1074273, EPI_ISL_1074274, EPI_ISL_1074275, EPI_ISL_1074276, EPI_ISL_1074277, EPI_ISL_1074278, EPI_ISL_1074279, EPI_ISL_1074280, EPI_ISL_1074281, EPI_ISL_1074282, EPI_ISL_1074283, EPI_ISL_1074284, EPI_ISL_1074285, EPI_ISL_1074286, EPI_ISL_1074287, EPI_ISL_1074288, EPI_ISL_1074289, EPI_ISL_1074290, EPI_ISL_1074291, EPI_ISL_1074292, EPI_ISL_1074293, EPI_ISL_1074294, EPI_ISL_1074295, EPI_ISL_1074296, EPI_ISL_1074297, EPI_ISL_1074298, EPI_ISL_1074299, EPI_ISL_1074300			
see above	Virology Laboratory, Ricardo Gutiérrez Children's Hospital	Vanderbilt University Medical Center / Virology Laboratory, Ricardo Gutiérrez Children's Hospital	Goya, Stephanie; Lucion, Maria Florencia; Juarez, Maria del Valle; Shilts, Meghan; Gentile, Angela; Mistchenko, Alicia S.; Das, Suman# & Viegas, Mariana# (#contributed equally to this study)
EPI_ISL_1520381, EPI_ISL_1520382, EPI_ISL_1520383, EPI_ISL_1520384, EPI_ISL_1520385, EPI_ISL_1520386, EPI_ISL_1520387, EPI_ISL_1520388, EPI_ISL_1520389, EPI_ISL_1520390, EPI_ISL_1520391, EPI_ISL_1520392, EPI_ISL_1520393, EPI_ISL_1520394, EPI_ISL_1520395, EPI_ISL_1520396, EPI_ISL_1520397, EPI_ISL_1520398, EPI_ISL_1520399, EPI_ISL_1520400, EPI_ISL_1520401, EPI_ISL_1520402, EPI_ISL_1520403, EPI_ISL_1520404, EPI_ISL_1520405, EPI_ISL_1520406, EPI_ISL_1520407, EPI_ISL_1520408, EPI_ISL_1520409, EPI_ISL_1520410, EPI_ISL_1520411, EPI_ISL_1520412, EPI_ISL_1520413, EPI_ISL_1520414, EPI_ISL_1520415, EPI_ISL_1520416, EPI_ISL_1520417, EPI_ISL_1520418, EPI_ISL_1520419, EPI_ISL_1520420, EPI_ISL_1520421, EPI_ISL_1520422, EPI_ISL_1520423, EPI_ISL_1520424, EPI_ISL_1520425, EPI_ISL_1520426, EPI_ISL_1520427, EPI_ISL_1520428, EPI_ISL_1520429, EPI_ISL_1520430, EPI_ISL_1520431, EPI_ISL_1520432, EPI_ISL_1520433, EPI_ISL_1520434, EPI_ISL_1520435, EPI_ISL_1520436, EPI_ISL_1520437, EPI_ISL_1520438, EPI_ISL_1520439, EPI_ISL_1520440, EPI_ISL_1647383, EPI_ISL_1647384, EPI_ISL_1647385, EPI_ISL_1647386, EPI_ISL_1647387, EPI_ISL_1647388, EPI_ISL_1647389, EPI_ISL_1647390, EPI_ISL_1647391, EPI_ISL_1647392, EPI_ISL_1647393, EPI_ISL_1647394, EPI_ISL_1647395, EPI_ISL_1647396, EPI_ISL_1647397, EPI_ISL_1647398, EPI_ISL_1647399, EPI_ISL_1647400, EPI_ISL_1647401, EPI_ISL_1647402, EPI_ISL_1647403, EPI_ISL_1647404, EPI_ISL_1647405, EPI_ISL_1647406, EPI_ISL_1647407, EPI_ISL_1647408, EPI_ISL_1647409, EPI_ISL_1647410, EPI_ISL_1647411, EPI_ISL_1647412, EPI_ISL_1647413, EPI_ISL_1647414, EPI_ISL_1647415, EPI_ISL_1647416, EPI_ISL_1647417, EPI_ISL_1647418, EPI_ISL_1647419, EPI_ISL_1647420, EPI_ISL_1647421, EPI_ISL_1647422			
see above	Respiratory Virus Unit, National Infection Service, Public Health England	National Infection Service, Public Health England	Zambon M, Talts T, Ellis J, Miah S, Platt S
EPI_ISL_1647456, EPI_ISL_1647457, EPI_ISL_1647458, EPI_ISL_1647459, EPI_ISL_1647460, EPI_ISL_1647461, EPI_ISL_1647462, EPI_ISL_1647463, EPI_ISL_1647464, EPI_ISL_1647465, EPI_ISL_1647466, EPI_ISL_1647467, EPI_ISL_1647468, EPI_ISL_1647469, EPI_ISL_1647470, EPI_ISL_1647471, EPI_ISL_1647472, EPI_ISL_1647473, EPI_ISL_1647474, EPI_ISL_1647475, EPI_ISL_1647476, EPI_ISL_1647477, EPI_ISL_1647478, EPI_ISL_1647479, EPI_ISL_1647480, EPI_ISL_1647481, EPI_ISL_1647482, EPI_ISL_1647483, EPI_ISL_1647484, EPI_ISL_1647485, EPI_ISL_1647486, EPI_ISL_1647487, EPI_ISL_1647488, EPI_ISL_1647489			
see above	Instituto Nacional de Saúde	WHO Influenza Centre for Reference and Research on Influenza	Angela Todd, Yi-Mo Deng, Almiro Rogerio Tivane, Naomi Komadina
EPI_ISL_1647490, EPI_ISL_1647491, EPI_ISL_1647492, EPI_ISL_1647493, EPI_ISL_1647494, EPI_ISL_1647495, EPI_ISL_1647496, EPI_ISL_1647497, EPI_ISL_1647498, EPI_ISL_1647499, EPI_ISL_1647500, EPI_ISL_1647501, EPI_ISL_1647502, EPI_ISL_1647503, EPI_ISL_1647504, EPI_ISL_1647505, EPI_ISL_1647506, EPI_ISL_1647507, EPI_ISL_1647508, EPI_ISL_1647509, EPI_ISL_1647510, EPI_ISL_1647511, EPI_ISL_1647512, EPI_ISL_1647513, EPI_ISL_1647514, EPI_ISL_1647515, EPI_ISL_1647516, EPI_ISL_1647517, EPI_ISL_1647518, EPI_ISL_1647519, EPI_ISL_1647520, EPI_ISL_1647521, EPI_ISL_1647522, EPI_ISL_1647523, EPI_ISL_1647524, EPI_ISL_1647525, EPI_ISL_1647526, EPI_ISL_1647527, EPI_ISL_1647528, EPI_ISL_1647529, EPI_ISL_1647530, EPI_ISL_1647531, EPI_ISL_1647532, EPI_ISL_1647533, EPI_ISL_1647534, EPI_ISL_1647535, EPI_ISL_1647536, EPI_ISL_1647537, EPI_ISL_1647538, EPI_ISL_1647539, EPI_ISL_1647540, EPI_ISL_1647541, EPI_ISL_1647542, EPI_ISL_1647543, EPI_ISL_1647544, EPI_ISL_1647545, EPI_ISL_1647546, EPI_ISL_1647547, EPI_ISL_1647548, EPI_ISL_1647549, EPI_ISL_1647550, EPI_ISL_1647551, EPI_ISL_1647552, EPI_ISL_1647553, EPI_ISL_1647554, EPI_ISL_1647555, EPI_ISL_1647556, EPI_ISL_1647557, EPI_ISL_1647558, EPI_ISL_1647559, EPI_ISL_1647560, EPI_ISL_1647561, EPI_ISL_1647562, EPI_ISL_1647563, EPI_ISL_1647564, EPI_ISL_1647565, EPI_ISL_1647566, EPI_ISL_1647567, EPI_ISL_1647568, EPI_ISL_1647569, EPI_ISL_1647570, EPI_ISL_1647571, EPI_ISL_1647572, EPI_ISL_1647573, EPI_ISL_1647574, EPI_ISL_1647575, EPI_ISL_1647576, EPI_ISL_1647577, EPI_ISL_1647578, EPI_ISL_1647579, EPI_ISL_1647580, EPI_ISL_1647581, EPI_ISL_1647582, EPI_ISL_1647583, EPI_ISL_1647584, EPI_ISL_1647585, EPI_ISL_1647586, EPI_ISL_1647587, EPI_ISL_1647588, EPI_ISL_1647589, EPI_ISL_1647590, EPI_ISL_1647591, EPI_ISL_1647592, EPI_ISL_1647593, EPI_ISL_1647594, EPI_ISL_1647595, EPI_ISL_1647596, EPI_ISL_1647597, EPI_ISL_1647598, EPI_ISL_1647599, EPI_ISL_1647600			
see above	Respiratory Virus Unit, National Infection Service, Public Health England	National Infection Service, Public Health England	Zambon M, Talts T, Ellis J, Miah S, Platt S
EPI_ISL_1653937	Royal Children's Hospital	WHO Influenza Centre for Reference and Research on Influenza	Angela Todd, Yi-Mo Deng, Annette Alafaci, Naomi Komadina
EPI_ISL_1653938, EPI_ISL_1653939	Royal Children's Hospital	WHO Influenza Centre for Reference and Research on Influenza	Jean Moselen, Yi-Mo Deng, Annette Alafaci, Naomi Komadina
EPI_ISL_1653940	Royal Children's Hospital	WHO Influenza Centre for Reference and Research on Influenza	Angela Todd, Yi-Mo Deng, Annette Alafaci, Naomi Komadina
EPI_ISL_1653941, EPI_ISL_1653942	Royal Children's Hospital	WHO Influenza Centre for Reference and Research on Influenza	Jean Moselen, Yi-Mo Deng, Annette Alafaci, Naomi Komadina
EPI_ISL_1653943	Royal Children's Hospital	WHO Influenza Centre for Reference and Research on Influenza	Angela Todd, Yi-Mo Deng, Annette Alafaci, Naomi Komadina
EPI_ISL_1653944	Royal Children's Hospital	WHO Influenza Centre for Reference and Research on Influenza	Jean Moselen, Yi-Mo Deng, Annette Alafaci, Naomi Komadina
EPI_ISL_1653945	Royal Children's Hospital	WHO Influenza Centre for Reference and Research on Influenza	Angela Todd, Yi-Mo Deng, Annette Alafaci, Naomi Komadina
EPI_ISL_1653946	Royal Children's Hospital	WHO Influenza Centre for Reference and Research on Influenza	Jean Moselen, Yi-Mo Deng, Annette Alafaci, Naomi Komadina
EPI_ISL_1653947	Royal Children's Hospital	WHO Influenza Centre for Reference and Research on Influenza	Angela Todd, Yi-Mo Deng, Annette Alafaci, Naomi Komadina
EPI_ISL_1653948	Royal Children's Hospital	WHO Influenza Centre for Reference and Research on Influenza	Jean Moselen, Yi-Mo Deng, Annette Alafaci, Naomi Komadina
EPI_ISL_1653949, EPI_ISL_1653950, EPI_ISL_1653951, EPI_ISL_1653952, EPI_ISL_1653953, EPI_ISL_1653954, EPI_ISL_1653955, EPI_ISL_1653956, EPI_ISL_1653957, EPI_ISL_1653958, EPI_ISL_1653959, EPI_ISL_1653960, EPI_ISL_1653961, EPI_ISL_1653962, EPI_ISL_1653963, EPI_ISL_1653964, EPI_ISL_1653965, EPI_ISL_1653966, EPI_ISL_1653967, EPI_ISL_1653968, EPI_ISL_1653969, EPI_ISL_1653970, EPI_ISL_1653971, EPI_ISL_1653972, EPI_ISL_1653973, EPI_ISL_1653974, EPI_ISL_1653975, EPI_ISL_1653976, EPI_ISL_1653977, EPI_ISL_1653978, EPI_ISL_1653979, EPI_ISL_1653980, EPI_ISL_1653981, EPI_ISL_1653982, EPI_ISL_1653983, EPI_ISL_1653984, EPI_ISL_1653985, EPI_ISL_1653986, EPI_ISL_1653987, EPI_ISL_1653988, EPI_ISL_1653989, EPI_ISL_1653990, EPI_ISL_1653991, EPI_ISL_1653992, EPI_ISL_1653993, EPI_ISL_1653994, EPI_ISL_1653995, EPI_ISL_1653996, EPI_ISL_1653997, EPI_ISL_1653998, EPI_ISL_1653999			
see above	Royal Children's Hospital	WHO Influenza Centre for Reference and Research on Influenza	Angela Todd, Yi-Mo Deng, Annette Alafaci, Naomi Komadina

EPI_ISL_1760383, EPI_ISL_1760384, EPI_ISL_1760385, EPI_ISL_1760386, EPI_ISL_1760387, EPI_ISL_1760388, EPI_ISL_1760389, EPI_ISL_1760390, EPI_ISL_1760391, EPI_ISL_1760392, EPI_ISL_1760393, EPI_ISL_1760394, EPI_ISL_1760395, EPI_ISL_1760396, EPI_ISL_1760397, EPI_ISL_1760398, EPI_ISL_1760399, EPI_ISL_1760400, EPI_ISL_1760401, EPI_ISL_1760402, EPI_ISL_1760403, EPI_ISL_1760404, EPI_ISL_1760405, EPI_ISL_1760406, EPI_ISL_1760407, EPI_ISL_1760408, EPI_ISL_1760409, EPI_ISL_1760410, EPI_ISL_1760411, EPI_ISL_1760412, EPI_ISL_1760413, EPI_ISL_1760414, EPI_ISL_1760415, EPI_ISL_1760416, EPI_ISL_1760417, EPI_ISL_1760418, EPI_ISL_1760419, EPI_ISL_1760420, EPI_ISL_1760421, EPI_ISL_1760422, EPI_ISL_1760423, EPI_ISL_1760424, EPI_ISL_1760425, EPI_ISL_1760426, EPI_ISL_1760427, EPI_ISL_1760428, EPI_ISL_1760429, EPI_ISL_1760430, EPI_ISL_1760431, EPI_ISL_1760432, EPI_ISL_1760433, EPI_ISL_1760434, EPI_ISL_1760435, EPI_ISL_1760436, EPI_ISL_1760437, EPI_ISL_1760438, EPI_ISL_1760439, EPI_ISL_1760440, EPI_ISL_1760441, EPI_ISL_1760442, EPI_ISL_1760443, EPI_ISL_1760444			
see above	Royal Children's Hospital, 50 Flemington Rd., Park	WHO Influenza Centre for Reference and Research on Influenza	Angela Todd, Yi-Mo Deng, Annette Alafaci, Naomi Komadina
EPI_ISL_1834083, EPI_ISL_1834084, EPI_ISL_1834085, EPI_ISL_1834086, EPI_ISL_1834087, EPI_ISL_1834088, EPI_ISL_1834089, EPI_ISL_1834090, EPI_ISL_1834091, EPI_ISL_1834092, EPI_ISL_1834093, EPI_ISL_1834094, EPI_ISL_1834095, EPI_ISL_1834096, EPI_ISL_1834097, EPI_ISL_1834098, EPI_ISL_1834099, EPI_ISL_1834100, EPI_ISL_1834101, EPI_ISL_1834102, EPI_ISL_1834103, EPI_ISL_1834104, EPI_ISL_1834105, EPI_ISL_1834106, EPI_ISL_1834107, EPI_ISL_1834108, EPI_ISL_1834109, EPI_ISL_1834110, EPI_ISL_1834112, EPI_ISL_1834113, EPI_ISL_1834114, EPI_ISL_1834115, EPI_ISL_1834116, EPI_ISL_1834117, EPI_ISL_1834118, EPI_ISL_1834119, EPI_ISL_1834120, EPI_ISL_1834121, EPI_ISL_1834123, EPI_ISL_1834124, EPI_ISL_1834125, EPI_ISL_1834126, EPI_ISL_1834127, EPI_ISL_1834128, EPI_ISL_1834129, EPI_ISL_1834130, EPI_ISL_1834131, EPI_ISL_1834132, EPI_ISL_1834133, EPI_ISL_1834134, EPI_ISL_1834135, EPI_ISL_1834136, EPI_ISL_1834137, EPI_ISL_1834138, EPI_ISL_1834139, EPI_ISL_1834140, EPI_ISL_1834141, EPI_ISL_1834142, EPI_ISL_1834143, EPI_ISL_1834144, EPI_ISL_1834145, EPI_ISL_1834146, EPI_ISL_1834147, EPI_ISL_1834148, EPI_ISL_1834149			
see above	Royal Children's Hospital	WHO Influenza Centre for Reference and Research on Influenza	Angela Todd, Yi-Mo Deng, Annette Alafaci, Naomi Komadina
EPI_ISL_1834150, EPI_ISL_1834151, EPI_ISL_1834152, EPI_ISL_1834153, EPI_ISL_1834154, EPI_ISL_1834155, EPI_ISL_1834158, EPI_ISL_1834159, EPI_ISL_1834160, EPI_ISL_1834161, EPI_ISL_1834162, EPI_ISL_1834163, EPI_ISL_1834164, EPI_ISL_1834165, EPI_ISL_1834166, EPI_ISL_1834167, EPI_ISL_1834168, EPI_ISL_1834169, EPI_ISL_1834170, EPI_ISL_1834171, EPI_ISL_1834172, EPI_ISL_1834173, EPI_ISL_1834174, EPI_ISL_1834175, EPI_ISL_1834176, EPI_ISL_1834177, EPI_ISL_1834178, EPI_ISL_1834179, EPI_ISL_1834180, EPI_ISL_1834181, EPI_ISL_1834182, EPI_ISL_1834183, EPI_ISL_1834184			
see above	Respiratory Virus Unit, National Infection Service, Public Health England	National Infection Service, Public Health England	Zambon M, Talts T, Ellis J, Miah S, Platt S
EPI_ISL_2156812, EPI_ISL_2156813, EPI_ISL_2156814, EPI_ISL_2156815, EPI_ISL_2156816, EPI_ISL_2156817, EPI_ISL_2156818, EPI_ISL_2156819, EPI_ISL_2156820	Royal Children's Hospital	WHO Influenza Centre for Reference and Research on Influenza	Angela Todd, Yi-Mo Deng, Annette Alafaci, Naomi Komadina
EPI_ISL_2543761	National Center for Communicable Diseases	WHO Collaborating Centre for Reference and Research on Influenza	Xiaomin Dong, Darmaa Bardach, Yi-Mo Deng, Ammar Aziz, Naomi Komadina
EPI_ISL_2543762	Monash Medical Centre	WHO Collaborating Centre for Reference and Research on Influenza	Xiaomin Dong, Michelle Francis, Tony Korman, Yi-Mo Deng, Ammar Aziz, Naomi Komadina
EPI_ISL_2543763	Royal Children's Hospital	WHO Collaborating Centre for Reference and Research on Influenza	Xiaomin Dong, Annette Alafaci,Yi-Mo Deng, Ammar Aziz, Naomi Komadina
EPI_ISL_2543764, EPI_ISL_2543765, EPI_ISL_2543766, EPI_ISL_2543767, EPI_ISL_2543768, EPI_ISL_2543769, EPI_ISL_2543770, EPI_ISL_2543771, EPI_ISL_2543772, EPI_ISL_2543773, EPI_ISL_2543774, EPI_ISL_2543775, EPI_ISL_2543776, EPI_ISL_2543777, EPI_ISL_2543778, EPI_ISL_2543779, EPI_ISL_2543780, EPI_ISL_2543781			
see above	Monash Medical Centre	WHO Collaborating Centre for Reference and Research on Influenza	Xiaomin Dong, Michelle Francis, Tony Korman, Yi-Mo Deng, Ammar Aziz, Naomi Komadina
EPI_ISL_2543782, EPI_ISL_2543783, EPI_ISL_2543784, EPI_ISL_2543785, EPI_ISL_2543786, EPI_ISL_2543787, EPI_ISL_2543788, EPI_ISL_2543789, EPI_ISL_2543790, EPI_ISL_2543791, EPI_ISL_2543792, EPI_ISL_2543793, EPI_ISL_2543794, EPI_ISL_2543795, EPI_ISL_2543796, EPI_ISL_2543797, EPI_ISL_2543798, EPI_ISL_2543799, EPI_ISL_2543800, EPI_ISL_2543801, EPI_ISL_2543802, EPI_ISL_2543803, EPI_ISL_2543804, EPI_ISL_2543805, EPI_ISL_2543806, EPI_ISL_2543807, EPI_ISL_2543808, EPI_ISL_2543809, EPI_ISL_2543810			
see above	Royal Children's Hospital	WHO Collaborating Centre for Reference and Research on Influenza	Xiaomin Dong, Annette Alafaci,Yi-Mo Deng, Ammar Aziz, Naomi Komadina
EPI_ISL_2543811, EPI_ISL_2543812, EPI_ISL_2543813, EPI_ISL_2543814, EPI_ISL_2543815, EPI_ISL_2543816, EPI_ISL_2543817, EPI_ISL_2543818, EPI_ISL_2543819, EPI_ISL_2543820, EPI_ISL_2543821, EPI_ISL_2543822, EPI_ISL_2543823, EPI_ISL_2543824, EPI_ISL_2543825, EPI_ISL_2543826			
see above	Institut Pasteur de Cote d'Ivoire	WHO Collaborating Centre for Reference and Research on Influenza	Xiaomin Dong, Herve Kadjo, Yi-Mo Deng, Ammar Aziz, Naomi Komadina
EPI_ISL_2543827, EPI_ISL_2543828, EPI_ISL_2543829, EPI_ISL_2543830, EPI_ISL_2543831, EPI_ISL_2543832, EPI_ISL_2543833, EPI_ISL_2543834, EPI_ISL_2543835, EPI_ISL_2543836, EPI_ISL_2543837, EPI_ISL_2543838, EPI_ISL_2543839, EPI_ISL_2543840, EPI_ISL_2543841, EPI_ISL_2543842, EPI_ISL_2543843, EPI_ISL_2543844			
see above	National Center for Communicable Diseases	WHO Collaborating Centre for Reference and Research on Influenza	Xiaomin Dong, Darmaa Bardach, Yi-Mo Deng, Ammar Aziz, Naomi Komadina
EPI_ISL_2543845, EPI_ISL_2543846, EPI_ISL_2543847	NIC, National Institute of Health	WHO Collaborating Centre for Reference and Research on Influenza	Xiaomin Dong, Pilailuk Akkapaiboon Okada, Yi-Mo Deng, Ammar Aziz, Naomi Komadina
EPI_ISL_2543848	Monash Medical Centre	WHO Collaborating Centre for Reference and Research on Influenza	Xiaomin Dong, Michelle Francis, Tony Korman, Yi-Mo Deng, Ammar Aziz, Naomi Komadina
EPI_ISL_2543849, EPI_ISL_2543850, EPI_ISL_2543851, EPI_ISL_2543852, EPI_ISL_2543853	Royal Children's Hospital	WHO Collaborating Centre for Reference and Research on Influenza	Xiaomin Dong, Annette Alafaci,Yi-Mo Deng, Ammar Aziz, Naomi Komadina
EPI_ISL_2543854, EPI_ISL_2543855, EPI_ISL_2543856, EPI_ISL_2543857, EPI_ISL_2543858, EPI_ISL_2543859	Institut Pasteur de Cote d'Ivoire	WHO Collaborating Centre for Reference and Research on Influenza	Xiaomin Dong, Herve Kadjo, Yi-Mo Deng, Ammar Aziz, Naomi Komadina
EPI_ISL_2543860, EPI_ISL_2543861, EPI_ISL_2543862, EPI_ISL_2543863, EPI_ISL_2543864, EPI_ISL_2543865, EPI_ISL_2543866, EPI_ISL_2543867, EPI_ISL_2543868, EPI_ISL_2543869, EPI_ISL_2543870, EPI_ISL_2543871, EPI_ISL_2543872, EPI_ISL_2543873, EPI_ISL_2543874, EPI_ISL_2543875, EPI_ISL_2543876, EPI_ISL_2543877, EPI_ISL_2543878, EPI_ISL_2543879, EPI_ISL_2543880, EPI_ISL_2543881, EPI_ISL_2543882, EPI_ISL_2543883, EPI_ISL_2543884, EPI_ISL_2543885, EPI_ISL_2543886, EPI_ISL_2543887, EPI_ISL_2543888, EPI_ISL_2543889			
see above	National Center for Communicable Diseases	WHO Collaborating Centre for Reference and Research on Influenza	Xiaomin Dong, Darmaa Bardach, Yi-Mo Deng, Ammar Aziz, Naomi Komadina
EPI_ISL_2543890	NIC, National Institute of Health	WHO Collaborating Centre for Reference and Research on Influenza	Xiaomin Dong, Pilailuk Akkapaiboon Okada, Yi-Mo Deng, Ammar Aziz, Naomi Komadina
EPI_ISL_2543922	KEMRI Wellcome Trust Research Programme	KEMRI Wellcome Trust Research Programme	Agoti,C.N., Otieno,J.R., Munywoki,P.K., Mwihuri,A.G., Cane,P.A., Nokes,D.J., Kellam,P. and Cotten,M.L.
EPI_ISL_2543931	Pathogen Diagnostic Center, Institut Pasteur of Shanghai, Chinese Academy of Sciences	Pathogen Diagnostic Center, Institut Pasteur of Shanghai, Chinese Academy of Sciences	Fu,X., He,Z., Lan,K., Zhang,C., Dong,W., Cheng,Y. and Hu,Y.
EPI_ISL_2543932	Microbiology Department, The University of Hong Kong, Queen Mary Hospital, University Pathology Building	Microbiology Department, The University of Hong Kong, Queen Mary Hospital, University Pathology Building	Zhang,K., He,J., Cheng,Z., Zhou,J., Bose,M., Henrickson,K.J. and Zheng,B.
EPI_ISL_2543933	Influenza Group, National Institute of Virology	Influenza Group, National Institute of Virology	Choudhary,M.L., Wadhwa,B., Jadhav,S.M., Chadha,M.S. and Mourya,D.T.
EPI_ISL_2543934, EPI_ISL_2543935, EPI_ISL_2543936	J. Craig Venter Institute	J. Craig Venter Institute	Shabman,R., Das,S.R., Puri,V., Fedorova,N., Amedeo,P., Williams,M., Shrivastava,S. and Halasa,N.
EPI_ISL_2543937, EPI_ISL_2543938, EPI_ISL_2543939, EPI_ISL_2543940	Virology, Graduate School of Medicine, Tohoku University	Virology, Graduate School of Medicine, Tohoku University	Malasao,R., Furuse,Y., Okamoto,M., Dapat,C., Saito,M., Saito-Obata,M., Tamaki,R., Segubre-Mercado,E., Lupisan,S. and Oshitani,H.
EPI_ISL_2543941	Division of Biosafety Evaluation and Control, Korea National Institute of Health, Korea Centers for Disease Control and Prevention	Division of Biosafety Evaluation and Control, Korea National Institute of Health, Korea Centers for Disease Control and Prevention	Yun,M.-R., Lee,W.-J., Kim,A.-R., Lee,H.S., Kim,K., Kim,S.S., Kim,Y.-J. and Kim,D.-W.
EPI_ISL_2543943	Emerging Viral Infections, Oxford University Clinical Research	Emerging Viral Infections, Oxford University Clinical Research	Do,L.A.H., Wilm,A., van Doorn,H.R., Lam,H.M., Sukumaran,R., Tran,A.T., Nguyen,B.H., Tran,T.T.L., Tran,Q.H., Vo,Q.B., Tran Dac,N.A., Trinh,H.N.,

	Unit	Unit	Nguyen,t.T.H., Le Binh,B.T., Le,K., Nguyen,M.T., Thai,Q.T., Vo,T.V., Ngo,N.Q.M., Dang,t.K.H., Cao,N.H., Tran,T.V., Ho,L.V., Farrar,J., de Jong,M.D., Chen,S., Nagaranjan,N., Bryant,J.E. and Hibberd,M.L.
EPI_ISL_2543949	Medical Microbiology, University Medical Center Utrecht	Medical Microbiology, University Medical Center Utrecht	Tan,L., Lemey,P., Viveen,M. and Coenjaerts,F.E.J.
EPI_ISL_2543952, EPI_ISL_2543953, EPI_ISL_2543954	J. Craig Venter Institute	J. Craig Venter Institute	Lorenzi,H., Town,C., Halpin,R., Bera,J., Ransier,A., Fedorova,N., Stockwell,T., Amedeo,P., Appalla,L., Bishop,B., Edworthy,P., Gupta,N., Hoover,J., Katzel,D., Li,K., Schobel,S., Shrivastava,S., Thovarai,V., Wang,S., Rebuffo-Scheer,C., Fan,J., He,J., Kehl,S.C., Lederboer,N., Jurgens,L.A., Bose,M.E., Beck,E.T., Kumar,S., Gerna,G., Wentworth,D.E. and Henrickson,K.J.
EPI_ISL_2543955	Medical Microbiology, University Medical Center Utrecht	Medical Microbiology, University Medical Center Utrecht	Tan,L., Lemey,P., Viveen,M. and Coenjaerts,F.
EPI_ISL_2543956	Medical Microbiology, University Medical Center Utrecht	Medical Microbiology, University Medical Center Utrecht	Tan,L., Lemey,P., Viveen,M. and Coenjaerts,F.E.J.
EPI_ISL_2543957	Medical Microbiology, University Medical Center Utrecht	Medical Microbiology, University Medical Center Utrecht	Tan,L., Lemey,P., Viveen,M. and Coenjaerts,F.
EPI_ISL_2543958, EPI_ISL_2543959, EPI_ISL_2543960	Medical Microbiology, University Medical Center Utrecht	Medical Microbiology, University Medical Center Utrecht	Tan,L., Lemey,P., Viveen,M. and Coenjaerts,F.E.J.
EPI_ISL_2543967	J. Craig Venter Institute	J. Craig Venter Institute	Lorenzi,H., Town,C., Halpin,R., Bera,J., Ransier,A., Fedorova,N., Stockwell,T., Amedeo,P., Appalla,L., Bishop,B., Edworthy,P., Gupta,N., Hoover,J., Katzel,D., Li,K., Schobel,S., Shrivastava,S., Thovarai,V., Wang,S., Rebuffo-Scheer,C., Fan,J., He,J., Kehl,S.C., Lederboer,N., Jurgens,L.A., Bose,M.E., Beck,E.T., Kumar,S., Noyola,D.E., Wentworth,D.E. and Henrickson,K.J.
EPI_ISL_2543969	Mirus Bio Corporation	Mirus Bio Corporation	Brazas,R.M.
EPI_ISL_2543970, EPI_ISL_2543971, EPI_ISL_2543972, EPI_ISL_2543973, EPI_ISL_2543974, EPI_ISL_2543975, EPI_ISL_2543976, EPI_ISL_2543977, EPI_ISL_2543978, EPI_ISL_2543979, EPI_ISL_2543980, EPI_ISL_2543981, EPI_ISL_2543982			
see above	J. Craig Venter Institute	J. Craig Venter Institute	Das,S.R., Halpin,R.A., Shilts,M., Puri,V., Akopov,A., Fedorova,N., Stockwell,T., Amedeo,P., Bishop,B., Katzel,D., Schobel,S., Shrivastava,S. and Hartert,T.
EPI_ISL_2543983	J. Craig Venter Institute	J. Craig Venter Institute	Shabman,R., Das,S.R., Shilts,M., Fedorova,N., Puri,V., Shrivastava,S., Amedeo,P., Hu,L., Durbin,A., Rocchi,I., Williams,T. and Hartert,T.
EPI_ISL_2543984, EPI_ISL_2543985, EPI_ISL_2543986, EPI_ISL_2543987, EPI_ISL_2543988, EPI_ISL_2543989, EPI_ISL_2543990	Lab Medicine, UW	Lab Medicine, UW	Greninger,A.L., Makhsous,N., Kuypers,J.M., Shean,R.C. and Jerome,K.R.
EPI_ISL_2543991, EPI_ISL_2543992, EPI_ISL_2543993, EPI_ISL_2543995	J. Craig Venter Institute	J. Craig Venter Institute	Das,S.R., Halpin,R.A., Puri,V., Akopov,A., Fedorova,N., Tsitrin,T., Stockwell,T., Amedeo,P., Bishop,B., Gupta,N., Hoover,J., Katzel,D., Schobel,S., Shrivastava,S., Wentworth,D.E. and Caserta,M.
EPI_ISL_2543996, EPI_ISL_2543997, EPI_ISL_2543998, EPI_ISL_2543999, EPI_ISL_2544000, EPI_ISL_2544001, EPI_ISL_2544002, EPI_ISL_2544003	J. Craig Venter Institute	J. Craig Venter Institute	Das,S.R., Halpin,R.A., Puri,V., Akopov,A., Fedorova,N., Stockwell,T., Amedeo,P., Bishop,B., Katzel,D., Schobel,S., Shrivastava,S., Wentworth,D.E. and Caserta,M.
EPI_ISL_2544004, EPI_ISL_2544005, EPI_ISL_2544006, EPI_ISL_2544007, EPI_ISL_2544008, EPI_ISL_2544009, EPI_ISL_2544010, EPI_ISL_2544011, EPI_ISL_2544012, EPI_ISL_2544013	J. Craig Venter Institute	J. Craig Venter Institute	Das,S.R., Halpin,R.A., Puri,V., Akopov,A., Fedorova,N., Stockwell,T., Amedeo,P., Bishop,B., Katzel,D., Schobel,S., Shrivastava,S., Hall,C.B., Tesini,B.L., Schnabel,K.C., Walsh,E.E. and Caserta,M.
EPI_ISL_2544014, EPI_ISL_2544015, EPI_ISL_2544016, EPI_ISL_2544017, EPI_ISL_2544018, EPI_ISL_2544019	J. Craig Venter Institute	J. Craig Venter Institute	Shabman,R., Fedorova,N., Puri,V., Shrivastava,S., Amedeo,P., Isom,R., Hu,L., Pickett,B., Novotny,M., Durbin,A., Rocchi,I., Williams,T., Hall,C.B., Tesini,B.L., Schnabel,K.C., Walsh,E.E. and Caserta,M.
EPI_ISL_2544020	Broad Institute of MIT & Harvard	Broad Institute of MIT & Harvard	Newman,R.M., Zody,M.C., DeVincenzo,J.P., Grad,Y., Lipsitch,M., Murphy,R., Fitzgerald,M., Young,S., Gargeya,S., Poon,T.W., Charlebois,P., Weiner,B., Yang,X., Piper,M.E., McCowan,C., Ireland,A., Levin,J., Malboeuf,C., Qu,J., Chapman,S.B., Murphy,C., Wortman,J., Nusbaum,C. and Birren,B.
EPI_ISL_2544021, EPI_ISL_2544022, EPI_ISL_2544023, EPI_ISL_2544024	J. Craig Venter Institute	J. Craig Venter Institute	Das,S., Halpin,R.A., Bera,J., Fedorova,N., Tsitrin,T., Stockwell,T., Amedeo,P., Bishop,B., Gupta,N., Hoover,J., Katzel,D., Schobel,S., Shrivastava,S., Hartert,T., Moore,M., Chappell,J., Larkin,E., Wentworth,D.E. and Anderson,L.J.
EPI_ISL_2544025	Infectious Diseases, St. Jude Children's Research Hospital	Infectious Diseases, St. Jude Children's Research Hospital	DeVincenzo,J., Jones,B.G., Kim,Y.-I. and Hurwitz,J.L.
EPI_ISL_2544026, EPI_ISL_2544027, EPI_ISL_2544028	J. Craig Venter Institute	J. Craig Venter Institute	Lorenzi,H., Halpin,R., Nguyen,A., Fedorova,N., Overton,L., Stockwell,T., Amedeo,P., Bishop,B., Chen,H., Edworthy,P., Gupta,N., Katzel,D., Li,K., Schobel,S., Shrivastava,S., Thovarai,V., Wang,S., Shapiro,C.A., Williams,J., Peebles,S., Chi,M., Wentworth,D.E. and Moore,M.L.
EPI_ISL_2544029	J. Craig Venter Institute	J. Craig Venter Institute	Lorenzi,H., Town,C., Halpin,R., Bera,J., Ransier,A., Fedorova,N., Stockwell,T., Amedeo,P., Appalla,L., Bishop,B., Edworthy,P., Gupta,N., Hoover,J., Katzel,D., Li,K., Schobel,S., Shrivastava,S., Thovarai,V., Wang,S., Rebuffo-Scheer,C., Fan,J., He,J., Kehl,S.C., Lederboer,N., Jurgens,L.A., Bose,M.E., Beck,E.T., Kumar,S., Wentworth,D.E. and Henrickson,K.J.
EPI_ISL_2544030, EPI_ISL_2544031	Pediatrics - Infectious Diseases, Medical College of Wisconsin	Pediatrics - Infectious Diseases, Medical College of Wisconsin	Rebuffo-Scheer,C., Bose,M.E., He,J., Khaja,S., Ulatowski,M., Beck,E.T., Fan,J., Kumar,S., Nelson,M.I. and Henrickson,K.J.
EPI_ISL_2544032	Molecular Virology and Microbiology, Baylor College of Medicine	Molecular Virology and Microbiology, Baylor College of Medicine	Tapia,L.I. and Piedra,P.A.
EPI_ISL_2544033	J. Craig Venter Institute	J. Craig Venter Institute	Lorenzi,H., Town,C., Halpin,R., Bera,J., Ransier,A., Fedorova,N., Stockwell,T., Amedeo,P., Appalla,L., Bishop,B., Edworthy,P., Gupta,N., Hoover,J., Katzel,D., Li,K., Schobel,S., Shrivastava,S., Thovarai,V., Wang,S., Rebuffo-Scheer,C., Fan,J., He,J., Kehl,S.C., Lederboer,N., Jurgens,L.A., Bose,M.E., Beck,E.T., Kumar,S., Kok,T., Wentworth,D.E. and Henrickson,K.J.
EPI_ISL_2544034, EPI_ISL_2544035, EPI_ISL_2544036, EPI_ISL_2544037, EPI_ISL_2544038, EPI_ISL_2544039, EPI_ISL_2544040, EPI_ISL_2544041	J. Craig Venter Institute	J. Craig Venter Institute	Shabman,R., Das,S.R., Shilts,M., Fedorova,N., Puri,V., Shrivastava,S., Amedeo,P., Williams,M., Barratt,K., Mitchell,J. and Jennings,L.
EPI_ISL_2544044	J. Craig Venter Institute	J. Craig Venter Institute	Lorenzi,H., Town,C., Halpin,R., Bera,J., Ransier,A., Fedorova,N., Stockwell,T., Amedeo,P., Appalla,L., Bishop,B., Edworthy,P., Gupta,N., Hoover,J., Katzel,D., Schobel,S., Shrivastava,S., Thovarai,V., Wang,S., Rebuffo-Scheer,C., Fan,J., He,J., Kehl,S.C., Lederboer,N., Jurgens,L.A., Bose,M.E., Beck,E.T., Kumar,S., Videla,C., Wentworth,D.E. and Henrickson,K.J.
EPI_ISL_2544045, EPI_ISL_2544046	J. Craig Venter Institute	J. Craig Venter Institute	Lorenzi,H., Town,C., Halpin,R., Bera,J., Ransier,A., Fedorova,N., Stockwell,T., Amedeo,P., Appalla,L., Bishop,B., Edworthy,P., Gupta,N., Hoover,J., Katzel,D., Li,K., Schobel,S., Shrivastava,S., Thovarai,V., Wang,S., Rebuffo-Scheer,C., Fan,J., He,J., Kehl,S.C., Lederboer,N., Jurgens,L.A., Bose,M.E., Beck,E.T., Kumar,S., Videla,C., Wentworth,D.E. and Henrickson,K.J.
EPI_ISL_2544051	Virology Laboratory, Dr. Ricardo Gutierrez Children Hospital	Virology Laboratory, Dr. Ricardo Gutierrez Children Hospital	Goya,S., Valinotto,L.E., Tittarelli,E., Rojo,G.L., Greninger,A., Luso,S., Natale,M., Mistchenko,A.S. and Viegas,M.
EPI_ISL_2544052	Virology Laboratory, Dr. Ricardo Gutierrez Children Hospital	Virology Laboratory, Dr. Ricardo Gutierrez Children Hospital	Goya,S., Valinotto,L.E., Tittarelli,E., Rojo,G.L., Greninger,A., Zaiat,J., Marti,M., Mistchenko,A.S. and Viegas,M.
EPI_ISL_2544055, EPI_ISL_2544056, EPI_ISL_2544057, EPI_ISL_2544058, EPI_ISL_2544059, EPI_ISL_2544060, EPI_ISL_2544061, EPI_ISL_2544062, EPI_ISL_2544063, EPI_ISL_2544064	J. Craig Venter Institute	J. Craig Venter Institute	Wentworth,D.E., Halpin,R.A., Bera,J., Lin,X., Fedorova,N., Tsitrin,T., McLellan,M., Stockwell,T., Amedeo,P., Bishop,B., Gupta,N., Hoover,J., Katzel,D., Schobel,S., Shrivastava,S., Garcia,J., Laguna-Torres,V.A., Leguia,M., Benavides,J.G. and Halsey,E.
EPI_ISL_2544066, EPI_ISL_2544067	KEMRI Wellcome Trust Research Programme	KEMRI Wellcome Trust Research Programme	Agoti,C.N., Otieno,J.R., Munywoki,P.K., Mwihuri,A.G., Cane,P.A., Nokes,D.J., Kellam,P. and Cotten,M.L.
EPI_ISL_2544090, EPI_ISL_2544091, EPI_ISL_2544092, EPI_ISL_2544093,	J. Craig Venter Institute	J. Craig Venter Institute	Shabman,R., Das,S.R., Puri,V., Fedorova,N., Amedeo,P., Williams,M., Shrivastava,S. and Halasa,N.

EPI_ISL_2544094			
EPI_ISL_2544100, EPI_ISL_2544101, EPI_ISL_2544102, EPI_ISL_2544103, EPI_ISL_2544104	Emerging Viral Infections, Oxford University Clinical Research Unit	Emerging Viral Infections, Oxford University Clinical Research Unit	Do,L.A.H., Wilm,A., van Doorn,H.R., Lam,H.M., Sukumaran,R., Tran,A.T., Nguyen,B.H., Tran,T.T.L., Tran,Q.H., Vo,Q.B., Tran Dac,N.A., Trinh,H.N., Nguyen,t.T.H., Le Binh,B.T., Le,K., Nguyen,M.T., Thai,Q.T., Vo,T.V., Ngo,N.Q.M., Dang,t.K.H., Cao,N.H., Tran,T.V., Ho,L.V., Farrar,J., de Jong,M.D., Chen,S., Nagaranjan,N., Bryant,J.E. and Hibberd,M.L.
EPI_ISL_2544106	Medical Microbiology, University Medical Center Utrecht	Medical Microbiology, University Medical Center Utrecht	Tan,L., Viveen,M.C., Lemey,P. and Coenjaerts,F.E.
EPI_ISL_2544107	J. Craig Venter Institute	J. Craig Venter Institute	Lorenzi,H., Town,C., Halpin,R., Bera,J., Ransier,A., Fedorova,N., Stockwell,T., Amedeo,P., Appalla,L., Bishop,B., Edworthy,P., Gupta,N., Hoover,J., Katzel,D., Li,K., Schobel,S., Shrivastava,S., Thovarai,V., Wang,S., Rebuffo-Scheer,C., Fan,J., He,J., Kehl,S.C., Lederboer,N., Jurgens,L.A., Bose,M.E., Beck,E.T., Kumar,S., Neumann-Haefelin,D., Wentworth,D.E. and Henrickson,K.J.
EPI_ISL_2544108	J. Craig Venter Institute	J. Craig Venter Institute	Lorenzi,H., Town,C., Halpin,R., Bera,J., Ransier,A., Fedorova,N., Stockwell,T., Amedeo,P., Appalla,L., Bishop,B., Edworthy,P., Gupta,N., Hoover,J., Katzel,D., Li,K., Schobel,S., Shrivastava,S., Thovarai,V., Wang,S., Rebuffo-Scheer,C., Fan,J., He,J., Kehl,S.C., Lederboer,N., Jurgens,L.A., Bose,M.E., Beck,E.T., Kumar,S., Gerna,G., Wentworth,D.E. and Henrickson,K.J.
EPI_ISL_2544109, EPI_ISL_2544110, EPI_ISL_2544111, EPI_ISL_2544112, EPI_ISL_2544113, EPI_ISL_2544114, EPI_ISL_2544115	Medical Microbiology, University Medical Center Utrecht	Medical Microbiology, University Medical Center Utrecht	Tan,L., Viveen,M.C., Lemey,P. and Coenjaerts,F.E.
EPI_ISL_2544121, EPI_ISL_2544122, EPI_ISL_2544123, EPI_ISL_2544124, EPI_ISL_2544125, EPI_ISL_2544126	Reference Microbiology, Public Health England National Infection Services	Reference Microbiology, Public Health England National Infection Services	Valappil,M., Talts,T., Ellis,J., Sails,A., Eltringham,G., Waugh,S., Gould,K., Harrison,I., Pebody,R. and Zambon,M.
EPI_ISL_2544137, EPI_ISL_2544138	J. Craig Venter Institute	J. Craig Venter Institute	Lorenzi,H., Town,C., Halpin,R., Bera,J., Ransier,A., Fedorova,N., Stockwell,T., Amedeo,P., Appalla,L., Bishop,B., Edworthy,P., Gupta,N., Hoover,J., Katzel,D., Li,K., Schobel,S., Shrivastava,S., Thovarai,V., Wang,S., Rebuffo-Scheer,C., Fan,J., He,J., Kehl,S.C., Lederboer,N., Jurgens,L.A., Bose,M.E., Beck,E.T., Kumar,S., Noyola,D.E., Wentworth,D.E. and Henrickson,K.J.
EPI_ISL_2544141, EPI_ISL_2544142, EPI_ISL_2544143, EPI_ISL_2544144, EPI_ISL_2544145, EPI_ISL_2544146	J. Craig Venter Institute	J. Craig Venter Institute	Das,S.R., Halpin,R.A., Shilts,M., Puri,V., Akopov,A., Fedorova,N., Stockwell,T., Amedeo,P., Bishop,B., Katzel,D., Schobel,S., Shrivastava,S. and Hartert,T.
EPI_ISL_2544147	Lab Medicine, UW	Lab Medicine, UW	Greninger,A.L., Makhsous,N., Kuypers,J.M., Shean,R.C. and Jerome,K.R.
EPI_ISL_2544148, EPI_ISL_2544149, EPI_ISL_2544150	Pediatrics, UT Southwestern Medical Center	Pediatrics, UT Southwestern Medical Center	Levitz,R., Gao,Y., Dozmorov,I., Song,R., Wakeland,E.K. and Kahn,J.S.
EPI_ISL_2544151	MedImmune	MedImmune	Cheng,X., Park,H. and Jin,H.
EPI_ISL_2544152	St. Louis University Medical Center	Viral Vaccine Research	R. Belshe, Karron,R.A., Buonagurio,D.A., Georgiu,A.F., Whitehead,S.S., Adamus,J.E., Clements-Mann,M.L., Harris,D.O., Randolph,V.B., Udem,S.A., Murphy,B.R. and Sidhu,M.S.
EPI_ISL_2544153, EPI_ISL_2544154, EPI_ISL_2544155, EPI_ISL_2544156	J. Craig Venter Institute	J. Craig Venter Institute	Das,S.R., Halpin,R.A., Puri,V., Akopov,A., Fedorova,N., Stockwell,T., Amedeo,P., Bishop,B., Katzel,D., Schobel,S., Shrivastava,S., Wentworth,D.E. and Caserta,M.
EPI_ISL_2544157, EPI_ISL_2544158, EPI_ISL_2544159, EPI_ISL_2544160, EPI_ISL_2544161, EPI_ISL_2544162	J. Craig Venter Institute	J. Craig Venter Institute	Das,S.R., Halpin,R.A., Puri,V., Akopov,A., Fedorova,N., Stockwell,T., Amedeo,P., Bishop,B., Katzel,D., Schobel,S., Shrivastava,S., Hall,C.B., Tesini,B.L., Schnabel,K.C., Walsh,E.E. and Caserta,M.
EPI_ISL_2544163, EPI_ISL_2544164, EPI_ISL_2544165	J. Craig Venter Institute	J. Craig Venter Institute	Shabman,R., Fedorova,N., Puri,V., Shrivastava,S., Amedeo,P., Isom,R., Hu,L., Pickett,B., Novotny,M., Durbin,A., Rocchi,I., Williams,T., Hall,C.B., Tesini,B.L., Schnabel,K.C., Walsh,E.E. and Caserta,M.
EPI_ISL_2544171, EPI_ISL_2544172	Pediatrics - Infectious Diseases, Medical College of Wisconsin	Pediatrics - Infectious Diseases, Medical College of Wisconsin	Rebuffo-Scheer,C., Bose,M.E., He,J., Khaja,S., Ulatowski,M., Beck,E.T., Fan,J., Kumar,S., Nelson,M.I. and Henrickson,K.J.
EPI_ISL_2544173, EPI_ISL_2544174, EPI_ISL_2544175, EPI_ISL_2544176, EPI_ISL_2544177, EPI_ISL_2544178	J. Craig Venter Institute	J. Craig Venter Institute	Shabman,R., Das,S.R., Shilts,M., Fedorova,N., Puri,V., Shrivastava,S., Amedeo,P., Williams,M., Barratt,K., Mitchell,J. and Jennings,L.
EPI_ISL_2544180, EPI_ISL_2544181	J. Craig Venter Institute	J. Craig Venter Institute	Lorenzi,H., Town,C., Halpin,R., Bera,J., Ransier,A., Fedorova,N., Stockwell,T., Amedeo,P., Appalla,L., Bishop,B., Edworthy,P., Gupta,N., Hoover,J., Katzel,D., Li,K., Schobel,S., Shrivastava,S., Thovarai,V., Wang,S., Rebuffo-Scheer,C., Fan,J., He,J., Kehl,S.C., Lederboer,N., Jurgens,L.A., Bose,M.E., Beck,E.T., Kumar,S., Videla,C., Wentworth,D.E. and Henrickson,K.J.
EPI_ISL_2544184	Virology Laboratory, Dr. Ricardo Gutierrez Children Hospital	Virology Laboratory, Dr. Ricardo Gutierrez Children Hospital	Goya,S., Valinotto,L.E., Tittarelli,E., Rojo,G.L., Greninger,A., Luso,S., Natale,M., Mistchenko,A.S. and Viegas,M.
EPI_ISL_2544185	Virology Laboratory, Dr. Ricardo Gutierrez Children Hospital	Virology Laboratory, Dr. Ricardo Gutierrez Children Hospital	Goya,S., Valinotto,L.E., Tittarelli,E., Rojo,G.L., Greninger,A., Zaiat,J., Marti,M., Mistchenko,A.S. and Viegas,M.
EPI_ISL_2544186	Virology Laboratory, Dr. Ricardo Gutierrez Children Hospital	Virology Laboratory, Dr. Ricardo Gutierrez Children Hospital	Goya,S., Rojo,G.L., Valinotto,L.E., Mistchenko,A.S. and Viegas,M.
EPI_ISL_2544188, EPI_ISL_2544189	Microbiology, Institute of Biological Sciences, Universeity of Sao Paulo	Microbiology, Institute of Biological Sciences, Universeity of Sao Paulo	Di Paola,N., Cunha,M.P., Oliveira,D.B.L., Durigon,E., Durigon,G.S. and Zanotto,P.M.A.
EPI_ISL_2544190, EPI_ISL_2544191, EPI_ISL_2544192	J. Craig Venter Institute	J. Craig Venter Institute	Wentworth,D.E., Halpin,R.A., Bera,J., Lin,X., Fedorova,N., Tsitrin,T., McLellan,M., Stockwell,T., Amedeo,P., Bishop,B., Gupta,N., Hoover,J., Katzel,D., Schobel,S., Shrivastava,S., Garcia,J., Laguna-Torres,V.A., Leguia,M., Benavides,J.G. and Halsey,E.
EPI_ISL_2558779	Kazuya Shirato National Institute of Infectious Diseases, Virology III	Kazuya Shirato National Institute of Infectious Diseases, Virology III	Shirato,K., Sato,K., Dapat,I., Nao,N., Omiya,S., Matsuyama,S., Takeda,M. and Nishimura,H.
EPI_ISL_2558780, EPI_ISL_2558781, EPI_ISL_2558782	Pediatrics - Infectious Diseases, Medical College of Wisconsin	Pediatrics - Infectious Diseases, Medical College of Wisconsin	Rebuffo-Scheer,C., Bose,M.E., He,J., Khaja,S., Ulatowski,M., Beck,E.T., Fan,J., Kumar,S., Nelson,M.I. and Henrickson,K.J.
EPI_ISL_2558785, EPI_ISL_2558786, EPI_ISL_2558799	Kazuya Shirato National Institute of Infectious Diseases, Virology III	Kazuya Shirato National Institute of Infectious Diseases, Virology III	Shirato,K., Sato,K., Dapat,I., Nao,N., Omiya,S., Matsuyama,S., Takeda,M. and Nishimura,H.
EPI_ISL_2558800	J. Craig Venter Institute	J. Craig Venter Institute	Shabman,R., Fedorova,N., Puri,V., Shrivastava,S., Amedeo,P., Isom,R., Hu,L., Pickett,B., Novotny,M., Durbin,A., Rocchi,I., Williams,T., Hall,C.B., Tesini,B.L., Schnabel,K.C., Walsh,E.E. and Caserta,M.
EPI_ISL_2558884	Kazuya Shirato National Institute of Infectious Diseases, Virology III	Kazuya Shirato National Institute of Infectious Diseases, Virology III	Shirato,K., Sato,K., Dapat,I., Nao,N., Omiya,S., Matsuyama,S., Takeda,M. and Nishimura,H.
EPI_ISL_2558885	Pediatrics - Infectious Diseases, Medical College of Wisconsin	Pediatrics - Infectious Diseases, Medical College of Wisconsin	Rebuffo-Scheer,C., Bose,M.E., He,J., Khaja,S., Ulatowski,M., Beck,E.T., Fan,J., Kumar,S., Nelson,M.I. and Henrickson,K.J.
EPI_ISL_2558886, EPI_ISL_2558887, EPI_ISL_2558888, EPI_ISL_2558895, EPI_ISL_2558896, EPI_ISL_2558897, EPI_ISL_2558898, EPI_ISL_2558899, EPI_ISL_2558900, EPI_ISL_2558901, EPI_ISL_2558902, EPI_ISL_2558903, EPI_ISL_2558904, EPI_ISL_2558905, EPI_ISL_2558907, EPI_ISL_2558908			
see above	Kazuya Shirato National Institute of Infectious Diseases, Virology III	Kazuya Shirato National Institute of Infectious Diseases, Virology III	Shirato,K., Sato,K., Dapat,I., Nao,N., Omiya,S., Matsuyama,S., Takeda,M. and Nishimura,H.
EPI_ISL_2558919	J. Craig Venter Institute	J. Craig Venter Institute	Shabman,R., Fedorova,N., Puri,V., Shrivastava,S., Amedeo,P., Isom,R., Hu,L., Pickett,B., Novotny,M., Durbin,A., Rocchi,I., Williams,T., Hall,C.B., Tesini,B.L., Schnabel,K.C., Walsh,E.E. and Caserta,M.
EPI_ISL_2558924, EPI_ISL_2558925, EPI_ISL_2558926	Kazuya Shirato National Institute of Infectious Diseases, Virology III	Kazuya Shirato National Institute of Infectious Diseases, Virology III	Shirato,K., Sato,K., Dapat,I., Nao,N., Omiya,S., Matsuyama,S., Takeda,M. and Nishimura,H.
EPI_ISL_2558927	Lab Medicine, UW	Lab Medicine, UW	Greninger,A.L., Makhsous,N., Kuypers,J.M., Shean,R.C. and Jerome,K.R.

EPI_ISL_2558934, EPI_ISL_2558935, EPI_ISL_2558936	J. Craig Venter Institute	J. Craig Venter Institute	Shabman,R., Fedorova,N., Puri,V., Shrivastava,S., Amedeo,P., Isom,R., Hu,L., Pickett,B., Novotny,M., Durbin,A., Rocchi,I., Williams,T., Hall,C.B., Tesini,B.L., Schnabel,K.C., Walsh,E.E. and Caserta,M.
EPI_ISL_2558971	Lab Medicine, UW	Lab Medicine, UW	Greninger,A.L., Makhsous,N., Kuypers,J.M., Shean,R.C. and Jerome,K.R.
EPI_ISL_2558973, EPI_ISL_2558974, EPI_ISL_2558975, EPI_ISL_2558976	J. Craig Venter Institute	J. Craig Venter Institute	Shabman,R., Fedorova,N., Puri,V., Shrivastava,S., Amedeo,P., Isom,R., Hu,L., Pickett,B., Novotny,M., Durbin,A., Rocchi,I., Williams,T., Hall,C.B., Tesini,B.L., Schnabel,K.C., Walsh,E.E. and Caserta,M.
EPI_ISL_2558985	Koichi Hashimoto Fukushima Medical University, Department of Pediatrics, School of Medicine	Koichi Hashimoto Fukushima Medical University, Department of Pediatrics, School of Medicine	Fukuyama,Y., Takeshita,F., Norito,S., Hashimoto,K. and Hosoya,M.
EPI_ISL_2558987	Department of Infectious Diseases and Pathobiology, Institute of Virology and Immunology (IVI)	Department of Infectious Diseases and Pathobiology, Institute of Virology and Immunology (IVI)	Thao,T.T.N., Labroussaa,F., Ebert,N., Stalder,H., Dijkman,R., Jores,J., Thiel,V., Bittel,P., Suter-Riniker,F. and Kelly,J.
EPI_ISL_2559009, EPI_ISL_2559010, EPI_ISL_2559011	J. Craig Venter Institute	J. Craig Venter Institute	Shabman,R., Fedorova,N., Puri,V., Shrivastava,S., Amedeo,P., Isom,R., Hu,L., Pickett,B., Novotny,M., Durbin,A., Rocchi,I., Williams,T., Hall,C.B., Tesini,B.L., Schnabel,K.C., Walsh,E.E. and Caserta,M.
EPI_ISL_2559012	Center for Infectious Diseases, School of Public Health, University of Texas Health Science Center	Center for Infectious Diseases, School of Public Health, University of Texas Health Science Center	Bahl,J., Hixson,J., Kim,D.-K., Qiu,X., Piedra,P.A., Piedra,F.-A., Avadhanula,V. and Machado,A.A.
EPI_ISL_2559013, EPI_ISL_2559014, EPI_ISL_2559015, EPI_ISL_2559016, EPI_ISL_2559017, EPI_ISL_2559018, EPI_ISL_2559019, EPI_ISL_2559069, EPI_ISL_2559070, EPI_ISL_2559071	J. Craig Venter Institute	J. Craig Venter Institute	Shabman,R., Fedorova,N., Puri,V., Shrivastava,S., Amedeo,P., Isom,R., Hu,L., Pickett,B., Novotny,M., Durbin,A., Rocchi,I., Williams,T., Hall,C.B., Tesini,B.L., Schnabel,K.C., Walsh,E.E. and Caserta,M.
EPI_ISL_2559073	Kazuya Shirato National Institute of Infectious Diseases, Virology III	Kazuya Shirato National Institute of Infectious Diseases, Virology III	Shirato,K., Sato,K., Dapat,I., Nao,N., Omiya,S., Matsuyama,S., Takeda,M. and Nishimura,H.
EPI_ISL_2559080	Pediatrics - Infectious Diseases, Medical College of Wisconsin	Pediatrics - Infectious Diseases, Medical College of Wisconsin	Rebuffo-Scheer,C., Bose,M.E., He,J., Khaja,S., Ulatowski,M., Beck,E.T., Fan,J., Kumar,S., Nelson,M.I. and Henrickson,K.J.
EPI_ISL_2559081, EPI_ISL_2560801, EPI_ISL_2560802	Kazuya Shirato National Institute of Infectious Diseases, Virology III	Kazuya Shirato National Institute of Infectious Diseases, Virology III	Shirato,K., Sato,K., Dapat,I., Nao,N., Omiya,S., Matsuyama,S., Takeda,M. and Nishimura,H.
EPI_ISL_2560803	Lab Medicine, UW	Lab Medicine, UW	Greninger,A.L., Makhsous,N., Kuypers,J.M., Shean,R.C. and Jerome,K.R.
EPI_ISL_2560804	Virology, University of Washington	Virology, University of Washington	Greninger,A.L., Shean,R.C. and Makhsous,N.
EPI_ISL_2560805	Laboratory Medicine, UW Virology	Laboratory Medicine, UW Virology	Greninger,A.L., Tait,A. and Makhsous,N.
EPI_ISL_2560806	Lab Medicine, UW	Lab Medicine, UW	Greninger,A.L., Makhsous,N., Kuypers,J.M., Shean,R.C. and Jerome,K.R.
EPI_ISL_2560807	Kazuya Shirato National Institute of Infectious Diseases, Virology III	Kazuya Shirato National Institute of Infectious Diseases, Virology III	Shirato,K., Sato,K., Dapat,I., Nao,N., Omiya,S., Matsuyama,S., Takeda,M. and Nishimura,H.
EPI_ISL_2560808, EPI_ISL_2560809, EPI_ISL_2560815, EPI_ISL_2560837	Lab Medicine, UW	Lab Medicine, UW	Greninger,A.L., Makhsous,N., Kuypers,J.M., Shean,R.C. and Jerome,K.R.
EPI_ISL_2560838	Kazuya Shirato National Institute of Infectious Diseases, Virology III	Kazuya Shirato National Institute of Infectious Diseases, Virology III	Shirato,K., Sato,K., Dapat,I., Nao,N., Omiya,S., Matsuyama,S., Takeda,M. and Nishimura,H.
EPI_ISL_2560839	Lab Medicine, UW	Lab Medicine, UW	Greninger,A.L., Makhsous,N., Kuypers,J.M., Shean,R.C. and Jerome,K.R.
EPI_ISL_2561008, EPI_ISL_2561009, EPI_ISL_2561010	Kazuya Shirato National Institute of Infectious Diseases, Virology III	Kazuya Shirato National Institute of Infectious Diseases, Virology III	Shirato,K., Sato,K., Dapat,I., Nao,N., Omiya,S., Matsuyama,S., Takeda,M. and Nishimura,H.
EPI_ISL_2561401	Lab Medicine, UW	Lab Medicine, UW	Greninger,A.L., Makhsous,N., Kuypers,J.M., Shean,R.C. and Jerome,K.R.
EPI_ISL_2561453	Maximum Containment Laboratory, National Institue of Virology	Maximum Containment Laboratory, National Institue of Virology	Yadav,P.D.
EPI_ISL_2574728	Epidemiology and Demography Department, KEMRI-Wellcome Trust Research Programme	Epidemiology and Demography Department, KEMRI-Wellcome Trust Research Programme	Otieno,J.R., Kamau,E.M., Oketch,J.W., Ngoi,J.M., Agoti,C.N., Gichuki,A.M., Otieno,G.P., Ngama,M., Cane,P.A., Kellam,P., Cotten,M., Lemey,P. and Nokes,D.J.
EPI_ISL_2575367	Suguru Takeuchi Nagoya University Graduate School of Medicine, Pediatrics	Suguru Takeuchi Nagoya University Graduate School of Medicine, Pediatrics	Takeuchi,S., Kawada,J. and Ito,Y.
EPI_ISL_2575413	Pediatrics - Infectious Diseases, Medical College of Wisconsin	Pediatrics - Infectious Diseases, Medical College of Wisconsin	Rebuffo-Scheer,C., Bose,M.E., He,J., Khaja,S., Ulatowski,M., Beck,E.T., Fan,J., Kumar,S., Nelson,M.I. and Henrickson,K.J.
EPI_ISL_2575417	Kazuya Shirato National Institute of Infectious Diseases, Virology III	Kazuya Shirato National Institute of Infectious Diseases, Virology III	Shirato,K., Sato,K., Dapat,I., Nao,N., Omiya,S., Matsuyama,S., Takeda,M. and Nishimura,H.
EPI_ISL_2575423	J. Craig Venter Institute	J. Craig Venter Institute	Tan,G., Pickett,B., Fedorova,N., Amedeo,P., Hu,L., Christensen,J., Miller,J., Durbin,A., Williams,T., Arumemi,F., Cadiz,C., Alanis,R., Balmseda,A., Wiliams,T., Schiller,A., Patel,M., Kubale,J. and Gordon,A.
EPI_ISL_2575424	Marie Bashir Institute for Infectious Diseases and Biosecurity & Sydney Medical School, The University of Sydney, Westmead Institute for Medical Research	Marie Bashir Institute for Infectious Diseases and Biosecurity & Sydney Medical School, The University of Sydney, Westmead Institute for Medical Research	Eden,J.-S., Kok,J., Dwyer,D.E., Fernandez,M., Carter,I. and Holmes,E.C.
EPI_ISL_2575425	Virus Research Group, Beijing Pediatric Research Institute, Beijing Children's Hospital, Capital Medical University	Virus Research Group, Beijing Pediatric Research Institute, Beijing Children's Hospital, Capital Medical University	Xu,L. and Xie,Z.
EPI_ISL_2575427, EPI_ISL_2575428, EPI_ISL_2575429, EPI_ISL_2575430	J. Craig Venter Institute	J. Craig Venter Institute	Tan,G., Pickett,B., Fedorova,N., Amedeo,P., Hu,L., Christensen,J., Miller,J., Durbin,A., Williams,T., Arumemi,F., Cadiz,C., Alanis,R., Balmseda,A., Wiliams,T., Schiller,A., Patel,M., Kubale,J. and Gordon,A.
EPI_ISL_2575431	Reference Microbiology, Public Health England National Infection Services	Reference Microbiology, Public Health England National Infection Services	Valappil,M., Talts,T., Ellis,J., Sails,A., Eltringham,G., Waugh,S., Gould,K., Harrison,I., Pebody,R. and Zambon,M.
EPI_ISL_2575432	Marie Bashir Institute for Infectious Diseases and Biosecurity & Sydney Medical School, The University of Sydney, Westmead Institute for Medical Research	Marie Bashir Institute for Infectious Diseases and Biosecurity & Sydney Medical School, The University of Sydney, Westmead Institute for Medical Research	Eden,J.-S., Kok,J., Dwyer,D.E., Fernandez,M., Carter,I. and Holmes,E.C.
EPI_ISL_2575463	J. Craig Venter Institute	J. Craig Venter Institute	Tan,G., Pickett,B., Fedorova,N., Amedeo,P., Hu,L., Christensen,J., Miller,J., Durbin,A., Williams,T., Arumemi,F., Cadiz,C., Alanis,R., Balmseda,A., Wiliams,T., Schiller,A., Patel,M., Kubale,J. and Gordon,A.
EPI_ISL_2575465	Pediatrics, University of New Mexico	Pediatrics, University of New Mexico	Dinwiddle,D.
EPI_ISL_2575471	Michiko Okamoto Graduate School of Medicine, Tohoku University, Virology	Michiko Okamoto Graduate School of Medicine, Tohoku University, Virology	Okamoto,M. and Oshitani,H.
EPI_ISL_2575474, EPI_ISL_2575475, EPI_ISL_2575476, EPI_ISL_2575478, EPI_ISL_2575479, EPI_ISL_2575480, EPI_ISL_2575481, EPI_ISL_2575482, EPI_ISL_2575483, EPI_ISL_2575484, EPI_ISL_2575485, EPI_ISL_2575486, EPI_ISL_2575487, EPI_ISL_2575488, EPI_ISL_2575489			
see above	Epidemiology and Demography Department, KEMRI Wellcome Trust Research Collaborative Programme	Epidemiology and Demography Department, KEMRI Wellcome Trust Research Collaborative Programme	Agoti,C.N., Phan,M.V.T., Munywoki,P.K., Githinji,G., Medley,G.F., Cane,P.A., Kellam,K., Cotten,M. and Nokes,D.J.
EPI_ISL_2575490	Marie Bashir Institute for Infectious Diseases and Biosecurity	Marie Bashir Institute for Infectious Diseases and Biosecurity	Eden,J.-S., Kok,J., Dwyer,D.E., Fernandez,M., Carter,I. and Holmes,E.C.

	& Sydney Medical School, The University of Sydney, Westmead Institute for Medical Research	& Sydney Medical School, The University of Sydney, Westmead Institute for Medical Research	
EPI_ISL_2575491	J. Craig Venter Institute	J. Craig Venter Institute	Tan,G., Pickett,B., Fedorova,N., Amedeo,P., Hu,L., Christensen,J., Miller,J., Durbin,A., Williams,T., Arumemi,F., Cadiz,C., Alanis,R., Balmseda,A., Wiliams,T., Schiller,A., Patel,M., Kubale,J. and Gordon,A.
EPI_ISL_2575492	Reference Microbiology, Public Health England National Infection Services	Reference Microbiology, Public Health England National Infection Services	Valappil,M., Talts,T., Ellis,J., Sails,A., Eltringham,G., Waugh,S., Gould,K., Harrison,I., Pebody,R. and Zambon,M.
EPI_ISL_2575493	J. Craig Venter Institute	J. Craig Venter Institute	Tan,G., Pickett,B., Fedorova,N., Amedeo,P., Isom,R., Hu,L., Christensen,J., Miller,J., Novotny,M., Durbin,A., Rocchi,I., Williams,T., Arumemi,F. and Das,S.
EPI_ISL_2575494, EPI_ISL_2575495	Marie Bashir Institute for Infectious Diseases and Biosecurity & Sydney Medical School, The University of Sydney, Westmead Institute for Medical Research	Marie Bashir Institute for Infectious Diseases and Biosecurity & Sydney Medical School, The University of Sydney, Westmead Institute for Medical Research	Eden,J.-S., Kok,J., Dwyer,D.E., Fernandez,M., Carter,I. and Holmes,E.C.
EPI_ISL_2575496, EPI_ISL_2575497	Pediatrics, University of New Mexico	Pediatrics, University of New Mexico	Dinwiddle,D.
EPI_ISL_2575498	J. Craig Venter Institute	J. Craig Venter Institute	Tan,G., Pickett,B., Fedorova,N., Amedeo,P., Hu,L., Christensen,J., Miller,J., Durbin,A., Williams,T., Arumemi,F., Cadiz,C., Alanis,R., Balmseda,A., Wiliams,T., Schiller,A., Patel,M., Kubale,J. and Gordon,A.
EPI_ISL_2575499	Department of Experimental Modeling and Infectious Diseases Pathogenesis, Federal Research Center of Fundamental and Translational Medicine	Department of Experimental Modeling and Infectious Diseases Pathogenesis, Federal Research Center of Fundamental and Translational Medicine	Kurskaya,O.G., Sobolev,I.A., Sharshov,K.A., Alexeev,A.Y., Murashkina,T.A., Kabilov,M.R., Alikina,T.Y. and Shestopalov,A.M.
EPI_ISL_2575500	Department of Experimental Modeling and Infectious Diseases Pathogenesis, Federal Research Center of Fundamental and Translational Medicine	Department of Experimental Modeling and Infectious Diseases Pathogenesis, Federal Research Center of Fundamental and Translational Medicine	Sobolev,I.A., Kurskaya,O.G., Sharshov,K.A., Alexeev,A.Y., Murashkina,T.A., Kabilov,M.R., Alikina,T.Y. and Shestopalov,A.M.
EPI_ISL_2575505, EPI_ISL_2575506	Marie Bashir Institute for Infectious Diseases and Biosecurity & Sydney Medical School, The University of Sydney, Westmead Institute for Medical Research	Marie Bashir Institute for Infectious Diseases and Biosecurity & Sydney Medical School, The University of Sydney, Westmead Institute for Medical Research	Eden,J.-S., Kok,J., Dwyer,D.E., Fernandez,M., Carter,I. and Holmes,E.C.
EPI_ISL_2575507	J. Craig Venter Institute	J. Craig Venter Institute	Tan,G., Pickett,B., Fedorova,N., Amedeo,P., Hu,L., Christensen,J., Miller,J., Durbin,A., Williams,T., Arumemi,F., Cadiz,C., Alanis,R., Balmseda,A., Wiliams,T., Schiller,A., Patel,M., Kubale,J. and Gordon,A.
EPI_ISL_2575508, EPI_ISL_2575509, EPI_ISL_2575510, EPI_ISL_2575511	Marie Bashir Institute for Infectious Diseases and Biosecurity & Sydney Medical School, The University of Sydney, Westmead Institute for Medical Research	Marie Bashir Institute for Infectious Diseases and Biosecurity & Sydney Medical School, The University of Sydney, Westmead Institute for Medical Research	Eden,J.-S., Kok,J., Dwyer,D.E., Fernandez,M., Carter,I. and Holmes,E.C.
EPI_ISL_2575512	Michiko Okamoto Graduate School of Medicine, Tohoku University, Virology	Michiko Okamoto Graduate School of Medicine, Tohoku University, Virology	Okamoto,M. and Oshitani,H.
EPI_ISL_2575513	Marie Bashir Institute for Infectious Diseases and Biosecurity & Sydney Medical School, The University of Sydney, Westmead Institute for Medical Research	Marie Bashir Institute for Infectious Diseases and Biosecurity & Sydney Medical School, The University of Sydney, Westmead Institute for Medical Research	Eden,J.-S., Kok,J., Dwyer,D.E., Fernandez,M., Carter,I. and Holmes,E.C.
EPI_ISL_2575515, EPI_ISL_2575516, EPI_ISL_2575517	Reference Microbiology, Public Health England National Infection Services	Reference Microbiology, Public Health England National Infection Services	Valappil,M., Talts,T., Ellis,J., Sails,A., Eltringham,G., Waugh,S., Gould,K., Harrison,I., Pebody,R. and Zambon,M.
EPI_ISL_2575518, EPI_ISL_2575522, EPI_ISL_2575523, EPI_ISL_2575524	Pediatrics, University of New Mexico	Pediatrics, University of New Mexico	Dinwiddle,D.
EPI_ISL_2575531	Center for Infectious Diseases, School of Public Health, University of Texas Health Science Center	Center for Infectious Diseases, School of Public Health, University of Texas Health Science Center	Bahl,J., Hixson,J., Kim,D.-K., Qiu,X., Piedra,P.A., Piedra,F.-A., Avadhanula,V. and Machado,A.A.
EPI_ISL_2575532	Epidemiology and Demography Department, KEMRI Wellcome Trust Research Collaborative Programme	Epidemiology and Demography Department, KEMRI Wellcome Trust Research Collaborative Programme	Agoti,C.N., Phan,M.V.T., Munywoki,P.K., Githinji,G., Medley,G.F., Cane,P.A., Kellam,K., Cotten,M. and Nokes,D.J.
EPI_ISL_2575536, EPI_ISL_2575538	Marie Bashir Institute for Infectious Diseases and Biosecurity & Sydney Medical School, The University of Sydney, Westmead Institute for Medical Research	Marie Bashir Institute for Infectious Diseases and Biosecurity & Sydney Medical School, The University of Sydney, Westmead Institute for Medical Research	Eden,J.-S., Kok,J., Dwyer,D.E., Fernandez,M., Carter,I. and Holmes,E.C.
EPI_ISL_2575546, EPI_ISL_2575547	Department of Experimental Modeling and Infectious Diseases Pathogenesis, Federal Research Center of Fundamental and Translational Medicine	Department of Experimental Modeling and Infectious Diseases Pathogenesis, Federal Research Center of Fundamental and Translational Medicine	Kurskaya,O.G., Sobolev,I.A., Sharshov,K.A., Alexeev,A.Y., Murashkina,T.A., Kabilov,M.R., Alikina,T.Y. and Shestopalov,A.M.
EPI_ISL_2575548	J. Craig Venter Institute	J. Craig Venter Institute	Tan,G., Pickett,B., Fedorova,N., Amedeo,P., Hu,L., Christensen,J., Miller,J., Durbin,A., Williams,T., Arumemi,F., Cadiz,C., Alanis,R., Balmseda,A., Wiliams,T., Schiller,A., Patel,M., Kubale,J. and Gordon,A.
EPI_ISL_2575549	Marie Bashir Institute for Infectious Diseases and Biosecurity & Sydney Medical School, The University of Sydney, Westmead Institute for Medical Research	Marie Bashir Institute for Infectious Diseases and Biosecurity & Sydney Medical School, The University of Sydney, Westmead Institute for Medical Research	Eden,J.-S., Kok,J., Dwyer,D.E., Fernandez,M., Carter,I. and Holmes,E.C.
EPI_ISL_2575564	Reference Microbiology, Public Health England National Infection Services	Reference Microbiology, Public Health England National Infection Services	Valappil,M., Talts,T., Ellis,J., Sails,A., Eltringham,G., Waugh,S., Gould,K., Harrison,I., Pebody,R. and Zambon,M.
EPI_ISL_2575565	Epidemiology and Demography Department, KEMRI Wellcome Trust Research Collaborative Programme	Epidemiology and Demography Department, KEMRI Wellcome Trust Research Collaborative Programme	Agoti,C.N., Phan,M.V.T., Munywoki,P.K., Githinji,G., Medley,G.F., Cane,P.A., Kellam,K., Cotten,M. and Nokes,D.J.
EPI_ISL_2575566	J. Craig Venter Institute	J. Craig Venter Institute	Tan,G., Pickett,B., Fedorova,N., Amedeo,P., Isom,R., Hu,L., Christensen,J., Miller,J., Novotny,M., Durbin,A., Rocchi,I., Williams,T., Arumemi,F. and Das,S.
EPI_ISL_2575567	J. Craig Venter Institute	J. Craig Venter Institute	Tan,G., Pickett,B., Fedorova,N., Amedeo,P., Hu,L., Christensen,J., Miller,J., Durbin,A., Williams,T., Arumemi,F., Cadiz,C., Alanis,R., Balmseda,A., Wiliams,T., Schiller,A., Patel,M., Kubale,J. and Gordon,A.
EPI_ISL_2575568, EPI_ISL_2575569	Epidemiology and Demography Department, KEMRI Wellcome Trust Research Collaborative Programme	Epidemiology and Demography Department, KEMRI Wellcome Trust Research Collaborative Programme	Agoti,C.N., Phan,M.V.T., Munywoki,P.K., Githinji,G., Medley,G.F., Cane,P.A., Kellam,K., Cotten,M. and Nokes,D.J.
EPI_ISL_2575570	J. Craig Venter Institute	J. Craig Venter Institute	Tan,G., Pickett,B., Fedorova,N., Amedeo,P., Hu,L., Christensen,J., Miller,J., Durbin,A., Williams,T., Arumemi,F., Cadiz,C., Alanis,R., Balmseda,A., Wiliams,T., Schiller,A., Patel,M., Kubale,J. and Gordon,A.
EPI_ISL_2575571	Department of Experimental Modeling and Pathogenesis of Infectious Diseases, Federal Research Center of Fundamental and Translational Medicine	Department of Experimental Modeling and Pathogenesis of Infectious Diseases, Federal Research Center of Fundamental and Translational Medicine	Dubovitskiy,N.A., Sobolev,I.A., Kurskaya,O.G., Sharshov,K.A., Anoshina,A.V., Leonova,N.V., Murashkina,T.A., Solomatina,M.V., Derko,A.A., Saroyan,T.A., Kabilov,M.R., Alikina,T.Y. and Shestopalov,A.M.
EPI_ISL_2575572, EPI_ISL_2575573, EPI_ISL_2575574, EPI_ISL_2575575, EPI_ISL_2575576, EPI_ISL_2575577, EPI_ISL_2575578, EPI_ISL_2575579	Epidemiology and Demography Department, KEMRI Wellcome Trust Research Collaborative Programme	Epidemiology and Demography Department, KEMRI Wellcome Trust Research Collaborative Programme	Agoti,C.N., Phan,M.V.T., Munywoki,P.K., Githinji,G., Medley,G.F., Cane,P.A., Kellam,K., Cotten,M. and Nokes,D.J.
EPI_ISL_2575580, EPI_ISL_2575581, EPI_ISL_2575582, EPI_ISL_2575583, EPI_ISL_2575584, EPI_ISL_2575585, EPI_ISL_2575586, EPI_ISL_2575587	J. Craig Venter Institute	J. Craig Venter Institute	Tan,G., Pickett,B., Fedorova,N., Amedeo,P., Hu,L., Christensen,J., Miller,J., Durbin,A., Williams,T., Arumemi,F., Cadiz,C., Alanis,R., Balmseda,A., Wiliams,T., Schiller,A., Patel,M., Kubale,J. and Gordon,A.

EPI_ISL_2575588, EPI_ISL_2575589, EPI_ISL_2575590	Marie Bashir Institute for Infectious Diseases and Biosecurity & Sydney Medical School, The University of Sydney, Westmead Institute for Medical Research	Marie Bashir Institute for Infectious Diseases and Biosecurity & Sydney Medical School, The University of Sydney, Westmead Institute for Medical Research	Eden,J.-S., Kok,J., Dwyer,D.E., Fernandez,M., Carter,I. and Holmes,E.C.
EPI_ISL_2575591	Epidemiology and Demography Department, KEMRI Wellcome Trust Research Collaborative Programme	Epidemiology and Demography Department, KEMRI Wellcome Trust Research Collaborative Programme	Agoti,C.N., Phan,M.V.T., Munywoki,P.K., Githinji,G., Medley,G.F., Cane,P.A., Kellam,K., Cotten,M. and Nokes,D.J.
EPI_ISL_2575592	Reference Microbiology, Public Health England National Infection Services	Reference Microbiology, Public Health England National Infection Services	Valappil,M., Talts,T., Ellis,J., Sails,A., Eltringham,G., Waugh,S., Gould,K., Harrison,I., Pebody,R. and Zambon,M.
EPI_ISL_2575593	Kazuya Shirato National Institute of Infectious Diseases, Virology III	Kazuya Shirato National Institute of Infectious Diseases, Virology III	Shirato,K., Sato,K., Dapat,I., Nao,N., Omiya,S., Matsuyama,S., Takeda,M. and Nishimura,H.
EPI_ISL_2575594, EPI_ISL_2575595, EPI_ISL_2575596	J. Craig Venter Institute	J. Craig Venter Institute	Tan,G., Pickett,B., Fedorova,N., Amedeo,P., Hu,L., Christensen,J., Miller,J., Durbin,A., Williams,T., Arumemi,F., Cadiz,C., Alanis,R., Balmseda,A., Wiliams,T., Schiller,A., Patel,M., Kubale,J. and Gordon,A.
EPI_ISL_2575597, EPI_ISL_2575598, EPI_ISL_2575599, EPI_ISL_2575600	Marie Bashir Institute for Infectious Diseases and Biosecurity & Sydney Medical School, The University of Sydney, Westmead Institute for Medical Research	Marie Bashir Institute for Infectious Diseases and Biosecurity & Sydney Medical School, The University of Sydney, Westmead Institute for Medical Research	Eden,J.-S., Kok,J., Dwyer,D.E., Fernandez,M., Carter,I. and Holmes,E.C.
EPI_ISL_2575601	J. Craig Venter Institute	J. Craig Venter Institute	Tan,G., Pickett,B., Fedorova,N., Amedeo,P., Isom,R., Hu,L., Christensen,J., Miller,J., Novotny,M., Durbin,A., Rocchi,I., Williams,T., Arumemi,F. and Das,S.
EPI_ISL_2575602, EPI_ISL_2575603, EPI_ISL_2575604, EPI_ISL_2575605	Marie Bashir Institute for Infectious Diseases and Biosecurity & Sydney Medical School, The University of Sydney, Westmead Institute for Medical Research	Marie Bashir Institute for Infectious Diseases and Biosecurity & Sydney Medical School, The University of Sydney, Westmead Institute for Medical Research	Eden,J.-S., Kok,J., Dwyer,D.E., Fernandez,M., Carter,I. and Holmes,E.C.
EPI_ISL_2575606	Department of Experimental Modeling and Pathogenesis of Infectious Diseases, Federal Research Center of Fundamental and Translational Medicine	Department of Experimental Modeling and Pathogenesis of Infectious Diseases, Federal Research Center of Fundamental and Translational Medicine	Dubovitskiy,N.A., Sobolev,I.A., Kurskaya,O.G., Sharshov,K.A., Anoshina,A.V., Leonova,N.V., Murashkina,T.A., Solomatina,M.V., Derko,A.A., Saroyan,T.A., Kabilov,M.R., Alikina,T.Y. and Shestopalov,A.M.
EPI_ISL_2575607, EPI_ISL_2575608, EPI_ISL_2575609, EPI_ISL_2575610, EPI_ISL_2575611, EPI_ISL_2575612, EPI_ISL_2575613, EPI_ISL_2575614	Marie Bashir Institute for Infectious Diseases and Biosecurity & Sydney Medical School, The University of Sydney, Westmead Institute for Medical Research	Marie Bashir Institute for Infectious Diseases and Biosecurity & Sydney Medical School, The University of Sydney, Westmead Institute for Medical Research	Eden,J.-S., Kok,J., Dwyer,D.E., Fernandez,M., Carter,I. and Holmes,E.C.
EPI_ISL_2575615	Department of Experimental Modeling and Pathogenesis of Infectious Diseases, Federal Research Center of Fundamental and Translational Medicine	Department of Experimental Modeling and Pathogenesis of Infectious Diseases, Federal Research Center of Fundamental and Translational Medicine	Dubovitskiy,N.A., Sobolev,I.A., Kurskaya,O.G., Sharshov,K.A., Anoshina,A.V., Leonova,N.V., Murashkina,T.A., Solomatina,M.V., Derko,A.A., Saroyan,T.A., Kabilov,M.R., Alikina,T.Y. and Shestopalov,A.M.
EPI_ISL_2575617	J. Craig Venter Institute	J. Craig Venter Institute	Tan,G., Pickett,B., Fedorova,N., Amedeo,P., Isom,R., Hu,L., Christensen,J., Miller,J., Novotny,M., Durbin,A., Rocchi,I., Williams,T., Arumemi,F. and Das,S.
EPI_ISL_2575618, EPI_ISL_2575619, EPI_ISL_2575620	J. Craig Venter Institute	J. Craig Venter Institute	Tan,G., Pickett,B., Fedorova,N., Amedeo,P., Hu,L., Christensen,J., Miller,J., Durbin,A., Williams,T., Arumemi,F., Cadiz,C., Alanis,R., Balmseda,A., Wiliams,T., Schiller,A., Patel,M., Kubale,J. and Gordon,A.
EPI_ISL_2575621, EPI_ISL_2575623, EPI_ISL_2577154, EPI_ISL_2577155	Marie Bashir Institute for Infectious Diseases and Biosecurity & Sydney Medical School, The University of Sydney, Westmead Institute for Medical Research	Marie Bashir Institute for Infectious Diseases and Biosecurity & Sydney Medical School, The University of Sydney, Westmead Institute for Medical Research	Eden,J.-S., Kok,J., Dwyer,D.E., Fernandez,M., Carter,I. and Holmes,E.C.
EPI_ISL_2577156	Reference Microbiology, Public Health England National Infection Services	Reference Microbiology, Public Health England National Infection Services	Valappil,M., Talts,T., Ellis,J., Sails,A., Eltringham,G., Waugh,S., Gould,K., Harrison,I., Pebody,R. and Zambon,M.
EPI_ISL_2577266, EPI_ISL_2577267, EPI_ISL_2577268, EPI_ISL_2577269, EPI_ISL_2577278, EPI_ISL_2577279, EPI_ISL_2577280, EPI_ISL_2577281, EPI_ISL_2577282, EPI_ISL_2577283, EPI_ISL_2577284			
see above	Epidemiology and Demography Department, KEMRI Wellcome Trust Research Collaborative Programme	Epidemiology and Demography Department, KEMRI Wellcome Trust Research Collaborative Programme	Agoti,C.N., Phan,M.V.T., Munywoki,P.K., Githinji,G., Medley,G.F., Cane,P.A., Kellam,K., Cotten,M. and Nokes,D.J.
EPI_ISL_2577285	J. Craig Venter Institute	J. Craig Venter Institute	Tan,G., Pickett,B., Fedorova,N., Amedeo,P., Isom,R., Hu,L., Christensen,J., Miller,J., Novotny,M., Durbin,A., Rocchi,I., Williams,T., Arumemi,F. and Das,S.
EPI_ISL_2577286, EPI_ISL_2577287, EPI_ISL_2577288	Reference Microbiology, Public Health England National Infection Services	Reference Microbiology, Public Health England National Infection Services	Valappil,M., Talts,T., Ellis,J., Sails,A., Eltringham,G., Waugh,S., Gould,K., Harrison,I., Pebody,R. and Zambon,M.
EPI_ISL_2577289	J. Craig Venter Institute	J. Craig Venter Institute	Tan,G., Pickett,B., Fedorova,N., Amedeo,P., Isom,R., Hu,L., Christensen,J., Miller,J., Novotny,M., Durbin,A., Rocchi,I., Williams,T., Arumemi,F. and Das,S.
EPI_ISL_2577291	Marie Bashir Institute for Infectious Diseases and Biosecurity & Sydney Medical School, The University of Sydney, Westmead Institute for Medical Research	Marie Bashir Institute for Infectious Diseases and Biosecurity & Sydney Medical School, The University of Sydney, Westmead Institute for Medical Research	Eden,J.-S., Kok,J., Dwyer,D.E., Fernandez,M., Carter,I. and Holmes,E.C.
EPI_ISL_2577292, EPI_ISL_2577293, EPI_ISL_2577294, EPI_ISL_2577295, EPI_ISL_2577296, EPI_ISL_2577297, EPI_ISL_2577298, EPI_ISL_2577299	Epidemiology and Demography Department, KEMRI Wellcome Trust Research Collaborative Programme	Epidemiology and Demography Department, KEMRI Wellcome Trust Research Collaborative Programme	Agoti,C.N., Phan,M.V.T., Munywoki,P.K., Githinji,G., Medley,G.F., Cane,P.A., Kellam,K., Cotten,M. and Nokes,D.J.
EPI_ISL_2577300	Marie Bashir Institute for Infectious Diseases and Biosecurity & Sydney Medical School, The University of Sydney, Westmead Institute for Medical Research	Marie Bashir Institute for Infectious Diseases and Biosecurity & Sydney Medical School, The University of Sydney, Westmead Institute for Medical Research	Eden,J.-S., Kok,J., Dwyer,D.E., Fernandez,M., Carter,I. and Holmes,E.C.
EPI_ISL_2577301	Pediatrics - Infectious Diseases, Medical College of Wisconsin	Pediatrics - Infectious Diseases, Medical College of Wisconsin	Rebuffo-Scheer,C., Bose,M.E., He,J., Khaja,S., Ulatowski,M., Beck,E.T., Fan,J., Kumar,S., Nelson,M.I. and Henrickson,K.J.
EPI_ISL_2577302, EPI_ISL_2577303, EPI_ISL_2577304, EPI_ISL_2577305, EPI_ISL_2577306, EPI_ISL_2577307	Epidemiology and Demography Department, KEMRI Wellcome Trust Research Collaborative Programme	Epidemiology and Demography Department, KEMRI Wellcome Trust Research Collaborative Programme	Agoti,C.N., Phan,M.V.T., Munywoki,P.K., Githinji,G., Medley,G.F., Cane,P.A., Kellam,K., Cotten,M. and Nokes,D.J.
EPI_ISL_2577308	Reference Microbiology, Public Health England National Infection Services	Reference Microbiology, Public Health England National Infection Services	Valappil,M., Talts,T., Ellis,J., Sails,A., Eltringham,G., Waugh,S., Gould,K., Harrison,I., Pebody,R. and Zambon,M.
EPI_ISL_2577309, EPI_ISL_2577310	Michiko Okamoto Graduate School of Medicine, Tohoku University, Virology	Michiko Okamoto Graduate School of Medicine, Tohoku University, Virology	Okamoto,M. and Oshitani,H.
EPI_ISL_2577311, EPI_ISL_2577312	Marie Bashir Institute for Infectious Diseases and Biosecurity & Sydney Medical School, The University of Sydney, Westmead Institute for Medical Research	Marie Bashir Institute for Infectious Diseases and Biosecurity & Sydney Medical School, The University of Sydney, Westmead Institute for Medical Research	Eden,J.-S., Kok,J., Dwyer,D.E., Fernandez,M., Carter,I. and Holmes,E.C.
EPI_ISL_2577323	Epidemiology and Demography Department, KEMRI Wellcome Trust Research Collaborative Programme	Epidemiology and Demography Department, KEMRI Wellcome Trust Research Collaborative Programme	Agoti,C.N., Phan,M.V.T., Munywoki,P.K., Githinji,G., Medley,G.F., Cane,P.A., Kellam,K., Cotten,M. and Nokes,D.J.
EPI_ISL_2577324, EPI_ISL_2577325	Marie Bashir Institute for Infectious Diseases and Biosecurity & Sydney Medical School, The University of Sydney, Westmead Institute for Medical Research	Marie Bashir Institute for Infectious Diseases and Biosecurity & Sydney Medical School, The University of Sydney, Westmead Institute for Medical Research	Eden,J.-S., Kok,J., Dwyer,D.E., Fernandez,M., Carter,I. and Holmes,E.C.
EPI_ISL_2577326	Reference Microbiology, Public Health England National Infection Services	Reference Microbiology, Public Health England National Infection Services	Valappil,M., Talts,T., Ellis,J., Sails,A., Eltringham,G., Waugh,S., Gould,K., Harrison,I., Pebody,R. and Zambon,M.
EPI_ISL_2577327, EPI_ISL_2577328,	Marie Bashir Institute for Infectious Diseases and Biosecurity	Marie Bashir Institute for Infectious Diseases and Biosecurity	Eden,J.-S., Kok,J., Dwyer,D.E., Fernandez,M., Carter,I. and Holmes,E.C.

EPI_ISL_2577329, EPI_ISL_2577330	& Sydney Medical School, The University of Sydney, Westmead Institute for Medical Research	& Sydney Medical School, The University of Sydney, Westmead Institute for Medical Research	
EPI_ISL_2577331	Michiko Okamoto Graduate School of Medicine, Tohoku University, Virology	Michiko Okamoto Graduate School of Medicine, Tohoku University, Virology	Okamoto,M. and Oshitani,H.
EPI_ISL_2577334	J. Craig Venter Institute	J. Craig Venter Institute	Tan,G., Pickett,B., Fedorova,N., Amedeo,P., Isom,R., Hu,L., Christensen,J., Miller,J., Novotny,M., Durbin,A., Rocchi,I., Williams,T., Arumemi,F. and Das,S.
EPI_ISL_2577335, EPI_ISL_2577336, EPI_ISL_2577337, EPI_ISL_2577338, EPI_ISL_2577339, EPI_ISL_2577382	Marie Bashir Institute for Infectious Diseases and Biosecurity & Sydney Medical School, The University of Sydney, Westmead Institute for Medical Research	Marie Bashir Institute for Infectious Diseases and Biosecurity & Sydney Medical School, The University of Sydney, Westmead Institute for Medical Research	Eden,J.-S., Kok,J., Dwyer,D.E., Fernandez,M., Carter,I. and Holmes,E.C.
EPI_ISL_2577383	J. Craig Venter Institute	J. Craig Venter Institute	Tan,G., Pickett,B., Fedorova,N., Amedeo,P., Isom,R., Hu,L., Christensen,J., Miller,J., Novotny,M., Durbin,A., Rocchi,I., Williams,T., Arumemi,F. and Das,S.
EPI_ISL_2577384	Michiko Okamoto Graduate School of Medicine, Tohoku University, Virology	Michiko Okamoto Graduate School of Medicine, Tohoku University, Virology	Okamoto,M. and Oshitani,H.
EPI_ISL_2577385	Reference Microbiology, Public Health England National Infection Services	Reference Microbiology, Public Health England National Infection Services	Valappil,M., Talts,T., Ellis,J., Sails,A., Eltringham,G., Waugh,S., Gould,K., Harrison,I., Pebody,R. and Zambon,M.
EPI_ISL_2577386, EPI_ISL_2577387	Marie Bashir Institute for Infectious Diseases and Biosecurity & Sydney Medical School, The University of Sydney, Westmead Institute for Medical Research	Marie Bashir Institute for Infectious Diseases and Biosecurity & Sydney Medical School, The University of Sydney, Westmead Institute for Medical Research	Eden,J.-S., Kok,J., Dwyer,D.E., Fernandez,M., Carter,I. and Holmes,E.C.
EPI_ISL_2577388, EPI_ISL_2577389, EPI_ISL_2577390, EPI_ISL_2577391, EPI_ISL_2577392, EPI_ISL_2577393	Epidemiology and Demography Department, KEMRI Wellcome Trust Research Collaborative Programme	Epidemiology and Demography Department, KEMRI Wellcome Trust Research Collaborative Programme	Agoti,C.N., Phan,M.V.T., Munywoki,P.K., Githinji,G., Medley,G.F., Cane,P.A., Kellam,K., Cotten,M. and Nokes,D.J.
EPI_ISL_2577394, EPI_ISL_2577395, EPI_ISL_2577396	Reference Microbiology, Public Health England National Infection Services	Reference Microbiology, Public Health England National Infection Services	Valappil,M., Talts,T., Ellis,J., Sails,A., Eltringham,G., Waugh,S., Gould,K., Harrison,I., Pebody,R. and Zambon,M.
EPI_ISL_2577397, EPI_ISL_2577398, EPI_ISL_2577399	J. Craig Venter Institute	J. Craig Venter Institute	Tan,G., Pickett,B., Fedorova,N., Amedeo,P., Hu,L., Christensen,J., Miller,J., Durbin,A., Williams,T., Arumemi,F., Cadiz,C., Alanis,R., Balmseda,A., Wiliams,T., Schiller,A., Patel,M., Kubale,J. and Gordon,A.
EPI_ISL_2577400, EPI_ISL_2577401, EPI_ISL_2577402	Marie Bashir Institute for Infectious Diseases and Biosecurity & Sydney Medical School, The University of Sydney, Westmead Institute for Medical Research	Marie Bashir Institute for Infectious Diseases and Biosecurity & Sydney Medical School, The University of Sydney, Westmead Institute for Medical Research	Eden,J.-S., Kok,J., Dwyer,D.E., Fernandez,M., Carter,I. and Holmes,E.C.
EPI_ISL_2577403, EPI_ISL_2577404, EPI_ISL_2577405	Epidemiology and Demography Department, KEMRI Wellcome Trust Research Collaborative Programme	Epidemiology and Demography Department, KEMRI Wellcome Trust Research Collaborative Programme	Agoti,C.N., Phan,M.V.T., Munywoki,P.K., Githinji,G., Medley,G.F., Cane,P.A., Kellam,K., Cotten,M. and Nokes,D.J.
EPI_ISL_2577406	J. Craig Venter Institute	J. Craig Venter Institute	Tan,G., Pickett,B., Fedorova,N., Amedeo,P., Isom,R., Hu,L., Christensen,J., Miller,J., Novotny,M., Durbin,A., Rocchi,I., Williams,T., Arumemi,F. and Das,S.
EPI_ISL_2577407	Marie Bashir Institute for Infectious Diseases and Biosecurity & Sydney Medical School, The University of Sydney, Westmead Institute for Medical Research	Marie Bashir Institute for Infectious Diseases and Biosecurity & Sydney Medical School, The University of Sydney, Westmead Institute for Medical Research	Eden,J.-S., Kok,J., Dwyer,D.E., Fernandez,M., Carter,I. and Holmes,E.C.
EPI_ISL_2577408	Reference Microbiology, Public Health England National Infection Services	Reference Microbiology, Public Health England National Infection Services	Valappil,M., Talts,T., Ellis,J., Sails,A., Eltringham,G., Waugh,S., Gould,K., Harrison,I., Pebody,R. and Zambon,M.
EPI_ISL_2577409, EPI_ISL_2577410	Marie Bashir Institute for Infectious Diseases and Biosecurity & Sydney Medical School, The University of Sydney, Westmead Institute for Medical Research	Marie Bashir Institute for Infectious Diseases and Biosecurity & Sydney Medical School, The University of Sydney, Westmead Institute for Medical Research	Eden,J.-S., Kok,J., Dwyer,D.E., Fernandez,M., Carter,I. and Holmes,E.C.
EPI_ISL_2577411, EPI_ISL_2577412, EPI_ISL_2577413	J. Craig Venter Institute	J. Craig Venter Institute	Tan,G., Pickett,B., Fedorova,N., Amedeo,P., Hu,L., Christensen,J., Miller,J., Durbin,A., Williams,T., Arumemi,F., Cadiz,C., Alanis,R., Balmseda,A., Wiliams,T., Schiller,A., Patel,M., Kubale,J. and Gordon,A.
EPI_ISL_2577414, EPI_ISL_2577415	Marie Bashir Institute for Infectious Diseases and Biosecurity & Sydney Medical School, The University of Sydney, Westmead Institute for Medical Research	Marie Bashir Institute for Infectious Diseases and Biosecurity & Sydney Medical School, The University of Sydney, Westmead Institute for Medical Research	Eden,J.-S., Kok,J., Dwyer,D.E., Fernandez,M., Carter,I. and Holmes,E.C.
EPI_ISL_2577416, EPI_ISL_2577417, EPI_ISL_2577418, EPI_ISL_2577419	Epidemiology and Demography Department, KEMRI Wellcome Trust Research Collaborative Programme	Epidemiology and Demography Department, KEMRI Wellcome Trust Research Collaborative Programme	Agoti,C.N., Phan,M.V.T., Munywoki,P.K., Githinji,G., Medley,G.F., Cane,P.A., Kellam,K., Cotten,M. and Nokes,D.J.
EPI_ISL_2577420	Marie Bashir Institute for Infectious Diseases and Biosecurity & Sydney Medical School, The University of Sydney, Westmead Institute for Medical Research	Marie Bashir Institute for Infectious Diseases and Biosecurity & Sydney Medical School, The University of Sydney, Westmead Institute for Medical Research	Eden,J.-S., Kok,J., Dwyer,D.E., Fernandez,M., Carter,I. and Holmes,E.C.
EPI_ISL_2577421, EPI_ISL_2577422	Reference Microbiology, Public Health England National Infection Services	Reference Microbiology, Public Health England National Infection Services	Valappil,M., Talts,T., Ellis,J., Sails,A., Eltringham,G., Waugh,S., Gould,K., Harrison,I., Pebody,R. and Zambon,M.
EPI_ISL_2577423, EPI_ISL_2577424	Marie Bashir Institute for Infectious Diseases and Biosecurity & Sydney Medical School, The University of Sydney, Westmead Institute for Medical Research	Marie Bashir Institute for Infectious Diseases and Biosecurity & Sydney Medical School, The University of Sydney, Westmead Institute for Medical Research	Eden,J.-S., Kok,J., Dwyer,D.E., Fernandez,M., Carter,I. and Holmes,E.C.
EPI_ISL_2577425	J. Craig Venter Institute	J. Craig Venter Institute	Tan,G., Pickett,B., Fedorova,N., Amedeo,P., Hu,L., Christensen,J., Miller,J., Durbin,A., Williams,T., Arumemi,F., Cadiz,C., Alanis,R., Balmseda,A., Wiliams,T., Schiller,A., Patel,M., Kubale,J. and Gordon,A.
EPI_ISL_2577426	Reference Microbiology, Public Health England National Infection Services	Reference Microbiology, Public Health England National Infection Services	Valappil,M., Talts,T., Ellis,J., Sails,A., Eltringham,G., Waugh,S., Gould,K., Harrison,I., Pebody,R. and Zambon,M.
EPI_ISL_2577427, EPI_ISL_2577428	Marie Bashir Institute for Infectious Diseases and Biosecurity & Sydney Medical School, The University of Sydney, Westmead Institute for Medical Research	Marie Bashir Institute for Infectious Diseases and Biosecurity & Sydney Medical School, The University of Sydney, Westmead Institute for Medical Research	Eden,J.-S., Kok,J., Dwyer,D.E., Fernandez,M., Carter,I. and Holmes,E.C.
EPI_ISL_2577429	J. Craig Venter Institute	J. Craig Venter Institute	Tan,G., Pickett,B., Fedorova,N., Amedeo,P., Hu,L., Christensen,J., Miller,J., Durbin,A., Williams,T., Arumemi,F., Cadiz,C., Alanis,R., Balmseda,A., Wiliams,T., Schiller,A., Patel,M., Kubale,J. and Gordon,A.
EPI_ISL_2577430	Marie Bashir Institute for Infectious Diseases and Biosecurity & Sydney Medical School, The University of Sydney, Westmead Institute for Medical Research	Marie Bashir Institute for Infectious Diseases and Biosecurity & Sydney Medical School, The University of Sydney, Westmead Institute for Medical Research	Eden,J.-S., Kok,J., Dwyer,D.E., Fernandez,M., Carter,I. and Holmes,E.C.
EPI_ISL_2577431, EPI_ISL_2577432, EPI_ISL_2577433, EPI_ISL_2577434	Epidemiology and Demography Department, KEMRI Wellcome Trust Research Collaborative Programme	Epidemiology and Demography Department, KEMRI Wellcome Trust Research Collaborative Programme	Agoti,C.N., Phan,M.V.T., Munywoki,P.K., Githinji,G., Medley,G.F., Cane,P.A., Kellam,K., Cotten,M. and Nokes,D.J.
EPI_ISL_2577435	Marie Bashir Institute for Infectious Diseases and Biosecurity & Sydney Medical School, The University of Sydney, Westmead Institute for Medical Research	Marie Bashir Institute for Infectious Diseases and Biosecurity & Sydney Medical School, The University of Sydney, Westmead Institute for Medical Research	Eden,J.-S., Kok,J., Dwyer,D.E., Fernandez,M., Carter,I. and Holmes,E.C.
EPI_ISL_2577436, EPI_ISL_2577437	J. Craig Venter Institute	J. Craig Venter Institute	Tan,G., Pickett,B., Fedorova,N., Amedeo,P., Isom,R., Hu,L., Christensen,J., Miller,J., Novotny,M., Durbin,A., Rocchi,I., Williams,T., Arumemi,F. and Das,S.
EPI_ISL_2577438, EPI_ISL_2577439, EPI_ISL_2577440	Marie Bashir Institute for Infectious Diseases and Biosecurity & Sydney Medical School, The University of Sydney,	Marie Bashir Institute for Infectious Diseases and Biosecurity & Sydney Medical School, The University of Sydney,	Eden,J.-S., Kok,J., Dwyer,D.E., Fernandez,M., Carter,I. and Holmes,E.C.

	Westmead Institute for Medical Research	Westmead Institute for Medical Research	
EPI_ISL_2577441, EPI_ISL_2577442, EPI_ISL_2577443	J. Craig Venter Institute	J. Craig Venter Institute	Tan,G., Pickett,B., Fedorova,N., Amedeo,P., Hu,L., Christensen,J., Miller,J., Durbin,A., Williams,T., Arumemi,F., Cadiz,C., Alanis,R., Balmseda,A., Wiliams,T., Schiller,A., Patel,M., Kubale,J. and Gordon,A.
EPI_ISL_2577444	Marie Bashir Institute for Infectious Diseases and Biosecurity & Sydney Medical School, The University of Sydney, Westmead Institute for Medical Research	Marie Bashir Institute for Infectious Diseases and Biosecurity & Sydney Medical School, The University of Sydney, Westmead Institute for Medical Research	Eden,J.-S., Kok,J., Dwyer,D.E., Fernandez,M., Carter,I. and Holmes,E.C.
EPI_ISL_2577471, EPI_ISL_2577472, EPI_ISL_2577473, EPI_ISL_2577474, EPI_ISL_2577475, EPI_ISL_2577476, EPI_ISL_2577477, EPI_ISL_2577478, EPI_ISL_2577479, EPI_ISL_2577480	Epidemiology and Demography Department, KEMRI Wellcome Trust Research Collaborative Programme	Epidemiology and Demography Department, KEMRI Wellcome Trust Research Collaborative Programme	Agoti,C.N., Phan,M.V.T., Munywoki,P.K., Githinji,G., Medley,G.F., Cane,P.A., Kellam,K., Cotten,M. and Nokes,D.J.
EPI_ISL_2577483, EPI_ISL_2577484	Marie Bashir Institute for Infectious Diseases and Biosecurity & Sydney Medical School, The University of Sydney, Westmead Institute for Medical Research	Marie Bashir Institute for Infectious Diseases and Biosecurity & Sydney Medical School, The University of Sydney, Westmead Institute for Medical Research	Eden,J.-S., Kok,J., Dwyer,D.E., Fernandez,M., Carter,I. and Holmes,E.C.
EPI_ISL_2577662	Reference Microbiology, Public Health England National Infection Services	Reference Microbiology, Public Health England National Infection Services	Valappil,M., Talts,T., Ellis,J., Sails,A., Eltringham,G., Waugh,S., Gould,K., Harrison,I., Pebody,R. and Zambon,M.
EPI_ISL_2577698, EPI_ISL_2577709, EPI_ISL_2577719, EPI_ISL_2577730, EPI_ISL_2577736, EPI_ISL_2577749, EPI_ISL_2577754	J. Craig Venter Institute	J. Craig Venter Institute	Tan,G., Pickett,B., Fedorova,N., Amedeo,P., Isom,R., Hu,L., Christensen,J., Miller,J., Novotny,M., Durbin,A., Rocchi,I., Williams,T., Arumemi,F. and Das,S.
EPI_ISL_2577788	Central Laboratory, Guangzhou Women and Children's Medical Center	Central Laboratory, Guangzhou Women and Children's Medical Center	Xie,J.H., Zhu,B., Zhong,J.Y., Chen,Y. and Zhang,Y.Y.
EPI_ISL_2578154, EPI_ISL_2578157	Michiko Okamoto Graduate School of Medicine, Tohoku University, Virology	Michiko Okamoto Graduate School of Medicine, Tohoku University, Virology	Okamoto,M. and Oshitani,H.
EPI_ISL_2578161	Marie Bashir Institute for Infectious Diseases and Biosecurity & Sydney Medical School, The University of Sydney, Westmead Institute for Medical Research	Marie Bashir Institute for Infectious Diseases and Biosecurity & Sydney Medical School, The University of Sydney, Westmead Institute for Medical Research	Eden,J.-S., Kok,J., Dwyer,D.E., Fernandez,M., Carter,I. and Holmes,E.C.
EPI_ISL_2578167	Michiko Okamoto Graduate School of Medicine, Tohoku University, Virology	Michiko Okamoto Graduate School of Medicine, Tohoku University, Virology	Okamoto,M. and Oshitani,H.
EPI_ISL_2578239	Marie Bashir Institute for Infectious Diseases and Biosecurity & Sydney Medical School, The University of Sydney, Westmead Institute for Medical Research	Marie Bashir Institute for Infectious Diseases and Biosecurity & Sydney Medical School, The University of Sydney, Westmead Institute for Medical Research	Eden,J.-S., Kok,J., Dwyer,D.E., Fernandez,M., Carter,I. and Holmes,E.C.
EPI_ISL_2578352, EPI_ISL_2578361, EPI_ISL_2578362, EPI_ISL_2578365	J. Craig Venter Institute	J. Craig Venter Institute	Tan,G., Pickett,B., Fedorova,N., Amedeo,P., Hu,L., Christensen,J., Miller,J., Durbin,A., Williams,T., Arumemi,F., Cadiz,C., Alanis,R., Balmseda,A., Wiliams,T., Schiller,A., Patel,M., Kubale,J. and Gordon,A.
EPI_ISL_2578381	Marie Bashir Institute for Infectious Diseases and Biosecurity & Sydney Medical School, The University of Sydney, Westmead Institute for Medical Research	Marie Bashir Institute for Infectious Diseases and Biosecurity & Sydney Medical School, The University of Sydney, Westmead Institute for Medical Research	Eden,J.-S., Kok,J., Dwyer,D.E., Fernandez,M., Carter,I. and Holmes,E.C.
EPI_ISL_2578661	Pediatrics - Infectious Diseases, Medical College of Wisconsin	Pediatrics - Infectious Diseases, Medical College of Wisconsin	Rebuffo-Scheer,C., Bose,M.E., He,J., Khaja,S., Ulatowski,M., Beck,E.T., Fan,J., Kumar,S., Nelson,M.I. and Henrickson,K.J.
EPI_ISL_2578662	Beijing Key Laboratory of Etiology of Viral Diseases in Children; Laboratory of Virology, Capital Institute of Pediatrics	Beijing Key Laboratory of Etiology of Viral Diseases in Children; Laboratory of Virology, Capital Institute of Pediatrics	Cui,G., Zhu,R., Deng,J., Zhao,L., Sun,Y., Wang,F. and Qian,Y.
EPI_ISL_2578663, EPI_ISL_2578664, EPI_ISL_2578665, EPI_ISL_2578666, EPI_ISL_2578667, EPI_ISL_2578668, EPI_ISL_2578669, EPI_ISL_2578670, EPI_ISL_2578671, EPI_ISL_2578672, EPI_ISL_2578673, EPI_ISL_2578674, EPI_ISL_2578675, EPI_ISL_2578676			
see above	Epidemiology and Demography Department, KEMRI-Wellcome Trust Research Programme	Epidemiology and Demography Department, KEMRI-Wellcome Trust Research Programme	Otieno,J.R., Kamau,E.M., Oketch,J.W., Ngoi,J.M., Agoti,C.N., Gichuki,A.M., Otieno,G.P., Ngama,M., Cane,P.A., Kellam,P., Cotten,M., Lemey,P. and Nokes,D.J.
EPI_ISL_2578677, EPI_ISL_2578678	Center for Infectious Diseases, School of Public Health, University of Texas Health Science Center	Center for Infectious Diseases, School of Public Health, University of Texas Health Science Center	Bahl,J., Hixson,J., Kim,D.-K., Qiu,X., Piedra,P.A., Piedra,F.-A., Avadhanula,V. and Machado,A.A.
EPI_ISL_2578679	Marie Bashir Institute for Infectious Diseases and Biosecurity & Sydney Medical School, The University of Sydney, Westmead Institute for Medical Research	Marie Bashir Institute for Infectious Diseases and Biosecurity & Sydney Medical School, The University of Sydney, Westmead Institute for Medical Research	Eden,J.-S., Kok,J., Dwyer,D.E., Fernandez,M., Carter,I. and Holmes,E.C.
EPI_ISL_2578680, EPI_ISL_2578681, EPI_ISL_2578682, EPI_ISL_2578683, EPI_ISL_2578684, EPI_ISL_2578685, EPI_ISL_2578686, EPI_ISL_2578687, EPI_ISL_2578688, EPI_ISL_2578689, EPI_ISL_2578690			
see above	Epidemiology and Demography Department, KEMRI-Wellcome Trust Research Programme	Epidemiology and Demography Department, KEMRI-Wellcome Trust Research Programme	Otieno,J.R., Kamau,E.M., Oketch,J.W., Ngoi,J.M., Agoti,C.N., Gichuki,A.M., Otieno,G.P., Ngama,M., Cane,P.A., Kellam,P., Cotten,M., Lemey,P. and Nokes,D.J.
EPI_ISL_2578691	Department of Pediatrics, Center of Excellence in Clinical Virology, Chulalongkorn	Department of Pediatrics, Center of Excellence in Clinical Virology, Chulalongkorn	Thongpan,I.
EPI_ISL_2578692	Center for Infectious Diseases, School of Public Health, University of Texas Health Science Center	Center for Infectious Diseases, School of Public Health, University of Texas Health Science Center	Bahl,J., Hixson,J., Kim,D.-K., Qiu,X., Piedra,P.A., Piedra,F.-A., Avadhanula,V. and Machado,A.A.
EPI_ISL_2578693, EPI_ISL_2578694, EPI_ISL_2578695, EPI_ISL_2578696, EPI_ISL_2578697, EPI_ISL_2578698, EPI_ISL_2578699, EPI_ISL_2578700, EPI_ISL_2578701, EPI_ISL_2578702, EPI_ISL_2578703, EPI_ISL_2578704, EPI_ISL_2578705, EPI_ISL_2578706, EPI_ISL_2578707, EPI_ISL_2578708, EPI_ISL_2578709, EPI_ISL_2578710, EPI_ISL_2578711			
see above	Epidemiology and Demography Department, KEMRI-Wellcome Trust Research Programme	Epidemiology and Demography Department, KEMRI-Wellcome Trust Research Programme	Otieno,J.R., Kamau,E.M., Oketch,J.W., Ngoi,J.M., Agoti,C.N., Gichuki,A.M., Otieno,G.P., Ngama,M., Cane,P.A., Kellam,P., Cotten,M., Lemey,P. and Nokes,D.J.
EPI_ISL_2578712	Pediatrics, University of New Mexico	Pediatrics, University of New Mexico	Kothari,A., Kennedy,J.L., Schwalm,K.C., Putt,C., Denson,J.L. and Dinwiddie,D.L.
EPI_ISL_2578713, EPI_ISL_2578714	Center for Infectious Diseases, School of Public Health, University of Texas Health Science Center	Center for Infectious Diseases, School of Public Health, University of Texas Health Science Center	Bahl,J., Hixson,J., Kim,D.-K., Qiu,X., Piedra,P.A., Piedra,F.-A., Avadhanula,V. and Machado,A.A.
EPI_ISL_2578715, EPI_ISL_2578716, EPI_ISL_2578717	Marie Bashir Institute for Infectious Diseases and Biosecurity & Sydney Medical School, The University of Sydney, Westmead Institute for Medical Research	Marie Bashir Institute for Infectious Diseases and Biosecurity & Sydney Medical School, The University of Sydney, Westmead Institute for Medical Research	Eden,J.-S., Kok,J., Dwyer,D.E., Fernandez,M., Carter,I. and Holmes,E.C.
EPI_ISL_2578718, EPI_ISL_2578719, EPI_ISL_2578720, EPI_ISL_2578721, EPI_ISL_2578722, EPI_ISL_2578723, EPI_ISL_2578724, EPI_ISL_2578725, EPI_ISL_2578726, EPI_ISL_2578727, EPI_ISL_2578728, EPI_ISL_2578729, EPI_ISL_2578730, EPI_ISL_2578731, EPI_ISL_2578732, EPI_ISL_2578733, EPI_ISL_2578734			
see above	Epidemiology and Demography Department, KEMRI-Wellcome Trust Research Programme	Epidemiology and Demography Department, KEMRI-Wellcome Trust Research Programme	Otieno,J.R., Kamau,E.M., Oketch,J.W., Ngoi,J.M., Agoti,C.N., Gichuki,A.M., Otieno,G.P., Ngama,M., Cane,P.A., Kellam,P., Cotten,M., Lemey,P. and Nokes,D.J.
EPI_ISL_2578767, EPI_ISL_2578768	Marie Bashir Institute for Infectious Diseases and Biosecurity & Sydney Medical School, The University of Sydney, Westmead Institute for Medical Research	Marie Bashir Institute for Infectious Diseases and Biosecurity & Sydney Medical School, The University of Sydney, Westmead Institute for Medical Research	Eden,J.-S., Kok,J., Dwyer,D.E., Fernandez,M., Carter,I. and Holmes,E.C.
EPI_ISL_2578769, EPI_ISL_2578770,	Epidemiology and Demography Department,	Epidemiology and Demography Department,	Otieno,J.R., Kamau,E.M., Oketch,J.W., Ngoi,J.M., Agoti,C.N., Gichuki,A.M., Otieno,G.P., Ngama,M., Cane,P.A., Kellam,P., Cotten,M., Lemey,P. and

EPI_ISL_2578771, EPI_ISL_2578772, EPI_ISL_2578773, EPI_ISL_2578774, EPI_ISL_2578775, EPI_ISL_2578776	KEMRI-Wellcome Trust Research Programme	KEMRI-Wellcome Trust Research Programme	Nokes,D.J.
EPI_ISL_2578777, EPI_ISL_2578778	J. Craig Venter Institute	J. Craig Venter Institute	Tan,G., Pickett,B., Fedorova,N., Amedeo,P., Hu,L., Christensen,J., Miller,J., Durbin,A., Williams,T., Arumemi,F., Cadiz,C., Alanis,R., Balmseda,A., Wiliams,T., Schiller,A., Patel,M., Kubale,J. and Gordon,A.
EPI_ISL_2578779, EPI_ISL_2578780	Virology, Graduate School of Medicine, Tohoku University	Virology, Graduate School of Medicine, Tohoku University	Malasao,R., Furuse,Y., Okamoto,M., Dapat,C., Saito,M., Saito-Obata,M., Tamaki,R., Segubre-Mercado,E., Lupisan,S. and Oshitani,H.
EPI_ISL_2578787	Department of Pediatrics, Center of Excellence in Clinical Virology, Chulalongkorn	Department of Pediatrics, Center of Excellence in Clinical Virology, Chulalongkorn	Thongpan,I.
EPI_ISL_2578788, EPI_ISL_2578789	Marie Bashir Institute for Infectious Diseases and Biosecurity & Sydney Medical School, The University of Sydney, Westmead Institute for Medical Research	Marie Bashir Institute for Infectious Diseases and Biosecurity & Sydney Medical School, The University of Sydney, Westmead Institute for Medical Research	Eden,J.-S., Kok,J., Dwyer,D.E., Fernandez,M., Carter,I. and Holmes,E.C.
EPI_ISL_2578799, EPI_ISL_2578800, EPI_ISL_2578801	Epidemiology and Demography Department, KEMRI-Wellcome Trust Research Programme	Epidemiology and Demography Department, KEMRI-Wellcome Trust Research Programme	Otieno,J.R., Kamau,E.M., Oketch,J.W., Ngoi,J.M., Agoti,C.N., Gichuki,A.M., Otieno,G.P., Ngama,M., Cane,P.A., Kellam,P., Cotten,M., Lemey,P. and Nokes,D.J.
EPI_ISL_2578802	Virology, Graduate School of Medicine, Tohoku University	Virology, Graduate School of Medicine, Tohoku University	Malasao,R., Furuse,Y., Okamoto,M., Dapat,C., Saito,M., Saito-Obata,M., Tamaki,R., Segubre-Mercado,E., Lupisan,S. and Oshitani,H.
EPI_ISL_2578803	Department of Pediatrics, Center of Excellence in Clinical Virology, Chulalongkorn	Department of Pediatrics, Center of Excellence in Clinical Virology, Chulalongkorn	Thongpan,I.
EPI_ISL_2578804, EPI_ISL_2578805, EPI_ISL_2578806, EPI_ISL_2578807, EPI_ISL_2578808	Epidemiology and Demography Department, KEMRI-Wellcome Trust Research Programme	Epidemiology and Demography Department, KEMRI-Wellcome Trust Research Programme	Otieno,J.R., Kamau,E.M., Oketch,J.W., Ngoi,J.M., Agoti,C.N., Gichuki,A.M., Otieno,G.P., Ngama,M., Cane,P.A., Kellam,P., Cotten,M., Lemey,P. and Nokes,D.J.
EPI_ISL_2578809, EPI_ISL_2578810	Virology, Graduate School of Medicine, Tohoku University	Virology, Graduate School of Medicine, Tohoku University	Malasao,R., Furuse,Y., Okamoto,M., Dapat,C., Saito,M., Saito-Obata,M., Tamaki,R., Segubre-Mercado,E., Lupisan,S. and Oshitani,H.
EPI_ISL_2578811, EPI_ISL_2578812, EPI_ISL_2578813	Marie Bashir Institute for Infectious Diseases and Biosecurity & Sydney Medical School, The University of Sydney, Westmead Institute for Medical Research	Marie Bashir Institute for Infectious Diseases and Biosecurity & Sydney Medical School, The University of Sydney, Westmead Institute for Medical Research	Eden,J.-S., Kok,J., Dwyer,D.E., Fernandez,M., Carter,I. and Holmes,E.C.
EPI_ISL_2578814, EPI_ISL_2578815	Epidemiology and Demography Department, KEMRI-Wellcome Trust Research Programme	Epidemiology and Demography Department, KEMRI-Wellcome Trust Research Programme	Otieno,J.R., Kamau,E.M., Oketch,J.W., Ngoi,J.M., Agoti,C.N., Gichuki,A.M., Otieno,G.P., Ngama,M., Cane,P.A., Kellam,P., Cotten,M., Lemey,P. and Nokes,D.J.
EPI_ISL_2578816	J. Craig Venter Institute	J. Craig Venter Institute	Tan,G., Pickett,B., Fedorova,N., Amedeo,P., Hu,L., Christensen,J., Miller,J., Durbin,A., Williams,T., Arumemi,F., Cadiz,C., Alanis,R., Balmseda,A., Wiliams,T., Schiller,A., Patel,M., Kubale,J. and Gordon,A.
EPI_ISL_2578817	Department of Pediatrics, Center of Excellence in Clinical Virology, Chulalongkorn	Department of Pediatrics, Center of Excellence in Clinical Virology, Chulalongkorn	Thongpan,I.
EPI_ISL_2578818, EPI_ISL_2578819, EPI_ISL_2578820, EPI_ISL_2578821	Epidemiology and Demography Department, KEMRI-Wellcome Trust Research Programme	Epidemiology and Demography Department, KEMRI-Wellcome Trust Research Programme	Otieno,J.R., Kamau,E.M., Oketch,J.W., Ngoi,J.M., Agoti,C.N., Gichuki,A.M., Otieno,G.P., Ngama,M., Cane,P.A., Kellam,P., Cotten,M., Lemey,P. and Nokes,D.J.
EPI_ISL_2578825, EPI_ISL_2578826	Marie Bashir Institute for Infectious Diseases and Biosecurity & Sydney Medical School, The University of Sydney, Westmead Institute for Medical Research	Marie Bashir Institute for Infectious Diseases and Biosecurity & Sydney Medical School, The University of Sydney, Westmead Institute for Medical Research	Eden,J.-S., Kok,J., Dwyer,D.E., Fernandez,M., Carter,I. and Holmes,E.C.
EPI_ISL_2579776	Center for Infectious Diseases, School of Public Health, University of Texas Health Science Center	Center for Infectious Diseases, School of Public Health, University of Texas Health Science Center	Bahl,J., Hixson,J., Kim,D.-K., Qiu,X., Piedra,P.A., Piedra,F.-A., Avadhanula,V. and Machado,A.A.
EPI_ISL_2579857	Influenza Group, National Institute of Virology	Influenza Group, National Institute of Virology	Choudhary,M.L., Wadhwa,B., Jadhav,S.M., Chadha,M.S. and Mourya,D.T.
EPI_ISL_2579858, EPI_ISL_2579859, EPI_ISL_2579860	Center for Infectious Diseases, School of Public Health, University of Texas Health Science Center	Center for Infectious Diseases, School of Public Health, University of Texas Health Science Center	Bahl,J., Hixson,J., Kim,D.-K., Qiu,X., Piedra,P.A., Piedra,F.-A., Avadhanula,V. and Machado,A.A.
EPI_ISL_2579886, EPI_ISL_2579887, EPI_ISL_2579888, EPI_ISL_2579889, EPI_ISL_2579890, EPI_ISL_2579891, EPI_ISL_2579892, EPI_ISL_2579894, EPI_ISL_2579895, EPI_ISL_2579896, EPI_ISL_2579897			
see above	Broad Institute of MIT & Harvard	Broad Institute of MIT & Harvard	Newman,R.M., Zody,M.C., DeVincenzo,J.P., Grad,Y., Lipsitch,M., Murphy,R., Fitzgerald,M., Young,S., Gargeya,S., Poon,T.W., Charlebois,P., Weiner,B., Yang,X., Piper,M.E., McCowan,C., Ireland,A., Levin,J., Malboeuf,C., Qu,J., Chapman,S.B., Murphy,C., Wortman,J., Nusbaum,C. and Birren,B.
EPI_ISL_2579900	J. Craig Venter Institute	J. Craig Venter Institute	Shrivastava,S., Halpin,R.A., Puri,V., Fedorova,N.B., Stockwell,T., Amedeo,P., Katzel,D., Schobel,S., Pickett,B.E., Moore,M., Chappell,J., Larkin,E., Wentworth,D.E., Anderson,L.J. and Hartert,T.
EPI_ISL_2582147	Experimental Pathology, Immunology, and Microbiology, American University of Beirut	Experimental Pathology, Immunology, and Microbiology, American University of Beirut	Hamdan,F., Ezzeddine,A., Elbahesh,H. and Zaraket,H.
EPI_ISL_2582157, EPI_ISL_2582160, EPI_ISL_2582161	Epidemiology and Demography Department, KEMRI-Wellcome Trust Research Programme	Epidemiology and Demography Department, KEMRI-Wellcome Trust Research Programme	Otieno,J.R., Kamau,E.M., Oketch,J.W., Ngoi,J.M., Agoti,C.N., Gichuki,A.M., Otieno,G.P., Ngama,M., Cane,P.A., Kellam,P., Cotten,M., Lemey,P. and Nokes,D.J.
EPI_ISL_2582166	Virology, Graduate School of Medicine, Tohoku University	Virology, Graduate School of Medicine, Tohoku University	Malasao,R., Furuse,Y., Okamoto,M., Dapat,C., Saito,M., Saito-Obata,M., Tamaki,R., Segubre-Mercado,E., Lupisan,S. and Oshitani,H.
EPI_ISL_2582167	Lab Medicine, UW	Lab Medicine, UW	Greninger,A.L., Makhsous,N., Kuypers,J.M., Shean,R.C. and Jerome,K.R.
EPI_ISL_2582169	Pediatrics - Infectious Diseases, Medical College of Wisconsin	Pediatrics - Infectious Diseases, Medical College of Wisconsin	Rebuffo-Scheer,C., Bose,M.E., He,J., Khaja,S., Ulatowski,M., Beck,E.T., Fan,J., Kumar,S., Nelson,M.I. and Henrickson,K.J.
EPI_ISL_2582173	Virology, Graduate School of Medicine, Tohoku University	Virology, Graduate School of Medicine, Tohoku University	Malasao,R., Furuse,Y., Okamoto,M., Dapat,C., Saito,M., Saito-Obata,M., Tamaki,R., Segubre-Mercado,E., Lupisan,S. and Oshitani,H.
EPI_ISL_2582176	Laboratory Medicine, UW Virology	Laboratory Medicine, UW Virology	Lin,M.J., Tait,A. and Greninger,A.L.
EPI_ISL_2582177	Pediatrics, University of New Mexico	Pediatrics, University of New Mexico	Kothari,A., Kennedy,J.L., Schwalm,K.C., Putt,C., Denson,J.L. and Dinwiddie,D.L.
EPI_ISL_2582180	Broad Institute of MIT & Harvard	Broad Institute of MIT & Harvard	Newman,R.M., Zody,M.C., DeVincenzo,J.P., Grad,Y., Lipsitch,M., Murphy,R., Fitzgerald,M., Young,S., Gargeya,S., Poon,T.W., Charlebois,P., Weiner,B., Yang,X., Piper,M.E., McCowan,C., Ireland,A., Levin,J., Malboeuf,C., Qu,J., Chapman,S.B., Murphy,C., Wortman,J., Nusbaum,C. and Birren,B.
EPI_ISL_2582181, EPI_ISL_2582183	Virology, Graduate School of Medicine, Tohoku University	Virology, Graduate School of Medicine, Tohoku University	Malasao,R., Furuse,Y., Okamoto,M., Dapat,C., Saito,M., Saito-Obata,M., Tamaki,R., Segubre-Mercado,E., Lupisan,S. and Oshitani,H.
EPI_ISL_2582187, EPI_ISL_2582196	J. Craig Venter Institute	J. Craig Venter Institute	Tan,G., Pickett,B., Fedorova,N., Amedeo,P., Isom,R., Hu,L., Christensen,J., Miller,J., Novotny,M., Durbin,A., Rocchi,I., Williams,T., Arumemi,F. and Das,S.
EPI_ISL_2582197	Lab Medicine, UW	Lab Medicine, UW	Greninger,A.L., Makhsous,N., Kuypers,J.M., Shean,R.C. and Jerome,K.R.
EPI_ISL_2582201	Epidemiology and Demography Department, KEMRI-Wellcome Trust Research Programme	Epidemiology and Demography Department, KEMRI-Wellcome Trust Research Programme	Otieno,J.R., Kamau,E.M., Oketch,J.W., Ngoi,J.M., Agoti,C.N., Gichuki,A.M., Otieno,G.P., Ngama,M., Cane,P.A., Kellam,P., Cotten,M., Lemey,P. and Nokes,D.J.
EPI_ISL_2582221	J. Craig Venter Institute	J. Craig Venter Institute	Tan,G., Pickett,B., Fedorova,N., Amedeo,P., Hu,L., Christensen,J., Miller,J., Durbin,A., Williams,T., Arumemi,F., Cadiz,C., Alanis,R., Balmseda,A., Wiliams,T., Schiller,A., Patel,M., Kubale,J. and Gordon,A.
EPI_ISL_2582223, EPI_ISL_2582226, EPI_ISL_2582227	Marie Bashir Institute for Infectious Diseases and Biosecurity & Sydney Medical School, The University of Sydney, Westmead Institute for Medical Research	Marie Bashir Institute for Infectious Diseases and Biosecurity & Sydney Medical School, The University of Sydney, Westmead Institute for Medical Research	Eden,J.-S., Kok,J., Dwyer,D.E., Fernandez,M., Carter,I. and Holmes,E.C.
EPI_ISL_2582230, EPI_ISL_2582231	J. Craig Venter Institute	J. Craig Venter Institute	Tan,G., Pickett,B., Fedorova,N., Amedeo,P., Hu,L., Christensen,J., Miller,J., Durbin,A., Williams,T., Arumemi,F., Cadiz,C., Alanis,R., Balmseda,A., Wiliams,T., Schiller,A., Patel,M., Kubale,J. and Gordon,A.
EPI_ISL_2582233	Laboratory Medicine, UW Virology	Laboratory Medicine, UW Virology	Lin,M.J., Tait,A. and Greninger,A.L.

EPI_ISL_2582235, EPI_ISL_2582238	J. Craig Venter Institute	J. Craig Venter Institute	Tan,G., Pickett,B., Fedorova,N., Amedeo,P., Hu,L., Christensen,J., Miller,J., Durbin,A., Williams,T., Arumemi,F., Cadiz,C., Alanis,R., Balmseda,A., Wiliams,T., Schiller,A., Patel,M., Kubale,J. and Gordon,A.
EPI_ISL_2582239, EPI_ISL_2582241, EPI_ISL_2582244	Marie Bashir Institute for Infectious Diseases and Biosecurity & Sydney Medical School, The University of Sydney, Westmead Institute for Medical Research	Marie Bashir Institute for Infectious Diseases and Biosecurity & Sydney Medical School, The University of Sydney, Westmead Institute for Medical Research	Eden,J.-S., Kok,J., Dwyer,D.E., Fernandez,M., Carter,I. and Holmes,E.C.
EPI_ISL_2582246	Pediatrics, University of New Mexico	Pediatrics, University of New Mexico	Kothari,A., Kennedy,J.L., Schwalm,K.C., Putt,C., Denson,J.L. and Dinwiddie,D.L.
EPI_ISL_2582247	Laboratory Medicine, UW Virology	Laboratory Medicine, UW Virology	Lin,M.J., Tait,A. and Greninger,A.L.
EPI_ISL_2582250, EPI_ISL_2582251, EPI_ISL_2582253	Marie Bashir Institute for Infectious Diseases and Biosecurity & Sydney Medical School, The University of Sydney, Westmead Institute for Medical Research	Marie Bashir Institute for Infectious Diseases and Biosecurity & Sydney Medical School, The University of Sydney, Westmead Institute for Medical Research	Eden,J.-S., Kok,J., Dwyer,D.E., Fernandez,M., Carter,I. and Holmes,E.C.
EPI_ISL_2582256, EPI_ISL_2582257, EPI_ISL_2582260	Epidemiology and Demography Department, KEMRI-Wellcome Trust Research Programme	Epidemiology and Demography Department, KEMRI-Wellcome Trust Research Programme	Otieno,J.R., Kamau,E.M., Oketch,J.W., Ngoi,J.M., Agoti,C.N., Gichuki,A.M., Otieno,G.P., Ngama,M., Cane,P.A., Kellam,P., Cotten,M., Lemey,P. and Nokes,D.J.
EPI_ISL_2582261	Pediatrics, University of New Mexico	Pediatrics, University of New Mexico	Kothari,A., Kennedy,J.L., Schwalm,K.C., Putt,C., Denson,J.L. and Dinwiddie,D.L.
EPI_ISL_2582264, EPI_ISL_2582265	Marie Bashir Institute for Infectious Diseases and Biosecurity & Sydney Medical School, The University of Sydney, Westmead Institute for Medical Research	Marie Bashir Institute for Infectious Diseases and Biosecurity & Sydney Medical School, The University of Sydney, Westmead Institute for Medical Research	Eden,J.-S., Kok,J., Dwyer,D.E., Fernandez,M., Carter,I. and Holmes,E.C.
EPI_ISL_2582267	Epidemiology and Demography Department, KEMRI-Wellcome Trust Research Programme	Epidemiology and Demography Department, KEMRI-Wellcome Trust Research Programme	Otieno,J.R., Kamau,E.M., Oketch,J.W., Ngoi,J.M., Agoti,C.N., Gichuki,A.M., Otieno,G.P., Ngama,M., Cane,P.A., Kellam,P., Cotten,M., Lemey,P. and Nokes,D.J.
EPI_ISL_2582271	J. Craig Venter Institute	J. Craig Venter Institute	Tan,G., Pickett,B., Fedorova,N., Amedeo,P., Hu,L., Christensen,J., Miller,J., Durbin,A., Williams,T., Arumemi,F., Cadiz,C., Alanis,R., Balmseda,A., Wiliams,T., Schiller,A., Patel,M., Kubale,J. and Gordon,A.
EPI_ISL_2582274, EPI_ISL_2582275, EPI_ISL_2582278	Department of Experimental Modeling and Pathogenesis of Infectious Diseases, Federal Research Center of Fundamental and Translational Medicine	Department of Experimental Modeling and Pathogenesis of Infectious Diseases, Federal Research Center of Fundamental and Translational Medicine	Dubovitskiy,N.A., Sobolev,I.A., Kurskaya,O.G., Sharshov,K.A., Anoshina,A.V., Leonova,N.V., Murashkina,T.A., Solomatina,M.V., Derko,A.A., Saroyan,T.A., Kabilov,M.R., Alikina,T.Y. and Shestopalov,A.M.
EPI_ISL_2582279, EPI_ISL_2582281, EPI_ISL_2582284	Epidemiology and Demography Department, KEMRI-Wellcome Trust Research Programme	Epidemiology and Demography Department, KEMRI-Wellcome Trust Research Programme	Otieno,J.R., Kamau,E.M., Oketch,J.W., Ngoi,J.M., Agoti,C.N., Gichuki,A.M., Otieno,G.P., Ngama,M., Cane,P.A., Kellam,P., Cotten,M., Lemey,P. and Nokes,D.J.
EPI_ISL_2582285	Department of Pediatrics, Center of Excellence in Clinical Virology, Chulalongkorn	Department of Pediatrics, Center of Excellence in Clinical Virology, Chulalongkorn	Thongpan,I.
EPI_ISL_2582292	Epidemiology and Demography Department, KEMRI-Wellcome Trust Research Programme	Epidemiology and Demography Department, KEMRI-Wellcome Trust Research Programme	Otieno,J.R., Kamau,E.M., Oketch,J.W., Ngoi,J.M., Agoti,C.N., Gichuki,A.M., Otieno,G.P., Ngama,M., Cane,P.A., Kellam,P., Cotten,M., Lemey,P. and Nokes,D.J.
EPI_ISL_2582296	Department of Pediatrics, Center of Excellence in Clinical Virology, Chulalongkorn	Department of Pediatrics, Center of Excellence in Clinical Virology, Chulalongkorn	Thongpan,I.
EPI_ISL_2582298, EPI_ISL_2582299, EPI_ISL_2582302, EPI_ISL_2582303	J. Craig Venter Institute	J. Craig Venter Institute	Shabman,R., Fedorova,N., Puri,V., Shrivastava,S., Amedeo,P., Isom,R., Hu,L., Pickett,B., Novotny,M., Durbin,A., Rocchi,I., Williams,T., Hall,C.B., Tesini,B.L., Schnabel,K.C., Walsh,E.E. and Caserta,M.
EPI_ISL_2582306, EPI_ISL_2582307, EPI_ISL_2582309, EPI_ISL_2582312, EPI_ISL_2582313, EPI_ISL_2582316, EPI_ISL_2582318, EPI_ISL_2582319, EPI_ISL_2582321, EPI_ISL_2582324, EPI_ISL_2582325, EPI_ISL_2582327, EPI_ISL_2582330, EPI_ISL_2582332, EPI_ISL_2582333, EPI_ISL_2582335, EPI_ISL_2582337, EPI_ISL_2582340, EPI_ISL_2582341, EPI_ISL_2582342, EPI_ISL_2582344, EPI_ISL_2582345, EPI_ISL_2582348, EPI_ISL_2582349, EPI_ISL_2582351, EPI_ISL_2582354, EPI_ISL_2582355, EPI_ISL_2582357, EPI_ISL_2582360, EPI_ISL_2582361, EPI_ISL_2582363, EPI_ISL_2582366, EPI_ISL_2582367, EPI_ISL_2582370, EPI_ISL_2582371, EPI_ISL_2582373, EPI_ISL_2582375, EPI_ISL_2582378, EPI_ISL_2582379, EPI_ISL_2582381, EPI_ISL_2582383, EPI_ISL_2582386, EPI_ISL_2582387, EPI_ISL_2582389, EPI_ISL_2582391, EPI_ISL_2582393, EPI_ISL_2582395, EPI_ISL_2582397, EPI_ISL_2582399, EPI_ISL_2582402, EPI_ISL_2582403, EPI_ISL_2582405, EPI_ISL_2582407, EPI_ISL_2582410, EPI_ISL_2582411, EPI_ISL_2582413, EPI_ISL_2582416, EPI_ISL_2582417, EPI_ISL_2582419, EPI_ISL_2582422, EPI_ISL_2582423, EPI_ISL_2582426, EPI_ISL_2582427, EPI_ISL_2582430, EPI_ISL_2582431, EPI_ISL_2582434, EPI_ISL_2582435, EPI_ISL_2582438, EPI_ISL_2582439, EPI_ISL_2582442, EPI_ISL_2582443, EPI_ISL_2582445, EPI_ISL_2582448, EPI_ISL_2582449, EPI_ISL_2582451, EPI_ISL_2582456, EPI_ISL_2582457, EPI_ISL_2582460, EPI_ISL_2582461, EPI_ISL_2582463, EPI_ISL_2582465, EPI_ISL_2582468, EPI_ISL_2582469, EPI_ISL_2582471, EPI_ISL_2582476, EPI_ISL_2582477, EPI_ISL_2582479, EPI_ISL_2582481, EPI_ISL_2582484, EPI_ISL_2582485, EPI_ISL_2582487, EPI_ISL_2582490, EPI_ISL_2582491, EPI_ISL_2582495, EPI_ISL_2582497, EPI_ISL_2582500, EPI_ISL_2582502, EPI_ISL_2582503, EPI_ISL_2582505, EPI_ISL_2582508, EPI_ISL_2582509			
see above	Broad Institute of MIT & Harvard	Broad Institute of MIT & Harvard	Newman,R.M., Zody,M.C., DeVincenzo,J.P., Grad,Y., Lipsitch,M., Murphy,R., Fitzgerald,M., Young,S., Gargeya,S., Poon,T.W., Charlebois,P., Weiner,B., Yang,X., Piper,M.E., McCowan,C., Ireland,A., Levin,J., Malboeuf,C., Qu,J., Chapman,S.B., Murphy,C., Wortman,J., Nusbaum,C. and Birren,B.
EPI_ISL_2582510, EPI_ISL_2582512	Marie Bashir Institute for Infectious Diseases and Biosecurity & Sydney Medical School, The University of Sydney, Westmead Institute for Medical Research	Marie Bashir Institute for Infectious Diseases and Biosecurity & Sydney Medical School, The University of Sydney, Westmead Institute for Medical Research	Eden,J.-S., Kok,J., Dwyer,D.E., Fernandez,M., Carter,I. and Holmes,E.C.
EPI_ISL_2582513, EPI_ISL_2582515, EPI_ISL_2582517, EPI_ISL_2582519	Broad Institute of MIT & Harvard	Broad Institute of MIT & Harvard	Newman,R.M., Zody,M.C., DeVincenzo,J.P., Grad,Y., Lipsitch,M., Murphy,R., Fitzgerald,M., Young,S., Gargeya,S., Poon,T.W., Charlebois,P., Weiner,B., Yang,X., Piper,M.E., McCowan,C., Ireland,A., Levin,J., Malboeuf,C., Qu,J., Chapman,S.B., Murphy,C., Wortman,J., Nusbaum,C. and Birren,B.
EPI_ISL_2582521	J. Craig Venter Institute	J. Craig Venter Institute	Shabman,R., Fedorova,N., Puri,V., Shrivastava,S., Amedeo,P., Isom,R., Hu,L., Pickett,B., Novotny,M., Durbin,A., Rocchi,I., Williams,T., Hall,C.B., Tesini,B.L., Schnabel,K.C., Walsh,E.E. and Caserta,M.
EPI_ISL_2582536	Laboratory Medicine, UW Virology	Laboratory Medicine, UW Virology	Lin,M.J., Tait,A. and Greninger,A.L.
EPI_ISL_2582538, EPI_ISL_2582540, EPI_ISL_2582541	Lab Medicine, UW	Lab Medicine, UW	Greninger,A.L., Makhsous,N., Kuypers,J.M., Shean,R.C. and Jerome,K.R.
EPI_ISL_2582543, EPI_ISL_2582545, EPI_ISL_2582547	J. Craig Venter Institute	J. Craig Venter Institute	Tan,G., Pickett,B., Fedorova,N., Amedeo,P., Isom,R., Hu,L., Christensen,J., Miller,J., Novotny,M., Durbin,A., Rocchi,I., Williams,T., Arumemi,F. and Das,S.
EPI_ISL_2582551	Lab Medicine, UW	Lab Medicine, UW	Greninger,A.L., Makhsous,N., Kuypers,J.M., Shean,R.C. and Jerome,K.R.
EPI_ISL_2582553	J. Craig Venter Institute	J. Craig Venter Institute	Tan,G., Pickett,B., Fedorova,N., Amedeo,P., Isom,R., Hu,L., Christensen,J., Miller,J., Novotny,M., Durbin,A., Rocchi,I., Williams,T., Arumemi,F. and Das,S.
EPI_ISL_2582561	Central Laboratory, Guangzhou Women and Children's Medical Center	Central Laboratory, Guangzhou Women and Children's Medical Center	Xie,J.H., Zhu,B., Zhong,J.Y., Chen,Y. and Zhang,Y.Y.
EPI_ISL_2582563	The Second Department, Lanzhou Institute of Biological Products Co	The Second Department, Lanzhou Institute of Biological Products Co	Zhu,C., Fu,S., Yu,L. and Zhou,X.
EPI_ISL_2582565, EPI_ISL_2582567	Epidemiology and Demography Department, KEMRI-Wellcome Trust Research Programme	Epidemiology and Demography Department, KEMRI-Wellcome Trust Research Programme	Otieno,J.R., Kamau,E.M., Oketch,J.W., Ngoi,J.M., Agoti,C.N., Gichuki,A.M., Otieno,G.P., Ngama,M., Cane,P.A., Kellam,P., Cotten,M., Lemey,P. and Nokes,D.J.
EPI_ISL_2582569, EPI_ISL_2582571	J. Craig Venter Institute	J. Craig Venter Institute	Tan,G., Pickett,B., Fedorova,N., Amedeo,P., Isom,R., Hu,L., Christensen,J., Miller,J., Novotny,M., Durbin,A., Rocchi,I., Williams,T., Arumemi,F. and Das,S.
EPI_ISL_2582573	Pediatrics - Infectious Diseases, Medical College of Wisconsin	Pediatrics - Infectious Diseases, Medical College of Wisconsin	Rebuffo-Scheer,C., Bose,M.E., He,J., Khaja,S., Ulatowski,M., Beck,E.T., Fan,J., Kumar,S., Nelson,M.I. and Henrickson,K.J.
EPI_ISL_2582577, EPI_ISL_2582579, EPI_ISL_2582581, EPI_ISL_2582583, EPI_ISL_2582585, EPI_ISL_2582587, EPI_ISL_2582588, EPI_ISL_2582590, EPI_ISL_2582592, EPI_ISL_2582594, EPI_ISL_2582596, EPI_ISL_2582598, EPI_ISL_2582600, EPI_ISL_2582601, EPI_ISL_2582603, EPI_ISL_2582605, EPI_ISL_2582607, EPI_ISL_2582609, EPI_ISL_2582610, EPI_ISL_2582612, EPI_ISL_2582614, EPI_ISL_2582616, EPI_ISL_2582618			
see above	Broad Institute of MIT & Harvard	Broad Institute of MIT & Harvard	Newman,R.M., Zody,M.C., DeVincenzo,J.P., Grad,Y., Lipsitch,M., Murphy,R., Fitzgerald,M., Young,S., Gargeya,S., Poon,T.W., Charlebois,P., Weiner,B., Yang,X., Piper,M.E., McCowan,C., Ireland,A., Levin,J., Malboeuf,C., Qu,J., Chapman,S.B., Murphy,C., Wortman,J., Nusbaum,C. and Birren,B.
EPI_ISL_2582621	Academy of Military Medical Sciences, Institute of Microbiology and Epidemiology	Academy of Military Medical Sciences, Institute of Microbiology and Epidemiology	Gu,H.J., Sun,S.J., Chen,R. and Yang,P.H.
EPI_ISL_2582623, EPI_ISL_2582625	Broad Institute of MIT & Harvard	Broad Institute of MIT & Harvard	Newman,R.M., Zody,M.C., DeVincenzo,J.P., Grad,Y., Lipsitch,M., Murphy,R., Fitzgerald,M., Young,S., Gargeya,S., Poon,T.W., Charlebois,P., Weiner,B.,

			Yang,X., Piper,M.E., McCowan,C., Ireland,A., Levin,J., Malboeuf,C., Qu,J., Chapman,S.B., Murphy,C., Wortman,J., Nusbaum,C. and Birren,B.
EPI_ISL_2582630	Pediatrics - Infectious Diseases, Medical College of Wisconsin	Pediatrics - Infectious Diseases, Medical College of Wisconsin	Rebuffo-Scheer,C., Bose,M.E., He,J., Khaja,S., Ulatowski,M., Beck,E.T., Fan,J., Kumar,S., Nelson,M.I. and Henrickson,K.J.
EPI_ISL_2582631, EPI_ISL_2582633	J. Craig Venter Institute	J. Craig Venter Institute	Shabman,R., Fedorova,N., Puri,V., Shrivastava,S., Amedeo,P., Isom,R., Hu,L., Pickett,B., Novotny,M., Durbin,A., Rocchi,I., Williams,T., Hall,C.B., Tesini,B.L., Schnabel,K.C., Walsh,E.E. and Caserta,M.
EPI_ISL_2582635	Broad Institute of MIT & Harvard	Broad Institute of MIT & Harvard	Newman,R.M., Zody,M.C., DeVincenzo,J.P., Grad,Y., Lipsitch,M., Murphy,R., Fitzgerald,M., Young,S., Gargeya,S., Poon,T.W., Charlebois,P., Weiner,B., Yang,X., Piper,M.E., McCowan,C., Ireland,A., Levin,J., Malboeuf,C., Qu,J., Chapman,S.B., Murphy,C., Wortman,J., Nusbaum,C. and Birren,B.
EPI_ISL_2582641, EPI_ISL_2582644, EPI_ISL_2582646	Marie Bashir Institute for Infectious Diseases and Biosecurity & Sydney Medical School, The University of Sydney, Westmead Institute for Medical Research	Marie Bashir Institute for Infectious Diseases and Biosecurity & Sydney Medical School, The University of Sydney, Westmead Institute for Medical Research	Eden,J.-S., Kok,J., Dwyer,D.E., Fernandez,M., Carter,I. and Holmes,E.C.
EPI_ISL_2582648, EPI_ISL_2582650, EPI_ISL_2582653	Pediatrics, University of New Mexico	Pediatrics, University of New Mexico	Kothari,A., Kennedy,J.L., Schwalm,K.C., Putt,C., Denson,J.L. and Dinwiddie,D.L.
EPI_ISL_2582669, EPI_ISL_2582670, EPI_ISL_2582673	Epidemiology and Demography Department, KEMRI-Wellcome Trust Research Programme	Epidemiology and Demography Department, KEMRI-Wellcome Trust Research Programme	Otieno,J.R., Kamau,E.M., Oketch,J.W., Ngoi,J.M., Agoti,C.N., Gichuki,A.M., Otieno,G.P., Ngama,M., Cane,P.A., Kellam,P., Cotten,M., Lemey,P. and Nokes,D.J.
EPI_ISL_2582675	Suguru Takeuchi Nagoya University Graduate School of Medicine, Department of Pediatrics	Suguru Takeuchi Nagoya University Graduate School of Medicine, Department of Pediatrics	Takeuchi,S., Kawada,J. and Ito,Y.
EPI_ISL_2582678	Epidemiology and Demography Department, KEMRI-Wellcome Trust Research Programme	Epidemiology and Demography Department, KEMRI-Wellcome Trust Research Programme	Otieno,J.R., Kamau,E.M., Oketch,J.W., Ngoi,J.M., Agoti,C.N., Gichuki,A.M., Otieno,G.P., Ngama,M., Cane,P.A., Kellam,P., Cotten,M., Lemey,P. and Nokes,D.J.
EPI_ISL_2582681	Experimental Pathology, Immunology, and Microbiology, American University of Beirut	Experimental Pathology, Immunology, and Microbiology, American University of Beirut	Ezzeddine,A.M.
EPI_ISL_2582683, EPI_ISL_2582686, EPI_ISL_2582688, EPI_ISL_2582691, EPI_ISL_2582694, EPI_ISL_2582696, EPI_ISL_2582699, EPI_ISL_2582701	Broad Institute of MIT & Harvard	Broad Institute of MIT & Harvard	Newman,R.M., Zody,M.C., DeVincenzo,J.P., Grad,Y., Lipsitch,M., Murphy,R., Fitzgerald,M., Young,S., Gargeya,S., Poon,T.W., Charlebois,P., Weiner,B., Yang,X., Piper,M.E., McCowan,C., Ireland,A., Levin,J., Malboeuf,C., Qu,J., Chapman,S.B., Murphy,C., Wortman,J., Nusbaum,C. and Birren,B.
EPI_ISL_2582703	J. Craig Venter Institute	J. Craig Venter Institute	Shabman,R., Fedorova,N., Puri,V., Shrivastava,S., Amedeo,P., Isom,R., Hu,L., Pickett,B., Novotny,M., Durbin,A., Rocchi,I., Williams,T., Hall,C.B., Tesini,B.L., Schnabel,K.C., Walsh,E.E. and Caserta,M.
EPI_ISL_2582706, EPI_ISL_2582709, EPI_ISL_2582711, EPI_ISL_2582714, EPI_ISL_2582716, EPI_ISL_2582719, EPI_ISL_2582722, EPI_ISL_2582724, EPI_ISL_2582727, EPI_ISL_2582729, EPI_ISL_2582732, EPI_ISL_2582735, EPI_ISL_2582738, EPI_ISL_2582740, EPI_ISL_2582743, EPI_ISL_2582745, EPI_ISL_2582747, EPI_ISL_2582750, EPI_ISL_2582751, EPI_ISL_2582756, EPI_ISL_2582758, EPI_ISL_2582760, EPI_ISL_2582762, EPI_ISL_2582764			
see above	Broad Institute of MIT & Harvard	Broad Institute of MIT & Harvard	Newman,R.M., Zody,M.C., DeVincenzo,J.P., Grad,Y., Lipsitch,M., Murphy,R., Fitzgerald,M., Young,S., Gargeya,S., Poon,T.W., Charlebois,P., Weiner,B., Yang,X., Piper,M.E., McCowan,C., Ireland,A., Levin,J., Malboeuf,C., Qu,J., Chapman,S.B., Murphy,C., Wortman,J., Nusbaum,C. and Birren,B.
EPI_ISL_2582766	Infectious Disease Initiative, Broad Institute	Infectious Disease Initiative, Broad Institute	Newman,R.M., Zody,M.C., DeVincenzo,J.P., Grad,Y., Lipsitch,M., Murphy,R., Fitzgerald,M., Young,S., Gargeya,S., Poon,T.W., Charlebois,P., Weiner,B., Yang,X., Piper,M.E., McCowan,C., Ireland,A., Levin,J., Malboeuf,C., Qu,J., Chapman,S.B., Murphy,C., Wortman,J., Nusbaum,C. and Birren,B.
EPI_ISL_2582768	Broad Institute of MIT & Harvard	Broad Institute of MIT & Harvard	Newman,R.M., Zody,M.C., DeVincenzo,J.P., Grad,Y., Lipsitch,M., Murphy,R., Fitzgerald,M., Young,S., Gargeya,S., Poon,T.W., Charlebois,P., Weiner,B., Yang,X., Piper,M.E., McCowan,C., Ireland,A., Levin,J., Malboeuf,C., Qu,J., Chapman,S.B., Murphy,C., Wortman,J., Nusbaum,C. and Birren,B.
EPI_ISL_2582771	Marie Bashir Institute for Infectious Diseases and Biosecurity & Sydney Medical School, The University of Sydney, Westmead Institute for Medical Research	Marie Bashir Institute for Infectious Diseases and Biosecurity & Sydney Medical School, The University of Sydney, Westmead Institute for Medical Research	Eden,J.-S., Kok,J., Dwyer,D.E., Fernandez,M., Carter,I. and Holmes,E.C.
EPI_ISL_2582775, EPI_ISL_2582778	J. Craig Venter Institute	J. Craig Venter Institute	Shabman,R., Fedorova,N., Puri,V., Shrivastava,S., Amedeo,P., Isom,R., Hu,L., Pickett,B., Novotny,M., Durbin,A., Rocchi,I., Williams,T., Hall,C.B., Tesini,B.L., Schnabel,K.C., Walsh,E.E. and Caserta,M.
EPI_ISL_2582780	Broad Institute of MIT & Harvard	Broad Institute of MIT & Harvard	Newman,R.M., Zody,M.C., DeVincenzo,J.P., Grad,Y., Lipsitch,M., Murphy,R., Fitzgerald,M., Young,S., Gargeya,S., Poon,T.W., Charlebois,P., Weiner,B., Yang,X., Piper,M.E., McCowan,C., Ireland,A., Levin,J., Malboeuf,C., Qu,J., Chapman,S.B., Murphy,C., Wortman,J., Nusbaum,C. and Birren,B.
EPI_ISL_2582782	J. Craig Venter Institute	J. Craig Venter Institute	Shabman,R., Fedorova,N., Puri,V., Shrivastava,S., Amedeo,P., Isom,R., Hu,L., Pickett,B., Novotny,M., Durbin,A., Rocchi,I., Williams,T., Hall,C.B., Tesini,B.L., Schnabel,K.C., Walsh,E.E. and Caserta,M.
EPI_ISL_2582784	Broad Institute of MIT & Harvard	Broad Institute of MIT & Harvard	Newman,R.M., Zody,M.C., DeVincenzo,J.P., Grad,Y., Lipsitch,M., Murphy,R., Fitzgerald,M., Young,S., Gargeya,S., Poon,T.W., Charlebois,P., Weiner,B., Yang,X., Piper,M.E., McCowan,C., Ireland,A., Levin,J., Malboeuf,C., Qu,J., Chapman,S.B., Murphy,C., Wortman,J., Nusbaum,C. and Birren,B.
EPI_ISL_2582787	Lab Medicine, UW	Lab Medicine, UW	Greninger,A.L., Makhsous,N., Kuypers,J.M., Shean,R.C. and Jerome,K.R.
EPI_ISL_2582789	Epidemiology and Demography Department, KEMRI-Wellcome Trust Research Programme	Epidemiology and Demography Department, KEMRI-Wellcome Trust Research Programme	Otieno,J.R., Kamau,E.M., Oketch,J.W., Ngoi,J.M., Agoti,C.N., Gichuki,A.M., Otieno,G.P., Ngama,M., Cane,P.A., Kellam,P., Cotten,M., Lemey,P. and Nokes,D.J.
EPI_ISL_2582796	Central Laboratory, Guangzhou Women and Children's Medical Center	Central Laboratory, Guangzhou Women and Children's Medical Center	Xie,J.H., Zhu,B., Zhong,J.Y., Chen,Y. and Zhang,Y.Y.
EPI_ISL_2582798	Lab Medicine, UW	Lab Medicine, UW	Greninger,A.L., Makhsous,N., Kuypers,J.M., Shean,R.C. and Jerome,K.R.
EPI_ISL_2582801	Department of Laboratory Medicine, Lin-Kou Chang-Gung Memorial Hospital	Department of Laboratory Medicine, Lin-Kou Chang-Gung Memorial Hospital	Tsao,K.-C., Gong,Y.-N., Yang,S.-L., Chen,G.-W., Chen,Y.-W., Huang,Y.-C. and Liu,Y.-C.
EPI_ISL_2582804	Epidemiology and Demography Department, KEMRI-Wellcome Trust Research Programme	Epidemiology and Demography Department, KEMRI-Wellcome Trust Research Programme	Otieno,J.R., Kamau,E.M., Oketch,J.W., Ngoi,J.M., Agoti,C.N., Gichuki,A.M., Otieno,G.P., Ngama,M., Cane,P.A., Kellam,P., Cotten,M., Lemey,P. and Nokes,D.J.
EPI_ISL_2582805	J. Craig Venter Institute	J. Craig Venter Institute	Tan,G., Pickett,B., Fedorova,N., Amedeo,P., Isom,R., Hu,L., Christensen,J., Miller,J., Novotny,M., Durbin,A., Rocchi,I., Williams,T., Arumemi,F. and Das,S.
EPI_ISL_2582808, EPI_ISL_2582810, EPI_ISL_2582813, EPI_ISL_2582819, EPI_ISL_2582820, EPI_ISL_2582823, EPI_ISL_2582826	Broad Institute of MIT & Harvard	Broad Institute of MIT & Harvard	Newman,R.M., Zody,M.C., DeVincenzo,J.P., Grad,Y., Lipsitch,M., Murphy,R., Fitzgerald,M., Young,S., Gargeya,S., Poon,T.W., Charlebois,P., Weiner,B., Yang,X., Piper,M.E., McCowan,C., Ireland,A., Levin,J., Malboeuf,C., Qu,J., Chapman,S.B., Murphy,C., Wortman,J., Nusbaum,C. and Birren,B.
EPI_ISL_2582832, EPI_ISL_2582834	Laboratory Medicine, UW Virology	Laboratory Medicine, UW Virology	Lin,M.J., Tait,A. and Greninger,A.L.
EPI_ISL_2582836, EPI_ISL_2582838	Department of Experimental Modeling and Pathogenesis of Infectious Diseases, Federal Research Center of Fundamental and Translational Medicine	Department of Experimental Modeling and Pathogenesis of Infectious Diseases, Federal Research Center of Fundamental and Translational Medicine	Dubovitskiy,N.A., Sobolev,I.A., Kurskaya,O.G., Sharshov,K.A., Anoshina,A.V., Leonova,N.V., Murashkina,T.A., Solomatina,M.V., Derko,A.A., Saroyan,T.A., Kabilov,M.R., Alikina,T.Y. and Shestopalov,A.M.
EPI_ISL_2582841	Marie Bashir Institute for Infectious Diseases and Biosecurity & Sydney Medical School, The University of Sydney, Westmead Institute for Medical Research	Marie Bashir Institute for Infectious Diseases and Biosecurity & Sydney Medical School, The University of Sydney, Westmead Institute for Medical Research	Eden,J.-S., Kok,J., Dwyer,D.E., Fernandez,M., Carter,I. and Holmes,E.C.
EPI_ISL_2582844	Broad Institute of MIT & Harvard	Broad Institute of MIT & Harvard	Newman,R.M., Zody,M.C., DeVincenzo,J.P., Grad,Y., Lipsitch,M., Murphy,R., Fitzgerald,M., Young,S., Gargeya,S., Poon,T.W., Charlebois,P., Weiner,B., Yang,X., Piper,M.E., McCowan,C., Ireland,A., Levin,J., Malboeuf,C., Qu,J., Chapman,S.B., Murphy,C., Wortman,J., Nusbaum,C. and Birren,B.
EPI_ISL_2582847	J. Craig Venter Institute	J. Craig Venter Institute	Shabman,R., Fedorova,N., Puri,V., Shrivastava,S., Amedeo,P., Isom,R., Hu,L., Pickett,B., Novotny,M., Durbin,A., Rocchi,I., Williams,T., Hall,C.B., Tesini,B.L., Schnabel,K.C., Walsh,E.E. and Caserta,M.
EPI_ISL_2582849, EPI_ISL_2582851	Center for Infectious Diseases, School of Public Health,	Center for Infectious Diseases, School of Public Health,	Bahl,J., Hixson,J., Kim,D.-K., Qiu,X., Piedra,P.A., Piedra,F.-A., Avadhanula,V. and Machado,A.A.

	University of Texas Health Science Center	University of Texas Health Science Center	
EPI_ISL_2582854	Broad Institute of MIT & Harvard	Broad Institute of MIT & Harvard	Newman,R.M., Zody,M.C., DeVincenzo,J.P., Grad,Y., Lipsitch,M., Murphy,R., Fitzgerald,M., Young,S., Gargeya,S., Poon,T.W., Charlebois,P., Weiner,B., Yang,X., Piper,M.E., McCowan,C., Ireland,A., Levin,J., Malboeuf,C., Qu,J., Chapman,S.B., Murphy,C., Wortman,J., Nusbaum,C. and Birren,B.
EPI_ISL_2582857, EPI_ISL_2582859, EPI_ISL_2582861	Pediatrics - Infectious Diseases, Medical College of Wisconsin	Pediatrics - Infectious Diseases, Medical College of Wisconsin	Rebuffo-Scheer,C., Bose,M.E., He,J., Khaja,S., Ulatowski,M., Beck,E.T., Fan,J., Kumar,S., Nelson,M.I. and Henrickson,K.J.
EPI_ISL_2582864	Gansu Center for Disease Control and Prevention, Pathogen Laboratory	Gansu Center for Disease Control and Prevention, Pathogen Laboratory	Qiao,R., Chen,J., Wu,H. and Yu,D.
EPI_ISL_2582866	Epidemiology and Demography Department, KEMRI-Wellcome Trust Research Programme	Epidemiology and Demography Department, KEMRI-Wellcome Trust Research Programme	Otieno,J.R., Kamau,E.M., Oketch,J.W., Ngoi,J.M., Agoti,C.N., Gichuki,A.M., Otieno,G.P., Ngama,M., Cane,P.A., Kellam,P., Cotten,M., Lemey,P. and Nokes,D.J.
EPI_ISL_2582869	Pediatrics - Infectious Diseases, Medical College of Wisconsin	Pediatrics - Infectious Diseases, Medical College of Wisconsin	Rebuffo-Scheer,C., Bose,M.E., He,J., Khaja,S., Ulatowski,M., Beck,E.T., Fan,J., Kumar,S., Nelson,M.I. and Henrickson,K.J.
EPI_ISL_2582873, EPI_ISL_2582875	J. Craig Venter Institute	J. Craig Venter Institute	Shabman,R., Fedorova,N., Puri,V., Shrivastava,S., Amedeo,P., Isom,R., Hu,L., Pickett,B., Novotny,M., Durbin,A., Rocchi,I., Williams,T., Hall,C.B., Tesini,B.L., Schnabel,K.C., Walsh,E.E. and Caserta,M.
EPI_ISL_2582878	Broad Institute of MIT & Harvard	Broad Institute of MIT & Harvard	Newman,R.M., Zody,M.C., DeVincenzo,J.P., Grad,Y., Lipsitch,M., Murphy,R., Fitzgerald,M., Young,S., Gargeya,S., Poon,T.W., Charlebois,P., Weiner,B., Yang,X., Piper,M.E., McCowan,C., Ireland,A., Levin,J., Malboeuf,C., Qu,J., Chapman,S.B., Murphy,C., Wortman,J., Nusbaum,C. and Birren,B.
EPI_ISL_2582880	Medicine, University of Washington, 300 9th Ave, Harborview Research & Training Building	Medicine, University of Washington, 300 9th Ave, Harborview Research & Training Building	Chu,H., Scott,E. and Roychoudhury,P.
EPI_ISL_2582883	Broad Institute of MIT & Harvard	Broad Institute of MIT & Harvard	Newman,R.M., Zody,M.C., DeVincenzo,J.P., Grad,Y., Lipsitch,M., Murphy,R., Fitzgerald,M., Young,S., Gargeya,S., Poon,T.W., Charlebois,P., Weiner,B., Yang,X., Piper,M.E., McCowan,C., Ireland,A., Levin,J., Malboeuf,C., Qu,J., Chapman,S.B., Murphy,C., Wortman,J., Nusbaum,C. and Birren,B.
EPI_ISL_2582886	J. Craig Venter Institute	J. Craig Venter Institute	Shabman,R., Fedorova,N., Puri,V., Shrivastava,S., Amedeo,P., Isom,R., Hu,L., Pickett,B., Novotny,M., Durbin,A., Rocchi,I., Williams,T., Hall,C.B., Tesini,B.L., Schnabel,K.C., Walsh,E.E. and Caserta,M.
EPI_ISL_2582892, EPI_ISL_2582893	Pediatrics - Infectious Diseases, Medical College of Wisconsin	Pediatrics - Infectious Diseases, Medical College of Wisconsin	Rebuffo-Scheer,C., Bose,M.E., He,J., Khaja,S., Ulatowski,M., Beck,E.T., Fan,J., Kumar,S., Nelson,M.I. and Henrickson,K.J.
EPI_ISL_2582901	J. Craig Venter Institute	J. Craig Venter Institute	Shabman,R., Fedorova,N., Puri,V., Shrivastava,S., Amedeo,P., Isom,R., Hu,L., Pickett,B., Novotny,M., Durbin,A., Rocchi,I., Williams,T., Hall,C.B., Tesini,B.L., Schnabel,K.C., Walsh,E.E. and Caserta,M.
EPI_ISL_2582904	Broad Institute of MIT & Harvard	Broad Institute of MIT & Harvard	Newman,R.M., Zody,M.C., DeVincenzo,J.P., Grad,Y., Lipsitch,M., Murphy,R., Fitzgerald,M., Young,S., Gargeya,S., Poon,T.W., Charlebois,P., Weiner,B., Yang,X., Piper,M.E., McCowan,C., Ireland,A., Levin,J., Malboeuf,C., Qu,J., Chapman,S.B., Murphy,C., Wortman,J., Nusbaum,C. and Birren,B.
EPI_ISL_2582906	Department of Pediatrics, Center of Excellence in Clinical Virology, Chulalongkorn	Department of Pediatrics, Center of Excellence in Clinical Virology, Chulalongkorn	Thongpan,I.
EPI_ISL_2582908, EPI_ISL_2582910	Epidemiology and Demography Department, KEMRI-Wellcome Trust Research Programme	Epidemiology and Demography Department, KEMRI-Wellcome Trust Research Programme	Otieno,J.R., Kamau,E.M., Oketch,J.W., Ngoi,J.M., Agoti,C.N., Gichuki,A.M., Otieno,G.P., Ngama,M., Cane,P.A., Kellam,P., Cotten,M., Lemey,P. and Nokes,D.J.
EPI_ISL_2582923, EPI_ISL_2582926, EPI_ISL_2582927, EPI_ISL_2582930, EPI_ISL_2582933	J. Craig Venter Institute	J. Craig Venter Institute	Shabman,R., Fedorova,N., Puri,V., Shrivastava,S., Amedeo,P., Isom,R., Hu,L., Pickett,B., Novotny,M., Durbin,A., Rocchi,I., Williams,T., Hall,C.B., Tesini,B.L., Schnabel,K.C., Walsh,E.E. and Caserta,M.
EPI_ISL_2582935, EPI_ISL_2582938, EPI_ISL_2582941	Broad Institute of MIT & Harvard	Broad Institute of MIT & Harvard	Newman,R.M., Zody,M.C., DeVincenzo,J.P., Grad,Y., Lipsitch,M., Murphy,R., Fitzgerald,M., Young,S., Gargeya,S., Poon,T.W., Charlebois,P., Weiner,B., Yang,X., Piper,M.E., McCowan,C., Ireland,A., Levin,J., Malboeuf,C., Qu,J., Chapman,S.B., Murphy,C., Wortman,J., Nusbaum,C. and Birren,B.
EPI_ISL_2582946, EPI_ISL_2582949	J. Craig Venter Institute	J. Craig Venter Institute	Shabman,R., Fedorova,N., Puri,V., Shrivastava,S., Amedeo,P., Isom,R., Hu,L., Pickett,B., Novotny,M., Durbin,A., Rocchi,I., Williams,T., Hall,C.B., Tesini,B.L., Schnabel,K.C., Walsh,E.E. and Caserta,M.
EPI_ISL_2582951	Broad Institute of MIT & Harvard	Broad Institute of MIT & Harvard	Newman,R.M., Zody,M.C., DeVincenzo,J.P., Grad,Y., Lipsitch,M., Murphy,R., Fitzgerald,M., Young,S., Gargeya,S., Poon,T.W., Charlebois,P., Weiner,B., Yang,X., Piper,M.E., McCowan,C., Ireland,A., Levin,J., Malboeuf,C., Qu,J., Chapman,S.B., Murphy,C., Wortman,J., Nusbaum,C. and Birren,B.
EPI_ISL_2582954	J. Craig Venter Institute	J. Craig Venter Institute	Shabman,R., Fedorova,N., Puri,V., Shrivastava,S., Amedeo,P., Isom,R., Hu,L., Pickett,B., Novotny,M., Durbin,A., Rocchi,I., Williams,T., Hall,C.B., Tesini,B.L., Schnabel,K.C., Walsh,E.E. and Caserta,M.
EPI_ISL_2582959	Epidemiology and Demography Department, KEMRI-Wellcome Trust Research Programme	Epidemiology and Demography Department, KEMRI-Wellcome Trust Research Programme	Otieno,J.R., Kamau,E.M., Oketch,J.W., Ngoi,J.M., Agoti,C.N., Gichuki,A.M., Otieno,G.P., Ngama,M., Cane,P.A., Kellam,P., Cotten,M., Lemey,P. and Nokes,D.J.
EPI_ISL_2582964, EPI_ISL_2582967	Pediatrics - Infectious Diseases, Medical College of Wisconsin	Pediatrics - Infectious Diseases, Medical College of Wisconsin	Rebuffo-Scheer,C., Bose,M.E., He,J., Khaja,S., Ulatowski,M., Beck,E.T., Fan,J., Kumar,S., Nelson,M.I. and Henrickson,K.J.
EPI_ISL_2582970	Epidemiology and Demography Department, KEMRI-Wellcome Trust Research Programme	Epidemiology and Demography Department, KEMRI-Wellcome Trust Research Programme	Otieno,J.R., Kamau,E.M., Oketch,J.W., Ngoi,J.M., Agoti,C.N., Gichuki,A.M., Otieno,G.P., Ngama,M., Cane,P.A., Kellam,P., Cotten,M., Lemey,P. and Nokes,D.J.
EPI_ISL_2582972, EPI_ISL_2582975	Pediatrics - Infectious Diseases, Medical College of Wisconsin	Pediatrics - Infectious Diseases, Medical College of Wisconsin	Rebuffo-Scheer,C., Bose,M.E., He,J., Khaja,S., Ulatowski,M., Beck,E.T., Fan,J., Kumar,S., Nelson,M.I. and Henrickson,K.J.
EPI_ISL_2582976	Infectious Disease Initiative, Broad Institute	Infectious Disease Initiative, Broad Institute	Newman,R.M., Zody,M.C., DeVincenzo,J.P., Grad,Y., Lipsitch,M., Murphy,R., Fitzgerald,M., Young,S., Gargeya,S., Poon,T.W., Charlebois,P., Weiner,B., Yang,X., Piper,M.E., McCowan,C., Ireland,A., Levin,J., Malboeuf,C., Qu,J., Chapman,S.B., Murphy,C., Wortman,J., Nusbaum,C. and Birren,B.
EPI_ISL_2582981	Pediatrics - Infectious Diseases, Medical College of Wisconsin	Pediatrics - Infectious Diseases, Medical College of Wisconsin	Rebuffo-Scheer,C., Bose,M.E., He,J., Khaja,S., Ulatowski,M., Beck,E.T., Fan,J., Kumar,S., Nelson,M.I. and Henrickson,K.J.
EPI_ISL_2582986, EPI_ISL_2582988, EPI_ISL_2582991, EPI_ISL_2582994, EPI_ISL_2582997, EPI_ISL_2583000, EPI_ISL_2583003, EPI_ISL_2583004	Broad Institute of MIT & Harvard	Broad Institute of MIT & Harvard	Newman,R.M., Zody,M.C., DeVincenzo,J.P., Grad,Y., Lipsitch,M., Murphy,R., Fitzgerald,M., Young,S., Gargeya,S., Poon,T.W., Charlebois,P., Weiner,B., Yang,X., Piper,M.E., McCowan,C., Ireland,A., Levin,J., Malboeuf,C., Qu,J., Chapman,S.B., Murphy,C., Wortman,J., Nusbaum,C. and Birren,B.
EPI_ISL_2583007	Pediatrics - Infectious Diseases, Medical College of Wisconsin	Pediatrics - Infectious Diseases, Medical College of Wisconsin	Rebuffo-Scheer,C., Bose,M.E., He,J., Khaja,S., Ulatowski,M., Beck,E.T., Fan,J., Kumar,S., Nelson,M.I. and Henrickson,K.J.
EPI_ISL_2583008, EPI_ISL_2583012	Broad Institute of MIT & Harvard	Broad Institute of MIT & Harvard	Newman,R.M., Zody,M.C., DeVincenzo,J.P., Grad,Y., Lipsitch,M., Murphy,R., Fitzgerald,M., Young,S., Gargeya,S., Poon,T.W., Charlebois,P., Weiner,B., Yang,X., Piper,M.E., McCowan,C., Ireland,A., Levin,J., Malboeuf,C., Qu,J., Chapman,S.B., Murphy,C., Wortman,J., Nusbaum,C. and Birren,B.
EPI_ISL_2583013	Pediatrics - Infectious Diseases, Medical College of Wisconsin	Pediatrics - Infectious Diseases, Medical College of Wisconsin	Rebuffo-Scheer,C., Bose,M.E., He,J., Khaja,S., Ulatowski,M., Beck,E.T., Fan,J., Kumar,S., Nelson,M.I. and Henrickson,K.J.
EPI_ISL_2583016, EPI_ISL_2583017, EPI_ISL_2583019, EPI_ISL_2583020, EPI_ISL_2583022	Broad Institute of MIT & Harvard	Broad Institute of MIT & Harvard	Newman,R.M., Zody,M.C., DeVincenzo,J.P., Grad,Y., Lipsitch,M., Murphy,R., Fitzgerald,M., Young,S., Gargeya,S., Poon,T.W., Charlebois,P., Weiner,B., Yang,X., Piper,M.E., McCowan,C., Ireland,A., Levin,J., Malboeuf,C., Qu,J., Chapman,S.B., Murphy,C., Wortman,J., Nusbaum,C. and Birren,B.
EPI_ISL_2583023, EPI_ISL_2583024	Epidemiology and Demography Department, KEMRI-Wellcome Trust Research Programme	Epidemiology and Demography Department, KEMRI-Wellcome Trust Research Programme	Otieno,J.R., Kamau,E.M., Oketch,J.W., Ngoi,J.M., Agoti,C.N., Gichuki,A.M., Otieno,G.P., Ngama,M., Cane,P.A., Kellam,P., Cotten,M., Lemey,P. and Nokes,D.J.
EPI_ISL_2583026	Medicine, University of Washington, 300 9th Ave, Harborview Research & Training Building	Medicine, University of Washington, 300 9th Ave, Harborview Research & Training Building	Chu,H., Scott,E. and Roychoudhury,P.
EPI_ISL_2583029	Broad Institute of MIT & Harvard	Broad Institute of MIT & Harvard	Newman,R.M., Zody,M.C., DeVincenzo,J.P., Grad,Y., Lipsitch,M., Murphy,R., Fitzgerald,M., Young,S., Gargeya,S., Poon,T.W., Charlebois,P., Weiner,B., Yang,X., Piper,M.E., McCowan,C., Ireland,A., Levin,J., Malboeuf,C., Qu,J., Chapman,S.B., Murphy,C., Wortman,J., Nusbaum,C. and Birren,B.
EPI_ISL_2583031	Pediatrics - Infectious Diseases, Medical College of Wisconsin	Pediatrics - Infectious Diseases, Medical College of Wisconsin	Rebuffo-Scheer,C., Bose,M.E., He,J., Khaja,S., Ulatowski,M., Beck,E.T., Fan,J., Kumar,S., Nelson,M.I. and Henrickson,K.J.
EPI_ISL_2583034	Broad Institute of MIT & Harvard	Broad Institute of MIT & Harvard	Newman,R.M., Zody,M.C., DeVincenzo,J.P., Grad,Y., Lipsitch,M., Murphy,R., Fitzgerald,M., Young,S., Gargeya,S., Poon,T.W., Charlebois,P., Weiner,B., Yang,X., Piper,M.E., McCowan,C., Ireland,A., Levin,J., Malboeuf,C., Qu,J., Chapman,S.B., Murphy,C., Wortman,J., Nusbaum,C. and Birren,B.

EPI_ISL_2583038	Pediatrics, University of New Mexico	Pediatrics, University of New Mexico	Kothari,A., Kennedy,J.L., Schwalm,K.C., Putt,C., Denson,J.L. and Dinwiddie,D.L.
EPI_ISL_2583040	Broad Institute of MIT & Harvard	Broad Institute of MIT & Harvard	Newman,R.M., Zody,M.C., DeVincenzo,J.P., Grad,Y., Lipsitch,M., Murphy,R., Fitzgerald,M., Young,S., Gargeya,S., Poon,T.W., Charlebois,P., Weiner,B., Yang,X., Piper,M.E., McCowan,C., Ireland,A., Levin,J., Malboeuf,C., Qu,J., Chapman,S.B., Murphy,C., Wortman,J., Nusbaum,C. and Birren,B.
EPI_ISL_2583042	Pediatrics, University of New Mexico	Pediatrics, University of New Mexico	Kothari,A., Kennedy,J.L., Schwalm,K.C., Putt,C., Denson,J.L. and Dinwiddie,D.L.
EPI_ISL_2583045, EPI_ISL_2583046, EPI_ISL_2583048, EPI_ISL_2583050, EPI_ISL_2583054, EPI_ISL_2583055	Broad Institute of MIT & Harvard	Broad Institute of MIT & Harvard	Newman,R.M., Zody,M.C., DeVincenzo,J.P., Grad,Y., Lipsitch,M., Murphy,R., Fitzgerald,M., Young,S., Gargeya,S., Poon,T.W., Charlebois,P., Weiner,B., Yang,X., Piper,M.E., McCowan,C., Ireland,A., Levin,J., Malboeuf,C., Qu,J., Chapman,S.B., Murphy,C., Wortman,J., Nusbaum,C. and Birren,B.
EPI_ISL_2583057	Pediatrics - Infectious Diseases, Medical College of Wisconsin	Pediatrics - Infectious Diseases, Medical College of Wisconsin	Rebuffo-Scheer,C., Bose,M.E., He,J., Khaja,S., Ulatowski,M., Beck,E.T., Fan,J., Kumar,S., Nelson,M.I. and Henrickson,K.J.
EPI_ISL_2583061, EPI_ISL_2583062, EPI_ISL_2583063	Pediatrics, University of New Mexico	Pediatrics, University of New Mexico	Kothari,A., Kennedy,J.L., Schwalm,K.C., Putt,C., Denson,J.L. and Dinwiddie,D.L.
EPI_ISL_2583065	Pediatrics - Infectious Diseases, Medical College of Wisconsin	Pediatrics - Infectious Diseases, Medical College of Wisconsin	Rebuffo-Scheer,C., Bose,M.E., He,J., Khaja,S., Ulatowski,M., Beck,E.T., Fan,J., Kumar,S., Nelson,M.I. and Henrickson,K.J.
EPI_ISL_2583068, EPI_ISL_2583072, EPI_ISL_2583074	Broad Institute of MIT & Harvard	Broad Institute of MIT & Harvard	Newman,R.M., Zody,M.C., DeVincenzo,J.P., Grad,Y., Lipsitch,M., Murphy,R., Fitzgerald,M., Young,S., Gargeya,S., Poon,T.W., Charlebois,P., Weiner,B., Yang,X., Piper,M.E., McCowan,C., Ireland,A., Levin,J., Malboeuf,C., Qu,J., Chapman,S.B., Murphy,C., Wortman,J., Nusbaum,C. and Birren,B.
EPI_ISL_2583076	Pediatrics, University of New Mexico	Pediatrics, University of New Mexico	Kothari,A., Kennedy,J.L., Schwalm,K.C., Putt,C., Denson,J.L. and Dinwiddie,D.L.
EPI_ISL_2583078, EPI_ISL_2583080, EPI_ISL_2583082	Broad Institute of MIT & Harvard	Broad Institute of MIT & Harvard	Newman,R.M., Zody,M.C., DeVincenzo,J.P., Grad,Y., Lipsitch,M., Murphy,R., Fitzgerald,M., Young,S., Gargeya,S., Poon,T.W., Charlebois,P., Weiner,B., Yang,X., Piper,M.E., McCowan,C., Ireland,A., Levin,J., Malboeuf,C., Qu,J., Chapman,S.B., Murphy,C., Wortman,J., Nusbaum,C. and Birren,B.
EPI_ISL_2583083	Pediatrics - Infectious Diseases, Medical College of Wisconsin	Pediatrics - Infectious Diseases, Medical College of Wisconsin	Rebuffo-Scheer,C., Bose,M.E., He,J., Khaja,S., Ulatowski,M., Beck,E.T., Fan,J., Kumar,S., Nelson,M.I. and Henrickson,K.J.
EPI_ISL_2583085, EPI_ISL_2583087	Broad Institute of MIT & Harvard	Broad Institute of MIT & Harvard	Newman,R.M., Zody,M.C., DeVincenzo,J.P., Grad,Y., Lipsitch,M., Murphy,R., Fitzgerald,M., Young,S., Gargeya,S., Poon,T.W., Charlebois,P., Weiner,B., Yang,X., Piper,M.E., McCowan,C., Ireland,A., Levin,J., Malboeuf,C., Qu,J., Chapman,S.B., Murphy,C., Wortman,J., Nusbaum,C. and Birren,B.
EPI_ISL_2583091	Epidemiology and Demography Department, KEMRI-Wellcome Trust Research Programme	Epidemiology and Demography Department, KEMRI-Wellcome Trust Research Programme	Otieno,J.R., Kamau,E.M., Oketch,J.W., Ngoi,J.M., Agoti,C.N., Gichuki,A.M., Otieno,G.P., Ngama,M., Cane,P.A., Kellam,P., Cotten,M., Lemey,P. and Nokes,D.J.
EPI_ISL_2583092	Broad Institute of MIT & Harvard	Broad Institute of MIT & Harvard	Newman,R.M., Zody,M.C., DeVincenzo,J.P., Grad,Y., Lipsitch,M., Murphy,R., Fitzgerald,M., Young,S., Gargeya,S., Poon,T.W., Charlebois,P., Weiner,B., Yang,X., Piper,M.E., McCowan,C., Ireland,A., Levin,J., Malboeuf,C., Qu,J., Chapman,S.B., Murphy,C., Wortman,J., Nusbaum,C. and Birren,B.
EPI_ISL_2584483, EPI_ISL_2584484, EPI_ISL_2584485, EPI_ISL_2584486	J. Craig Venter Institute	J. Craig Venter Institute	Shabman,R., Das,S.R., Puri,V., Fedorova,N., Amedeo,P., Williams,M., Shrivastava,S. and Halasa,N.
EPI_ISL_2584489, EPI_ISL_2584491, EPI_ISL_2584492, EPI_ISL_2584494, EPI_ISL_2584496, EPI_ISL_2584497, EPI_ISL_2584498, EPI_ISL_2584499, EPI_ISL_2584500, EPI_ISL_2584504	Epidemiology and Demography, KEMRI-Wellcome Trust	Epidemiology and Demography, KEMRI-Wellcome Trust	Kamau,E., Otieno,J.R., Murunga,N., Nyiro,J.U., Oketch,J.W., Ngoi,J.M., de Laurent,Z.R., Mwema,A., Agoti,C.N. and Nokes,D.J.
EPI_ISL_2584505	Medical Microbiology, University Medical Center Utrecht	Medical Microbiology, University Medical Center Utrecht	Tan,L., Viveen,M.C., Lemey,P. and Coenjaerts,F.E.
EPI_ISL_2584506	Medical Microbiology, University Medical Center Utrecht	Medical Microbiology, University Medical Center Utrecht	Tan,L., Lemey,P., Viveen,M. and Coenjaerts,F.
EPI_ISL_2584511, EPI_ISL_2584512, EPI_ISL_2584513, EPI_ISL_2584514, EPI_ISL_2584515, EPI_ISL_2584516	J. Craig Venter Institute	J. Craig Venter Institute	Shabman,R., Das,S.R., Shilts,M., Fedorova,N., Puri,V., Shrivastava,S., Amedeo,P., Williams,M., Barratt,K., Mitchell,J. and Jennings,L.
EPI_ISL_2584518	J. Craig Venter Institute	J. Craig Venter Institute	Wentworth,D.E., Halpin,R.A., Bera,J., Lin,X., Fedorova,N., Tsitrin,T., McLellan,M., Stockwell,T., Amedeo,P., Bishop,B., Gupta,N., Hoover,J., Katzel,D., Schobel,S., Shrivastava,S., Garcia,J., Laguna-Torres,V.A., Leguia,M., Benavides,J.G. and Halsey,E.
EPI_ISL_2584558, EPI_ISL_2584560, EPI_ISL_2584562, EPI_ISL_2584565, EPI_ISL_2584566, EPI_ISL_2584568, EPI_ISL_2584569, EPI_ISL_2584570	J. Craig Venter Institute	J. Craig Venter Institute	Das,S.R., Halpin,R.A., Shilts,M., Puri,V., Akopov,A., Fedorova,N., Stockwell,T., Amedeo,P., Bishop,B., Katzel,D., Schobel,S., Shrivastava,S. and Hartert,T.
EPI_ISL_2584571, EPI_ISL_2584572	J. Craig Venter Institute	J. Craig Venter Institute	Wentworth,D.E., Halpin,R.A., Bera,J., Lin,X., Fedorova,N., Tsitrin,T., McLellan,M., Stockwell,T., Amedeo,P., Bishop,B., Gupta,N., Hoover,J., Katzel,D., Schobel,S., Shrivastava,S., Garcia,J., Laguna-Torres,V.A., Leguia,M., Benavides,J.G. and Halsey,E.
EPI_ISL_2584579, EPI_ISL_2584582	Medicine, University of Washington, 300 9th Ave, Harborview Research & Training Building	Medicine, University of Washington, 300 9th Ave, Harborview Research & Training Building	Chu,H., Scott,E. and Roychoudhury,P.
EPI_ISL_2584583	J. Craig Venter Institute	J. Craig Venter Institute	Shabman,R., Das,S.R., Puri,V., Fedorova,N., Amedeo,P., Williams,M., Shrivastava,S. and Halasa,N.
EPI_ISL_2584584, EPI_ISL_2584585, EPI_ISL_2584586, EPI_ISL_2584587	J. Craig Venter Institute	J. Craig Venter Institute	Das,S.R., Halpin,R.A., Shilts,M., Puri,V., Akopov,A., Fedorova,N., Stockwell,T., Amedeo,P., Bishop,B., Katzel,D., Schobel,S., Shrivastava,S. and Hartert,T.
EPI_ISL_2584588	J. Craig Venter Institute	J. Craig Venter Institute	Das,S., Halpin,R.A., Bera,J., Puri,V., Fedorova,N., Tsitrin,T., Stockwell,T., Amedeo,P., Bishop,B., Katzel,D., Schobel,S., Shrivastava,S., Hartert,T., Moore,M., Chappell,J., Larkin,E., Wentworth,D.E. and Anderson,L.J.
EPI_ISL_2584590	J. Craig Venter Institute	J. Craig Venter Institute	Das,S.R., Halpin,R.A., Puri,V., Akopov,A., Fedorova,N., Stockwell,T., Amedeo,P., Bishop,B., Katzel,D., Schobel,S., Shrivastava,S., Hall,C.B., Tesini,B.L., Schnabel,K.C., Walsh,E.E. and Caserta,M.
EPI_ISL_2584591, EPI_ISL_2584592, EPI_ISL_2584593, EPI_ISL_2584594	Epidemiology and Demography, KEMRI-Wellcome Trust	Epidemiology and Demography, KEMRI-Wellcome Trust	Kamau,E., Otieno,J.R., Murunga,N., Nyiro,J.U., Oketch,J.W., Ngoi,J.M., de Laurent,Z.R., Mwema,A., Agoti,C.N. and Nokes,D.J.
EPI_ISL_2584595	J. Craig Venter Institute	J. Craig Venter Institute	Das,S.R., Halpin,R.A., Shilts,M., Puri,V., Akopov,A., Fedorova,N., Stockwell,T., Amedeo,P., Bishop,B., Katzel,D., Schobel,S., Shrivastava,S. and Hartert,T.
EPI_ISL_2584596	J. Craig Venter Institute	J. Craig Venter Institute	Das,S.R., Halpin,R.A., Puri,V., Akopov,A., Fedorova,N., Stockwell,T., Amedeo,P., Bishop,B., Katzel,D., Schobel,S., Shrivastava,S., Hall,C.B., Tesini,B.L., Schnabel,K.C., Walsh,E.E. and Caserta,M.
EPI_ISL_2584597	Pediatrics, UT Southwestern Medical Center	Pediatrics, UT Southwestern Medical Center	Levitz,R., Gao,Y., Dozmorov,I., Song,R., Wakeland,E.K. and Kahn,J.S.
EPI_ISL_2584598	Medical Microbiology, University Medical Center Utrecht	Medical Microbiology, University Medical Center Utrecht	Tan,L., Lemey,P., Viveen,M. and Coenjaerts,F.E.J.
EPI_ISL_2584599, EPI_ISL_2584600	J. Craig Venter Institute	J. Craig Venter Institute	Das,S.R., Halpin,R.A., Puri,V., Akopov,A., Fedorova,N., Stockwell,T., Amedeo,P., Bishop,B., Katzel,D., Schobel,S., Shrivastava,S., Hall,C.B., Tesini,B.L., Schnabel,K.C., Walsh,E.E. and Caserta,M.
EPI_ISL_2584601	J. Craig Venter Institute	J. Craig Venter Institute	Das,S.R., Halpin,R.A., Puri,V., Akopov,A., Fedorova,N., Stockwell,T., Amedeo,P., Bishop,B., Katzel,D., Schobel,S., Shrivastava,S., Wentworth,D.E. and Caserta,M.
EPI_ISL_2584602	Medical Microbiology, University Medical Center Utrecht	Medical Microbiology, University Medical Center Utrecht	Tan,L., Lemey,P., Viveen,M. and Coenjaerts,F.E.J.
EPI_ISL_2584604	J. Craig Venter Institute	J. Craig Venter Institute	Das,S.R., Halpin,R.A., Puri,V., Akopov,A., Fedorova,N., Tsitrin,T., Stockwell,T., Amedeo,P., Bishop,B., Gupta,N., Hoover,J., Katzel,D., Schobel,S., Shrivastava,S., Wentworth,D.E. and Caserta,M.
EPI_ISL_2584605, EPI_ISL_2584606, EPI_ISL_2584610, EPI_ISL_2584611, EPI_ISL_2584613, EPI_ISL_2584614	J. Craig Venter Institute	J. Craig Venter Institute	Das,S.R., Halpin,R.A., Puri,V., Akopov,A., Fedorova,N., Stockwell,T., Amedeo,P., Bishop,B., Katzel,D., Schobel,S., Shrivastava,S., Hall,C.B., Tesini,B.L., Schnabel,K.C., Walsh,E.E. and Caserta,M.

EPI_ISL_2584615	J. Craig Venter Institute	J. Craig Venter Institute	Das,S.R., Halpin,R.A., Puri,V., Akopov,A., Fedorova,N., Stockwell,T., Amedeo,P., Bishop,B., Katzel,D., Schobel,S., Shrivastava,S., Wentworth,D.E. and Caserta,M.
EPI_ISL_2584617	Pediatrics and Microbiology, University of Texas Southwestern Medical Center	Pediatrics and Microbiology, University of Texas Southwestern Medical Center	Levitz,R., Wattier,R., Phillips,P., Solomon,A., Lawler,J., Lazar,I. and Kahn,J.S.
EPI_ISL_2584618	J. Craig Venter Institute	J. Craig Venter Institute	Das,S.R., Halpin,R.A., Puri,V., Akopov,A., Fedorova,N., Stockwell,T., Amedeo,P., Bishop,B., Katzel,D., Schobel,S., Shrivastava,S., Wentworth,D.E. and Caserta,M.
EPI_ISL_2584619	J. Craig Venter Institute	J. Craig Venter Institute	Das,S.R., Halpin,R.A., Puri,V., Akopov,A., Fedorova,N., Stockwell,T., Amedeo,P., Bishop,B., Katzel,D., Schobel,S., Shrivastava,S., Hall,C.B., Tesini,B.L., Schnabel,K.C., Walsh,E.E. and Caserta,M.
EPI_ISL_2584620	J. Craig Venter Institute	J. Craig Venter Institute	Das,S.R., Halpin,R.A., Puri,V., Akopov,A., Fedorova,N., Stockwell,T., Amedeo,P., Bishop,B., Katzel,D., Schobel,S., Shrivastava,S., Wentworth,D.E. and Caserta,M.
EPI_ISL_2584621	Biologia Molecular y Validacion de Tecnicas, Instituto de Diagnostico y Referencia Epidemiologicos (InDRE)	Biologia Molecular y Validacion de Tecnicas, Instituto de Diagnostico y Referencia Epidemiologicos (InDRE)	Ortiz-Alcantara,J.M., Garces-Ayala,F., Perez-Agueros,S.I., Hernandez-Moreno,A.L., Munoz-Medina,J.E., Monroy-Munoz,I.E., Santos Coy-Arechavaleta,A., Meza-Chavez,A., Angeles-Martinez,J., Anguiano-Hernandez,Y.-M., Martinez-Miguel,B., Santacruz-Tinoco,C.E., Gonzalez-Ibarra,J., Alvarado-Yaah,J.E., Gonzalez-Bonilla,C.R., Diaz-Quinonez,J.A. and Ramirez-Gonzalez,J.E.
EPI_ISL_2584622	Medical Microbiology, University Medical Center Utrecht	Medical Microbiology, University Medical Center Utrecht	Tan,L., Viveen,M.C., Lemey,P. and Coenjaerts,F.E.
EPI_ISL_2584623	J. Craig Venter Institute	J. Craig Venter Institute	Wentworth,D.E., Halpin,R.A., Bera,J., Lin,X., Fedorova,N., Tsitrin,T., McLellan,M., Stockwell,T., Amedeo,P., Bishop,B., Gupta,N., Hoover,J., Katzel,D., Schobel,S., Shrivastava,S., Garcia,J., Laguna-Torres,V.A., Leguia,M., Benavides,J.G. and Halsey,E.
EPI_ISL_2584624	Medical Microbiology, University Medical Center Utrecht	Medical Microbiology, University Medical Center Utrecht	Tan,L., Viveen,M.C., Lemey,P. and Coenjaerts,F.E.
EPI_ISL_2584626	KEMRI Wellcome Trust Research Programme	KEMRI Wellcome Trust Research Programme	Agoti,C.N., Otieno,J.R., Munywoki,P.K., Mwihuri,A.G., Cane,P.A., Nokes,D.J., Kellam,P. and Cotten,M.L.
EPI_ISL_2584627	Virology, Public Health Institution of Turkey	Virology, Public Health Institution of Turkey	Bayrakdar,F.
EPI_ISL_2584628	J. Craig Venter Institute	J. Craig Venter Institute	Shabman,R., Das,S.R., Shilts,M., Fedorova,N., Puri,V., Shrivastava,S., Amedeo,P., Williams,M., Barratt,K., Mitchell,J. and Jennings,L.
EPI_ISL_2584630	J. Craig Venter Institute	J. Craig Venter Institute	Wentworth,D.E., Halpin,R.A., Bera,J., Lin,X., Fedorova,N., Tsitrin,T., McLellan,M., Stockwell,T., Amedeo,P., Bishop,B., Gupta,N., Hoover,J., Katzel,D., Schobel,S., Shrivastava,S., Garcia,J., Laguna-Torres,V.A., Leguia,M., Benavides,J.G. and Halsey,E.
EPI_ISL_2584631, EPI_ISL_2584632	Medical Microbiology, University Medical Center Utrecht	Medical Microbiology, University Medical Center Utrecht	Tan,L., Viveen,M.C., Lemey,P. and Coenjaerts,F.E.
EPI_ISL_2584633	J. Craig Venter Institute	J. Craig Venter Institute	Shabman,R., Das,S.R., Shilts,M., Fedorova,N., Puri,V., Shrivastava,S., Amedeo,P., Williams,M., Barratt,K., Mitchell,J. and Jennings,L.
EPI_ISL_2584634	Medical Microbiology, University Medical Center Utrecht	Medical Microbiology, University Medical Center Utrecht	Tan,L., Lemey,P., Viveen,M. and Coenjaerts,F.E.J.
EPI_ISL_2584635	J. Craig Venter Institute	J. Craig Venter Institute	Das,S.R., Halpin,R.A., Shilts,M., Puri,V., Akopov,A., Fedorova,N., Stockwell,T., Amedeo,P., Bishop,B., Katzel,D., Schobel,S., Shrivastava,S. and Hartert,T.
EPI_ISL_2584636, EPI_ISL_2584637	J. Craig Venter Institute	J. Craig Venter Institute	Shabman,R., Das,S.R., Shilts,M., Fedorova,N., Puri,V., Shrivastava,S., Amedeo,P., Williams,M., Barratt,K., Mitchell,J. and Jennings,L.
EPI_ISL_2584638	J. Craig Venter Institute	J. Craig Venter Institute	Shabman,R., Das,S.R., Puri,V., Fedorova,N., Amedeo,P., Williams,M., Shrivastava,S. and Halasa,N.
EPI_ISL_2584639	Biologia Molecular y Validacion de Tecnicas, Instituto de Diagnostico y Referencia Epidemiologicos (InDRE)	Biologia Molecular y Validacion de Tecnicas, Instituto de Diagnostico y Referencia Epidemiologicos (InDRE)	Munoz-Medina,J.E., Monroy-Munoz,I.E., Santos Coy-Arechavaleta,A., Meza-Chavez,A., Angeles-Martinez,J., Anguiano-Hernandez,Y.M., Santacruz-Tinoco,C.E., Gonzalez-Ibarra,J., Martinez-Miguel,B., Alvarado-Yaah,J.E., Palomec-Nava,I.D., Ortiz-Alcantara,J.M., Garces-Ayala,F., Ramirez-Gonzalez,J.E., Diaz-Quinonez,J.A. and Gonzalez-Bonilla,C.R.
EPI_ISL_2584640, EPI_ISL_2584641	J. Craig Venter Institute	J. Craig Venter Institute	Shabman,R., Das,S.R., Puri,V., Fedorova,N., Amedeo,P., Williams,M., Shrivastava,S. and Halasa,N.
EPI_ISL_2584642	Medical Microbiology, University Medical Center Utrecht	Medical Microbiology, University Medical Center Utrecht	Tan,L., Viveen,M.C., Lemey,P. and Coenjaerts,F.E.
EPI_ISL_2584644, EPI_ISL_2584646, EPI_ISL_2584648, EPI_ISL_2584650	J. Craig Venter Institute	J. Craig Venter Institute	Das,S.R., Halpin,R.A., Shilts,M., Puri,V., Akopov,A., Fedorova,N., Stockwell,T., Amedeo,P., Bishop,B., Katzel,D., Schobel,S., Shrivastava,S. and Hartert,T.
EPI_ISL_2584652	J. Craig Venter Institute	J. Craig Venter Institute	Das,S.R., Halpin,R.A., Puri,V., Akopov,A., Fedorova,N., Tsitrin,T., Stockwell,T., Amedeo,P., Bishop,B., Gupta,N., Hoover,J., Katzel,D., Schobel,S., Shrivastava,S., Wentworth,D.E. and Caserta,M.
EPI_ISL_2584653, EPI_ISL_2584654	Epidemiology and Demography, KEMRI-Wellcome Trust	Epidemiology and Demography, KEMRI-Wellcome Trust	Kamau,E., Otieno,J.R., Murunga,N., Nyiro,J.U., Oketch,J.W., Ngoi,J.M., de Laurent,Z.R., Mwema,A., Agoti,C.N. and Nokes,D.J.
EPI_ISL_2584656	J. Craig Venter Institute	J. Craig Venter Institute	Shabman,R., Das,S.R., Shilts,M., Fedorova,N., Puri,V., Shrivastava,S., Amedeo,P., Williams,M., Barratt,K., Mitchell,J. and Jennings,L.
EPI_ISL_2584657	Epidemiology and Demography, KEMRI-Wellcome Trust	Epidemiology and Demography, KEMRI-Wellcome Trust	Kamau,E., Otieno,J.R., Murunga,N., Nyiro,J.U., Oketch,J.W., Ngoi,J.M., de Laurent,Z.R., Mwema,A., Agoti,C.N. and Nokes,D.J.
EPI_ISL_2584658	J. Craig Venter Institute	J. Craig Venter Institute	Shabman,R., Das,S.R., Shilts,M., Fedorova,N., Puri,V., Shrivastava,S., Amedeo,P., Williams,M., Barratt,K., Mitchell,J. and Jennings,L.
EPI_ISL_2584659, EPI_ISL_2584660	J. Craig Venter Institute	J. Craig Venter Institute	Wentworth,D.E., Halpin,R.A., Bera,J., Lin,X., Fedorova,N., Tsitrin,T., McLellan,M., Stockwell,T., Amedeo,P., Bishop,B., Gupta,N., Hoover,J., Katzel,D., Schobel,S., Shrivastava,S., Garcia,J., Laguna-Torres,V.A., Leguia,M., Benavides,J.G. and Halsey,E.
EPI_ISL_2584661	Epidemiology and Demography, KEMRI-Wellcome Trust	Epidemiology and Demography, KEMRI-Wellcome Trust	Kamau,E., Otieno,J.R., Murunga,N., Nyiro,J.U., Oketch,J.W., Ngoi,J.M., de Laurent,Z.R., Mwema,A., Agoti,C.N. and Nokes,D.J.
EPI_ISL_2584662	J. Craig Venter Institute	J. Craig Venter Institute	Shabman,R., Das,S.R., Shilts,M., Fedorova,N., Puri,V., Shrivastava,S., Amedeo,P., Williams,M., Barratt,K., Mitchell,J. and Jennings,L.
EPI_ISL_2584664, EPI_ISL_2584665, EPI_ISL_2584666, EPI_ISL_2584667, EPI_ISL_2584668, EPI_ISL_2584669, EPI_ISL_2584670	Epidemiology and Demography, KEMRI-Wellcome Trust	Epidemiology and Demography, KEMRI-Wellcome Trust	Kamau,E., Otieno,J.R., Murunga,N., Nyiro,J.U., Oketch,J.W., Ngoi,J.M., de Laurent,Z.R., Mwema,A., Agoti,C.N. and Nokes,D.J.
EPI_ISL_2584671	J. Craig Venter Institute	J. Craig Venter Institute	Shabman,R., Das,S.R., Shilts,M., Fedorova,N., Puri,V., Shrivastava,S., Amedeo,P., Williams,M., Barratt,K., Mitchell,J. and Jennings,L.
EPI_ISL_2584673, EPI_ISL_2584674	Epidemiology and Demography, KEMRI-Wellcome Trust	Epidemiology and Demography, KEMRI-Wellcome Trust	Kamau,E., Otieno,J.R., Murunga,N., Nyiro,J.U., Oketch,J.W., Ngoi,J.M., de Laurent,Z.R., Mwema,A., Agoti,C.N. and Nokes,D.J.
EPI_ISL_2584675	Medical Microbiology, University Medical Center Utrecht	Medical Microbiology, University Medical Center Utrecht	Tan,L., Lemey,P., Viveen,M. and Coenjaerts,F.
EPI_ISL_2584676, EPI_ISL_2584677	Epidemiology and Demography, KEMRI-Wellcome Trust	Epidemiology and Demography, KEMRI-Wellcome Trust	Kamau,E., Otieno,J.R., Murunga,N., Nyiro,J.U., Oketch,J.W., Ngoi,J.M., de Laurent,Z.R., Mwema,A., Agoti,C.N. and Nokes,D.J.
EPI_ISL_2584678	J. Craig Venter Institute	J. Craig Venter Institute	Das,S.R., Halpin,R.A., Puri,V., Akopov,A., Fedorova,N., Stockwell,T., Amedeo,P., Bishop,B., Katzel,D., Schobel,S., Shrivastava,S., Wentworth,D.E. and Caserta,M.
EPI_ISL_2584680	Epidemiology and Demography, KEMRI-Wellcome Trust	Epidemiology and Demography, KEMRI-Wellcome Trust	Kamau,E., Otieno,J.R., Murunga,N., Nyiro,J.U., Oketch,J.W., Ngoi,J.M., de Laurent,Z.R., Mwema,A., Agoti,C.N. and Nokes,D.J.
EPI_ISL_2584681	J. Craig Venter Institute	J. Craig Venter Institute	Das,S.R., Halpin,R.A., Puri,V., Akopov,A., Fedorova,N., Stockwell,T., Amedeo,P., Bishop,B., Katzel,D., Schobel,S., Shrivastava,S., Hall,C.B., Tesini,B.L., Schnabel,K.C., Walsh,E.E. and Caserta,M.
EPI_ISL_2584682	J. Craig Venter Institute	J. Craig Venter Institute	Das,S.R., Halpin,R.A., Puri,V., Akopov,A., Fedorova,N., Tsitrin,T., Stockwell,T., Amedeo,P., Bishop,B., Gupta,N., Hoover,J., Katzel,D., Schobel,S., Shrivastava,S., Wentworth,D.E. and Caserta,M.
EPI_ISL_2584683, EPI_ISL_2584684, EPI_ISL_2584685	J. Craig Venter Institute	J. Craig Venter Institute	Das,S.R., Halpin,R.A., Puri,V., Akopov,A., Fedorova,N., Stockwell,T., Amedeo,P., Bishop,B., Katzel,D., Schobel,S., Shrivastava,S., Hall,C.B., Tesini,B.L., Schnabel,K.C., Walsh,E.E. and Caserta,M.
EPI_ISL_2584686	J. Craig Venter Institute	J. Craig Venter Institute	Shabman,R., Das,S.R., Puri,V., Fedorova,N., Amedeo,P., Williams,M., Shrivastava,S. and Halasa,N.
EPI_ISL_2584688, EPI_ISL_2584690, EPI_ISL_2584691	Epidemiology and Demography, KEMRI-Wellcome Trust	Epidemiology and Demography, KEMRI-Wellcome Trust	Kamau,E., Otieno,J.R., Murunga,N., Nyiro,J.U., Oketch,J.W., Ngoi,J.M., de Laurent,Z.R., Mwema,A., Agoti,C.N. and Nokes,D.J.
EPI_ISL_2584692	Medicine, University of Washington, 300 9th Ave, Harborview	Medicine, University of Washington, 300 9th Ave, Harborview	Chu,H., Scott,E. and Roychoudhury,P.

	Research & Training Building	Research & Training Building	
EPI_ISL_2584693	Epidemiology and Demography, KEMRI-Wellcome Trust	Epidemiology and Demography, KEMRI-Wellcome Trust	Kamau,E., Otieno,J.R., Murunga,N., Nyiro,J.U., Oketch,J.W., Ngoi,J.M., de Laurent,Z.R., Mwema,A., Agoti,C.N. and Nokes,D.J.
EPI_ISL_2584696	J. Craig Venter Institute	J. Craig Venter Institute	Das,S.R., Halpin,R.A., Puri,V., Akopov,A., Fedorova,N., Tsitrin,T., Stockwell,T., Amedeo,P., Bishop,B., Gupta,N., Hoover,J., Katzel,D., Schobel,S., Shrivastava,S., Wentworth,D.E. and Caserta,M.
EPI_ISL_2584697	J. Craig Venter Institute	J. Craig Venter Institute	Das,S.R., Halpin,R.A., Puri,V., Akopov,A., Fedorova,N., Stockwell,T., Amedeo,P., Bishop,B., Katzel,D., Schobel,S., Shrivastava,S., Wentworth,D.E. and Caserta,M.
EPI_ISL_2584698	J. Craig Venter Institute	J. Craig Venter Institute	Das,S.R., Halpin,R.A., Puri,V., Akopov,A., Fedorova,N., Stockwell,T., Amedeo,P., Bishop,B., Katzel,D., Schobel,S., Shrivastava,S., Hall,C.B., Tesini,B.L., Schnabel,K.C., Walsh,E.E. and Caserta,M.
EPI_ISL_2584699	Epidemiology and Demography, KEMRI-Wellcome Trust	Epidemiology and Demography, KEMRI-Wellcome Trust	Kamau,E., Otieno,J.R., Murunga,N., Nyiro,J.U., Oketch,J.W., Ngoi,J.M., de Laurent,Z.R., Mwema,A., Agoti,C.N. and Nokes,D.J.
EPI_ISL_2584703, EPI_ISL_2584705	J. Craig Venter Institute	J. Craig Venter Institute	Das,S.R., Halpin,R.A., Puri,V., Akopov,A., Fedorova,N., Stockwell,T., Amedeo,P., Bishop,B., Katzel,D., Schobel,S., Shrivastava,S., Hall,C.B., Tesini,B.L., Schnabel,K.C., Walsh,E.E. and Caserta,M.
EPI_ISL_2584706, EPI_ISL_2584708, EPI_ISL_2584711, EPI_ISL_2584715, EPI_ISL_2584717, EPI_ISL_2584718, EPI_ISL_2584719, EPI_ISL_2584720, EPI_ISL_2584722, EPI_ISL_2584723, EPI_ISL_2584724, EPI_ISL_2584725			
see above	Epidemiology and Demography, KEMRI-Wellcome Trust	Epidemiology and Demography, KEMRI-Wellcome Trust	Kamau,E., Otieno,J.R., Murunga,N., Nyiro,J.U., Oketch,J.W., Ngoi,J.M., de Laurent,Z.R., Mwema,A., Agoti,C.N. and Nokes,D.J.
EPI_ISL_2584726	KEMRI Wellcome Trust Research Programme	KEMRI Wellcome Trust Research Programme	Agoti,C.N., Otieno,J.R., Munywoki,P.K., Mwihuri,A.G., Cane,P.A., Nokes,D.J., Kellam,P. and Cotten,M.L.
EPI_ISL_2584727	J. Craig Venter Institute	J. Craig Venter Institute	Das,S., Halpin,R.A., Bera,J., Fedorova,N., Tsitrin,T., Stockwell,T., Amedeo,P., Bishop,B., Gupta,N., Hoover,J., Katzel,D., Schobel,S., Shrivastava,S., Hartert,T., Moore,M., Chappell,J., Larkin,E., Wentworth,D.E. and Anderson,L.J.
EPI_ISL_2584728	J. Craig Venter Institute	J. Craig Venter Institute	Wentworth,D.E., Halpin,R.A., Bera,J., Lin,X., Fedorova,N., Tsitrin,T., McLellan,M., Stockwell,T., Amedeo,P., Bishop,B., Gupta,N., Hoover,J., Katzel,D., Schobel,S., Shrivastava,S., Garcia,J., Laguna-Torres,V.A., Leguia,M., Benavides,J.G. and Halsey,E.
EPI_ISL_2584729	J. Craig Venter Institute	J. Craig Venter Institute	Das,S.R., Halpin,R.A., Shilts,M., Puri,V., Akopov,A., Fedorova,N., Stockwell,T., Amedeo,P., Bishop,B., Katzel,D., Schobel,S., Shrivastava,S. and Hartert,T.
EPI_ISL_2584730, EPI_ISL_2584731	Medical Microbiology, University Medical Center Utrecht	Medical Microbiology, University Medical Center Utrecht	Tan,L., Viveen,M.C., Lemey,P. and Coenjaerts,F.E.
EPI_ISL_2584732	J. Craig Venter Institute	J. Craig Venter Institute	Shabman,R., Das,S.R., Shilts,M., Fedorova,N., Puri,V., Shrivastava,S., Amedeo,P., Williams,M., Barratt,K., Mitchell,J. and Jennings,L.
EPI_ISL_2584733	J. Craig Venter Institute	J. Craig Venter Institute	Shabman,R., Das,S.R., Shilts,M., Fedorova,N., Puri,V., Shrivastava,S., Amedeo,P., Hu,L., Durbin,A., Rocchi,I., Williams,T. and Hartert,T.
EPI_ISL_2584734	J. Craig Venter Institute	J. Craig Venter Institute	Shabman,R., Das,S.R., Puri,V., Fedorova,N., Amedeo,P., Williams,M., Shrivastava,S. and Halasa,N.
EPI_ISL_2584735, EPI_ISL_2584736	J. Craig Venter Institute	J. Craig Venter Institute	Das,S.R., Halpin,R.A., Shilts,M., Puri,V., Akopov,A., Fedorova,N., Stockwell,T., Amedeo,P., Bishop,B., Katzel,D., Schobel,S., Shrivastava,S. and Hartert,T.
EPI_ISL_2584737	J. Craig Venter Institute	J. Craig Venter Institute	Shabman,R., Das,S.R., Shilts,M., Fedorova,N., Puri,V., Shrivastava,S., Amedeo,P., Williams,M., Barratt,K., Mitchell,J. and Jennings,L.
EPI_ISL_2584738	Laboratory Medicine, UW Virology	Laboratory Medicine, UW Virology	Lin,M.J., Tait,A. and Greninger,A.L.
EPI_ISL_2584739	J. Craig Venter Institute	J. Craig Venter Institute	Das,S.R., Halpin,R.A., Shilts,M., Puri,V., Akopov,A., Fedorova,N., Stockwell,T., Amedeo,P., Bishop,B., Katzel,D., Schobel,S., Shrivastava,S. and Hartert,T.
EPI_ISL_2584740	Medical Microbiology, University Medical Center Utrecht	Medical Microbiology, University Medical Center Utrecht	Tan,L., Viveen,M.C., Lemey,P. and Coenjaerts,F.E.
EPI_ISL_2584741	J. Craig Venter Institute	J. Craig Venter Institute	Lorenzi,H., Town,C., Halpin,R., Bera,J., Ransier,A., Fedorova,N., Stockwell,T., Amedeo,P., Appalla,L., Bishop,B., Edworthy,P., Gupta,N., Hoover,J., Katzel,D., Li,K., Schobel,S., Shrivastava,S., Thovarai,V., Wang,S., Rebuffo-Scheer,C., Fan,J., He,J., Kehl,S.C., Lederboer,N., Jurgens,L.A., Bose,M.E., Beck,E.T., Kumar,S., Wentworth,D.E. and Henrickson,K.J.
EPI_ISL_2584742	J. Craig Venter Institute	J. Craig Venter Institute	Shabman,R., Das,S.R., Shilts,M., Fedorova,N., Puri,V., Shrivastava,S., Amedeo,P., Williams,M., Barratt,K., Mitchell,J. and Jennings,L.
EPI_ISL_2584745	J. Craig Venter Institute	J. Craig Venter Institute	Das,S.R., Halpin,R.A., Puri,V., Akopov,A., Fedorova,N., Stockwell,T., Amedeo,P., Bishop,B., Katzel,D., Schobel,S., Shrivastava,S., Wentworth,D.E. and Caserta,M.
EPI_ISL_2584748, EPI_ISL_2584749	J. Craig Venter Institute	J. Craig Venter Institute	Das,S.R., Halpin,R.A., Puri,V., Akopov,A., Fedorova,N., Stockwell,T., Amedeo,P., Bishop,B., Katzel,D., Schobel,S., Shrivastava,S., Hall,C.B., Tesini,B.L., Schnabel,K.C., Walsh,E.E. and Caserta,M.
EPI_ISL_2584750, EPI_ISL_2584751	J. Craig Venter Institute	J. Craig Venter Institute	Das,S.R., Halpin,R.A., Puri,V., Akopov,A., Fedorova,N., Stockwell,T., Amedeo,P., Bishop,B., Katzel,D., Schobel,S., Shrivastava,S., Wentworth,D.E. and Caserta,M.
EPI_ISL_2584752	J. Craig Venter Institute	J. Craig Venter Institute	Das,S.R., Halpin,R.A., Puri,V., Akopov,A., Fedorova,N., Stockwell,T., Amedeo,P., Bishop,B., Katzel,D., Schobel,S., Shrivastava,S., Hall,C.B., Tesini,B.L., Schnabel,K.C., Walsh,E.E. and Caserta,M.
EPI_ISL_2584753	J. Craig Venter Institute	J. Craig Venter Institute	Das,S.R., Halpin,R.A., Puri,V., Akopov,A., Fedorova,N., Tsitrin,T., Stockwell,T., Amedeo,P., Bishop,B., Gupta,N., Hoover,J., Katzel,D., Schobel,S., Shrivastava,S., Wentworth,D.E. and Caserta,M.
EPI_ISL_2584754, EPI_ISL_2584755, EPI_ISL_2584756	J. Craig Venter Institute	J. Craig Venter Institute	Das,S.R., Halpin,R.A., Puri,V., Akopov,A., Fedorova,N., Stockwell,T., Amedeo,P., Bishop,B., Katzel,D., Schobel,S., Shrivastava,S., Wentworth,D.E. and Caserta,M.
EPI_ISL_2584760	J. Craig Venter Institute	J. Craig Venter Institute	Das,S.R., Halpin,R.A., Puri,V., Akopov,A., Fedorova,N., Tsitrin,T., Stockwell,T., Amedeo,P., Bishop,B., Gupta,N., Hoover,J., Katzel,D., Schobel,S., Shrivastava,S., Wentworth,D.E. and Caserta,M.
EPI_ISL_2584762	J. Craig Venter Institute	J. Craig Venter Institute	Das,S.R., Halpin,R.A., Puri,V., Akopov,A., Fedorova,N., Stockwell,T., Amedeo,P., Bishop,B., Katzel,D., Schobel,S., Shrivastava,S., Hall,C.B., Tesini,B.L., Schnabel,K.C., Walsh,E.E. and Caserta,M.
EPI_ISL_2584764	J. Craig Venter Institute	J. Craig Venter Institute	Das,S.R., Halpin,R.A., Puri,V., Akopov,A., Fedorova,N., Stockwell,T., Amedeo,P., Bishop,B., Katzel,D., Schobel,S., Shrivastava,S., Wentworth,D.E. and Caserta,M.
EPI_ISL_2584765	J. Craig Venter Institute	J. Craig Venter Institute	Das,S.R., Halpin,R.A., Puri,V., Akopov,A., Fedorova,N., Stockwell,T., Amedeo,P., Bishop,B., Katzel,D., Schobel,S., Shrivastava,S., Hall,C.B., Tesini,B.L., Schnabel,K.C., Walsh,E.E. and Caserta,M.
EPI_ISL_2584766	J. Craig Venter Institute	J. Craig Venter Institute	Das,S.R., Halpin,R.A., Puri,V., Akopov,A., Fedorova,N., Stockwell,T., Amedeo,P., Bishop,B., Katzel,D., Schobel,S., Shrivastava,S., Wentworth,D.E. and Caserta,M.
EPI_ISL_2584768, EPI_ISL_2584770, EPI_ISL_2584771	J. Craig Venter Institute	J. Craig Venter Institute	Das,S.R., Halpin,R.A., Puri,V., Akopov,A., Fedorova,N., Stockwell,T., Amedeo,P., Bishop,B., Katzel,D., Schobel,S., Shrivastava,S., Hall,C.B., Tesini,B.L., Schnabel,K.C., Walsh,E.E. and Caserta,M.
EPI_ISL_2584773, EPI_ISL_2584774	Epidemiology and Demography, KEMRI-Wellcome Trust	Epidemiology and Demography, KEMRI-Wellcome Trust	Kamau,E., Otieno,J.R., Murunga,N., Nyiro,J.U., Oketch,J.W., Ngoi,J.M., de Laurent,Z.R., Mwema,A., Agoti,C.N. and Nokes,D.J.
EPI_ISL_2584812	J. Craig Venter Institute	J. Craig Venter Institute	Shabman,R., Das,S.R., Puri,V., Fedorova,N., Amedeo,P., Williams,M., Shrivastava,S. and Halasa,N.
EPI_ISL_2584814	J. Craig Venter Institute	J. Craig Venter Institute	Das,S.R., Halpin,R.A., Puri,V., Akopov,A., Fedorova,N., Tsitrin,T., Stockwell,T., Amedeo,P., Bishop,B., Gupta,N., Hoover,J., Katzel,D., Schobel,S., Shrivastava,S., Wentworth,D.E. and Caserta,M.
EPI_ISL_2584815	J. Craig Venter Institute	J. Craig Venter Institute	Das,S., Halpin,R.A., Bera,J., Fedorova,N., Tsitrin,T., Stockwell,T., Amedeo,P., Bishop,B., Gupta,N., Hoover,J., Katzel,D., Schobel,S., Shrivastava,S., Hartert,T., Moore,M., Chappell,J., Larkin,E., Wentworth,D.E. and Anderson,L.J.
EPI_ISL_2584817	J. Craig Venter Institute	J. Craig Venter Institute	Das,S.R., Halpin,R.A., Puri,V., Akopov,A., Fedorova,N., Stockwell,T., Amedeo,P., Bishop,B., Katzel,D., Schobel,S., Shrivastava,S., Wentworth,D.E. and Caserta,M.
EPI_ISL_2584818	J. Craig Venter Institute	J. Craig Venter Institute	Das,S.R., Halpin,R.A., Puri,V., Akopov,A., Fedorova,N., Tsitrin,T., Stockwell,T., Amedeo,P., Bishop,B., Gupta,N., Hoover,J., Katzel,D., Schobel,S., Shrivastava,S., Wentworth,D.E. and Caserta,M.
EPI_ISL_2584819, EPI_ISL_2584820	J. Craig Venter Institute	J. Craig Venter Institute	Das,S.R., Halpin,R.A., Puri,V., Akopov,A., Fedorova,N., Stockwell,T., Amedeo,P., Bishop,B., Katzel,D., Schobel,S., Shrivastava,S., Hall,C.B., Tesini,B.L.,

			Schnabel,K.C., Walsh,E.E. and Caserta,M.
EPI_ISL_2584824, EPI_ISL_2584847	KEMRI Wellcome Trust Research Programme	KEMRI Wellcome Trust Research Programme	Agoti,C.N., Otieno,J.R., Munywoki,P.K., Mwihuri,A.G., Cane,P.A., Nokes,D.J., Kellam,P. and Cotten,M.L.
EPI_ISL_2584849	J. Craig Venter Institute	J. Craig Venter Institute	Wentworth,D.E., Halpin,R.A., Bera,J., Lin,X., Fedorova,N., Tsitrin,T., McLellan,M., Stockwell,T., Amedeo,P., Bishop,B., Gupta,N., Hoover,J., Katzel,D., Schobel,S., Shrivastava,S., Garcia,J., Laguna-Torres,V.A., Leguia,M., Benavides,J.G. and Halsey,E.
EPI_ISL_2584850	J. Craig Venter Institute	J. Craig Venter Institute	Das,S.R., Halpin,R.A., Puri,V., Akopov,A., Fedorova,N., Tsitrin,T., Stockwell,T., Amedeo,P., Bishop,B., Gupta,N., Hoover,J., Katzel,D., Schobel,S., Shrivastava,S., Wentworth,D.E. and Caserta,M.
EPI_ISL_2584852	Medical Microbiology, University Medical Center Utrecht	Medical Microbiology, University Medical Center Utrecht	Tan,L., Viveen,M.C., Lemey,P. and Coenjaerts,F.E.
EPI_ISL_2584853	J. Craig Venter Institute	J. Craig Venter Institute	Shabman,R., Das,S.R., Shilts,M., Fedorova,N., Puri,V., Shrivastava,S., Amedeo,P., Williams,M., Barratt,K., Mitchell,J. and Jennings,L.
EPI_ISL_2584855	J. Craig Venter Institute	J. Craig Venter Institute	Das,S., Halpin,R.A., Bera,J., Puri,V., Fedorova,N., Tsitrin,T., Stockwell,T., Amedeo,P., Bishop,B., Katzel,D., Schobel,S., Shrivastava,S., Hartert,T., Moore,M., Chappell,J., Larkin,E., Wentworth,D.E. and Anderson,L.J.
EPI_ISL_2584856	Epidemiology and Demography, KEMRI-Wellcome Trust	Epidemiology and Demography, KEMRI-Wellcome Trust	Kamau,E., Otieno,J.R., Murunga,N., Nyiro,J.U., Oketch,J.W., Ngoi,J.M., de Laurent,Z.R., Mwema,A., Agoti,C.N. and Nokes,D.J.
EPI_ISL_2584859	KEMRI Wellcome Trust Research Programme	KEMRI Wellcome Trust Research Programme	Agoti,C.N., Otieno,J.R., Munywoki,P.K., Mwihuri,A.G., Cane,P.A., Nokes,D.J., Kellam,P. and Cotten,M.L.
EPI_ISL_2584860	J. Craig Venter Institute	J. Craig Venter Institute	Shabman,R., Das,S.R., Shilts,M., Fedorova,N., Puri,V., Shrivastava,S., Amedeo,P., Williams,M., Barratt,K., Mitchell,J. and Jennings,L.
EPI_ISL_2584861	J. Craig Venter Institute	J. Craig Venter Institute	Wentworth,D.E., Halpin,R.A., Bera,J., Lin,X., Fedorova,N., Tsitrin,T., McLellan,M., Stockwell,T., Amedeo,P., Bishop,B., Gupta,N., Hoover,J., Katzel,D., Schobel,S., Shrivastava,S., Garcia,J., Laguna-Torres,V.A., Leguia,M., Benavides,J.G. and Halsey,E.
EPI_ISL_2584862	Michiko Okamoto Tohoku University Graduate School of Medicine, Virology	Michiko Okamoto Tohoku University Graduate School of Medicine, Virology	Okamoto,M., Malasaor,R. and Oshitani,H.
EPI_ISL_2584863	J. Craig Venter Institute	J. Craig Venter Institute	Das,S.R., Halpin,R.A., Shilts,M., Puri,V., Akopov,A., Fedorova,N., Stockwell,T., Amedeo,P., Bishop,B., Katzel,D., Schobel,S., Shrivastava,S. and Hartert,T.
EPI_ISL_2584865	J. Craig Venter Institute	J. Craig Venter Institute	Shabman,R., Das,S.R., Puri,V., Fedorova,N., Amedeo,P., Williams,M., Shrivastava,S. and Halasa,N.
EPI_ISL_2584866, EPI_ISL_2584867, EPI_ISL_2584868, EPI_ISL_2584869, EPI_ISL_2584871, EPI_ISL_2584872	Epidemiology and Demography, KEMRI-Wellcome Trust	Epidemiology and Demography, KEMRI-Wellcome Trust	Kamau,E., Otieno,J.R., Murunga,N., Nyiro,J.U., Oketch,J.W., Ngoi,J.M., de Laurent,Z.R., Mwema,A., Agoti,C.N. and Nokes,D.J.
EPI_ISL_2584873, EPI_ISL_2584874, EPI_ISL_2584875	J. Craig Venter Institute	J. Craig Venter Institute	Wentworth,D.E., Halpin,R.A., Bera,J., Lin,X., Fedorova,N., Tsitrin,T., McLellan,M., Stockwell,T., Amedeo,P., Bishop,B., Gupta,N., Hoover,J., Katzel,D., Schobel,S., Shrivastava,S., Garcia,J., Laguna-Torres,V.A., Leguia,M., Benavides,J.G. and Halsey,E.
EPI_ISL_2584879	J. Craig Venter Institute	J. Craig Venter Institute	Das,S., Halpin,R.A., Bera,J., Puri,V., Fedorova,N., Tsitrin,T., Stockwell,T., Amedeo,P., Bishop,B., Katzel,D., Schobel,S., Shrivastava,S., Hartert,T., Moore,M., Chappell,J., Larkin,E., Wentworth,D.E. and Anderson,L.J.
EPI_ISL_2584880	J. Craig Venter Institute	J. Craig Venter Institute	Das,S.R., Halpin,R.A., Shilts,M., Puri,V., Akopov,A., Fedorova,N., Stockwell,T., Amedeo,P., Bishop,B., Katzel,D., Schobel,S., Shrivastava,S. and Hartert,T.
EPI_ISL_2584881, EPI_ISL_2584882, EPI_ISL_2584883, EPI_ISL_2584884, EPI_ISL_2584885, EPI_ISL_2584886, EPI_ISL_2584887, EPI_ISL_2584888, EPI_ISL_2584889	Epidemiology and Demography, KEMRI-Wellcome Trust	Epidemiology and Demography, KEMRI-Wellcome Trust	Kamau,E., Otieno,J.R., Murunga,N., Nyiro,J.U., Oketch,J.W., Ngoi,J.M., de Laurent,Z.R., Mwema,A., Agoti,C.N. and Nokes,D.J.
EPI_ISL_2584890	Medical Microbiology, University Medical Center Utrecht	Medical Microbiology, University Medical Center Utrecht	Tan,L., Lemey,P., Viveen,M. and Coenjaerts,F.E.J.
EPI_ISL_2584891, EPI_ISL_2584893	J. Craig Venter Institute	J. Craig Venter Institute	Shabman,R., Das,S.R., Shilts,M., Fedorova,N., Puri,V., Shrivastava,S., Amedeo,P., Williams,M., Barratt,K., Mitchell,J. and Jennings,L.
EPI_ISL_2584894	J. Craig Venter Institute	J. Craig Venter Institute	Wentworth,D.E., Halpin,R.A., Bera,J., Lin,X., Fedorova,N., Tsitrin,T., McLellan,M., Stockwell,T., Amedeo,P., Bishop,B., Gupta,N., Hoover,J., Katzel,D., Schobel,S., Shrivastava,S., Garcia,J., Laguna-Torres,V.A., Leguia,M., Benavides,J.G. and Halsey,E.
EPI_ISL_2584896	Medicine, University of Washington, 300 9th Ave, Harborview Research & Training Building	Medicine, University of Washington, 300 9th Ave, Harborview Research & Training Building	Chu,H., Scott,E. and Roychoudhury,P.
EPI_ISL_2584897	J. Craig Venter Institute	J. Craig Venter Institute	Das,S., Halpin,R.A., Bera,J., Fedorova,N., Tsitrin,T., Stockwell,T., Amedeo,P., Bishop,B., Gupta,N., Hoover,J., Katzel,D., Schobel,S., Shrivastava,S., Hartert,T., Moore,M., Chappell,J., Larkin,E., Wentworth,D.E. and Anderson,L.J.
EPI_ISL_2584898, EPI_ISL_2584899, EPI_ISL_2584901, EPI_ISL_2584902, EPI_ISL_2584904, EPI_ISL_2584905, EPI_ISL_2584906, EPI_ISL_2584907, EPI_ISL_2584909	J. Craig Venter Institute	J. Craig Venter Institute	Das,S.R., Halpin,R.A., Shilts,M., Puri,V., Akopov,A., Fedorova,N., Stockwell,T., Amedeo,P., Bishop,B., Katzel,D., Schobel,S., Shrivastava,S. and Hartert,T.
EPI_ISL_2584912, EPI_ISL_2584913, EPI_ISL_2584914, EPI_ISL_2584915, EPI_ISL_2584916, EPI_ISL_2584917, EPI_ISL_2584918, EPI_ISL_2584919, EPI_ISL_2584920, EPI_ISL_2584921, EPI_ISL_2584922, EPI_ISL_2584923, EPI_ISL_2584924, EPI_ISL_2584925, EPI_ISL_2584926, EPI_ISL_2584927, EPI_ISL_2584928, EPI_ISL_2584929, EPI_ISL_2584930, EPI_ISL_2584931, EPI_ISL_2584932, EPI_ISL_2584933, EPI_ISL_2584934, EPI_ISL_2584935			
see above	Epidemiology and Demography, KEMRI-Wellcome Trust	Epidemiology and Demography, KEMRI-Wellcome Trust	Kamau,E., Otieno,J.R., Murunga,N., Nyiro,J.U., Oketch,J.W., Ngoi,J.M., de Laurent,Z.R., Mwema,A., Agoti,C.N. and Nokes,D.J.
EPI_ISL_2584937	Medical Microbiology, University Medical Center Utrecht	Medical Microbiology, University Medical Center Utrecht	Tan,L., Viveen,M.C., Lemey,P. and Coenjaerts,F.E.
EPI_ISL_2584938, EPI_ISL_2584939, EPI_ISL_2584940	J. Craig Venter Institute	J. Craig Venter Institute	Shabman,R., Das,S.R., Shilts,M., Fedorova,N., Puri,V., Shrivastava,S., Amedeo,P., Williams,M., Barratt,K., Mitchell,J. and Jennings,L.
EPI_ISL_2584941	Medicine, University of Washington, 300 9th Ave, Harborview Research & Training Building	Medicine, University of Washington, 300 9th Ave, Harborview Research & Training Building	Chu,H., Scott,E. and Roychoudhury,P.
EPI_ISL_2584944, EPI_ISL_2584945, EPI_ISL_2584946, EPI_ISL_2584947, EPI_ISL_2584948, EPI_ISL_2584950, EPI_ISL_2584952, EPI_ISL_2584953	J. Craig Venter Institute	J. Craig Venter Institute	Das,S.R., Halpin,R.A., Shilts,M., Puri,V., Akopov,A., Fedorova,N., Stockwell,T., Amedeo,P., Bishop,B., Katzel,D., Schobel,S., Shrivastava,S. and Hartert,T.
EPI_ISL_2584955, EPI_ISL_2584956, EPI_ISL_2584958, EPI_ISL_2584959, EPI_ISL_2584960, EPI_ISL_2584961, EPI_ISL_2584963, EPI_ISL_2584964, EPI_ISL_2584965, EPI_ISL_2584967, EPI_ISL_2584968, EPI_ISL_2584969, EPI_ISL_2584970, EPI_ISL_2584972, EPI_ISL_2584973, EPI_ISL_2584975, EPI_ISL_2584976, EPI_ISL_2584977, EPI_ISL_2584978, EPI_ISL_2584980, EPI_ISL_2584981, EPI_ISL_2584982, EPI_ISL_2584983, EPI_ISL_2584985			
see above	Epidemiology and Demography, KEMRI-Wellcome Trust	Epidemiology and Demography, KEMRI-Wellcome Trust	Kamau,E., Otieno,J.R., Murunga,N., Nyiro,J.U., Oketch,J.W., Ngoi,J.M., de Laurent,Z.R., Mwema,A., Agoti,C.N. and Nokes,D.J.
EPI_ISL_2584986, EPI_ISL_2584987	Medical Microbiology, University Medical Center Utrecht	Medical Microbiology, University Medical Center Utrecht	Tan,L., Viveen,M.C., Lemey,P. and Coenjaerts,F.E.
EPI_ISL_2584988, EPI_ISL_2584989, EPI_ISL_2584990, EPI_ISL_2584991	J. Craig Venter Institute	J. Craig Venter Institute	Shabman,R., Das,S.R., Shilts,M., Fedorova,N., Puri,V., Shrivastava,S., Amedeo,P., Williams,M., Barratt,K., Mitchell,J. and Jennings,L.
EPI_ISL_2584992, EPI_ISL_2584993	J. Craig Venter Institute	J. Craig Venter Institute	Wentworth,D.E., Halpin,R.A., Bera,J., Lin,X., Fedorova,N., Tsitrin,T., McLellan,M., Stockwell,T., Amedeo,P., Bishop,B., Gupta,N., Hoover,J., Katzel,D., Schobel,S., Shrivastava,S., Garcia,J., Laguna-Torres,V.A., Leguia,M., Benavides,J.G. and Halsey,E.
EPI_ISL_2584994	Division of Biosafety Evaluation and Control, Korea National Institute of Health	Division of Biosafety Evaluation and Control, Korea National Institute of Health	Yun,M.-R., Lee,W.-J., Kim,A.-R., Lee,H.S., Kim,K., Kim,S.S., Kim,Y.-J. and Kim,D.-W.
EPI_ISL_2584995, EPI_ISL_2584996, EPI_ISL_2584997, EPI_ISL_2584998, EPI_ISL_2584999, EPI_ISL_2585000, EPI_ISL_2585001, EPI_ISL_2585002, EPI_ISL_2585003, EPI_ISL_2585004, EPI_ISL_2585005, EPI_ISL_2585006, EPI_ISL_2585007, EPI_ISL_2585009, EPI_ISL_2585010, EPI_ISL_2585012, EPI_ISL_2585013, EPI_ISL_2585014, EPI_ISL_2585015			

see above	J. Craig Venter Institute	J. Craig Venter Institute	Das,S.R., Halpin,R.A., Shilts,M., Puri,V., Akopov,A., Fedorova,N., Stockwell,T., Amedeo,P., Bishop,B., Katzel,D., Schobel,S., Shrivastava,S. and Hartert,T.
EPI_ISL_2585016	J. Craig Venter Institute	J. Craig Venter Institute	Shabman,R., Das,S.R., Puri,V., Fedorova,N., Amedeo,P., Williams,M., Shrivastava,S. and Halasa,N.
EPI_ISL_2585017, EPI_ISL_2585018, EPI_ISL_2585019, EPI_ISL_2585020, EPI_ISL_2585021, EPI_ISL_2585022, EPI_ISL_2585023, EPI_ISL_2585025, EPI_ISL_2585026, EPI_ISL_2585027, EPI_ISL_2585028, EPI_ISL_2585029, EPI_ISL_2585031, EPI_ISL_2585032, EPI_ISL_2585033, EPI_ISL_2585036, EPI_ISL_2585037, EPI_ISL_2585039, EPI_ISL_2585040, EPI_ISL_2585041, EPI_ISL_2585042, EPI_ISL_2585043, EPI_ISL_2585044, EPI_ISL_2585045, EPI_ISL_2585047, EPI_ISL_2585049, EPI_ISL_2585050, EPI_ISL_2585051, EPI_ISL_2585052, EPI_ISL_2585053, EPI_ISL_2585054, EPI_ISL_2585056, EPI_ISL_2585057, EPI_ISL_2585058, EPI_ISL_2585059, EPI_ISL_2585060, EPI_ISL_2585061			
see above	Epidemiology and Demography, KEMRI-Wellcome Trust	Epidemiology and Demography, KEMRI-Wellcome Trust	Kamau,E., Otieno,J.R., Murunga,N., Nyiro,J.U., Oketch,J.W., Ngoi,J.M., de Laurent,Z.R., Mwema,A., Agoti,C.N. and Nokes,D.J.
EPI_ISL_2585063	Medical Microbiology, University Medical Center Utrecht	Medical Microbiology, University Medical Center Utrecht	Tan,L., Viveen,M.C., Lemey,P. and Coenjaerts,F.E.
EPI_ISL_2585064, EPI_ISL_2585065, EPI_ISL_2585066, EPI_ISL_2585067, EPI_ISL_2585068, EPI_ISL_2585070, EPI_ISL_2585072	J. Craig Venter Institute	J. Craig Venter Institute	Shabman,R., Das,S.R., Shilts,M., Fedorova,N., Puri,V., Shrivastava,S., Amedeo,P., Williams,M., Barratt,K., Mitchell,J. and Jennings,L.
EPI_ISL_2585074	J. Craig Venter Institute	J. Craig Venter Institute	Lorenzi,H., Town,C., Halpin,R., Bera,J., Ransier,A., Fedorova,N., Stockwell,T., Amedeo,P., Appalla,L., Bishop,B., Edworthy,P., Gupta,N., Hoover,J., Katzel,D., Li,K., Schobel,S., Shrivastava,S., Thovarai,V., Wang,S., Rebuffo-Scheer,C., Fan,J., He,J., Kehl,S.C., Lederboer,N., Jurgens,L.A., Bose,M.E., Beck,E.T., Kumar,S., Wentworth,D.E. and Henrickson,K.J.
EPI_ISL_2585075	J. Craig Venter Institute	J. Craig Venter Institute	Das,S.R., Halpin,R.A., Shilts,M., Puri,V., Akopov,A., Fedorova,N., Stockwell,T., Amedeo,P., Bishop,B., Katzel,D., Schobel,S., Shrivastava,S. and Hartert,T.
EPI_ISL_2585077	J. Craig Venter Institute	J. Craig Venter Institute	Shabman,R., Das,S.R., Shilts,M., Fedorova,N., Puri,V., Shrivastava,S., Amedeo,P., Hu,L., Durbin,A., Rocchi,I., Williams,T. and Hartert,T.
EPI_ISL_2585078, EPI_ISL_2585079, EPI_ISL_2585080, EPI_ISL_2585081, EPI_ISL_2585082, EPI_ISL_2585083, EPI_ISL_2585084, EPI_ISL_2585085, EPI_ISL_2585086, EPI_ISL_2585087, EPI_ISL_2585091, EPI_ISL_2585093, EPI_ISL_2585094			
see above	J. Craig Venter Institute	J. Craig Venter Institute	Das,S.R., Halpin,R.A., Shilts,M., Puri,V., Akopov,A., Fedorova,N., Stockwell,T., Amedeo,P., Bishop,B., Katzel,D., Schobel,S., Shrivastava,S. and Hartert,T.
EPI_ISL_2585095	J. Craig Venter Institute	J. Craig Venter Institute	Shabman,R., Das,S.R., Puri,V., Fedorova,N., Amedeo,P., Williams,M., Shrivastava,S. and Halasa,N.
EPI_ISL_2585096, EPI_ISL_2585098, EPI_ISL_2585099, EPI_ISL_2585100, EPI_ISL_2585101, EPI_ISL_2585102, EPI_ISL_2585103, EPI_ISL_2585104, EPI_ISL_2585105, EPI_ISL_2585106, EPI_ISL_2585107, EPI_ISL_2585108, EPI_ISL_2585110, EPI_ISL_2585111, EPI_ISL_2585112, EPI_ISL_2585113, EPI_ISL_2585114, EPI_ISL_2585115, EPI_ISL_2585116, EPI_ISL_2585117, EPI_ISL_2585118, EPI_ISL_2585119, EPI_ISL_2585120, EPI_ISL_2585121, EPI_ISL_2585123, EPI_ISL_2585124, EPI_ISL_2585125, EPI_ISL_2585126, EPI_ISL_2585127, EPI_ISL_2585128, EPI_ISL_2585130, EPI_ISL_2585131, EPI_ISL_2585132, EPI_ISL_2585133, EPI_ISL_2585134, EPI_ISL_2585135, EPI_ISL_2585136, EPI_ISL_2585137, EPI_ISL_2585139, EPI_ISL_2585140			
see above	Epidemiology and Demography, KEMRI-Wellcome Trust	Epidemiology and Demography, KEMRI-Wellcome Trust	Kamau,E., Otieno,J.R., Murunga,N., Nyiro,J.U., Oketch,J.W., Ngoi,J.M., de Laurent,Z.R., Mwema,A., Agoti,C.N. and Nokes,D.J.
EPI_ISL_2585142	Medical Microbiology, University Medical Center Utrecht	Medical Microbiology, University Medical Center Utrecht	Tan,L., Viveen,M.C., Lemey,P. and Coenjaerts,F.E.
EPI_ISL_2585143	J. Craig Venter Institute	J. Craig Venter Institute	Shabman,R., Das,S.R., Shilts,M., Fedorova,N., Puri,V., Shrivastava,S., Amedeo,P., Williams,M., Barratt,K., Mitchell,J. and Jennings,L.
EPI_ISL_2585144	J. Craig Venter Institute	J. Craig Venter Institute	Wentworth,D.E., Halpin,R.A., Bera,J., Lin,X., Fedorova,N., Tsitrin,T., McLellan,M., Stockwell,T., Amedeo,P., Bishop,B., Gupta,N., Hoover,J., Katzel,D., Schobel,S., Shrivastava,S., Garcia,J., Laguna-Torres,V.A., Leguia,M., Benavides,J.G. and Halsey,E.
EPI_ISL_2585146	Taylor G.S., Virology laboratory, Bristol Public Health Laboratory	Taylor G.S., Virology laboratory, Bristol Public Health Laboratory	Taylor,G.S.
EPI_ISL_2585147, EPI_ISL_2585148	Medicine, University of Washington, 300 9th Ave, Harborview Research & Training Building	Medicine, University of Washington, 300 9th Ave, Harborview Research & Training Building	Chu,H., Scott,E. and Roychoudhury,P.
EPI_ISL_2585149, EPI_ISL_2585150	J. Craig Venter Institute	J. Craig Venter Institute	Das,S.R., Halpin,R.A., Shilts,M., Puri,V., Akopov,A., Fedorova,N., Stockwell,T., Amedeo,P., Bishop,B., Katzel,D., Schobel,S., Shrivastava,S. and Hartert,T.
EPI_ISL_2585153, EPI_ISL_2585154, EPI_ISL_2585155	J. Craig Venter Institute	J. Craig Venter Institute	Shabman,R., Das,S.R., Puri,V., Fedorova,N., Amedeo,P., Williams,M., Shrivastava,S. and Halasa,N.
EPI_ISL_2585156, EPI_ISL_2585157, EPI_ISL_2585158, EPI_ISL_2585160, EPI_ISL_2585161, EPI_ISL_2585162, EPI_ISL_2585163, EPI_ISL_2585164, EPI_ISL_2585165, EPI_ISL_2585167, EPI_ISL_2585168, EPI_ISL_2585169, EPI_ISL_2585171, EPI_ISL_2585172, EPI_ISL_2585173, EPI_ISL_2585174, EPI_ISL_2585175, EPI_ISL_2585176, EPI_ISL_2585177, EPI_ISL_2585178, EPI_ISL_2585179, EPI_ISL_2585180, EPI_ISL_2585181, EPI_ISL_2585182, EPI_ISL_2585183, EPI_ISL_2585184, EPI_ISL_2585185, EPI_ISL_2585186, EPI_ISL_2585187, EPI_ISL_2585188			
see above	Epidemiology and Demography, KEMRI-Wellcome Trust	Epidemiology and Demography, KEMRI-Wellcome Trust	Kamau,E., Otieno,J.R., Murunga,N., Nyiro,J.U., Oketch,J.W., Ngoi,J.M., de Laurent,Z.R., Mwema,A., Agoti,C.N. and Nokes,D.J.
EPI_ISL_2585189, EPI_ISL_2585190	Medical Microbiology, University Medical Center Utrecht	Medical Microbiology, University Medical Center Utrecht	Tan,L., Viveen,M.C., Lemey,P. and Coenjaerts,F.E.
EPI_ISL_2585191, EPI_ISL_2585192, EPI_ISL_2585193, EPI_ISL_2585195	J. Craig Venter Institute	J. Craig Venter Institute	Shabman,R., Das,S.R., Shilts,M., Fedorova,N., Puri,V., Shrivastava,S., Amedeo,P., Williams,M., Barratt,K., Mitchell,J. and Jennings,L.
EPI_ISL_2585196	Medicine, University of Washington, 300 9th Ave, Harborview Research & Training Building	Medicine, University of Washington, 300 9th Ave, Harborview Research & Training Building	Chu,H., Scott,E. and Roychoudhury,P.
EPI_ISL_2585199, EPI_ISL_2585200	J. Craig Venter Institute	J. Craig Venter Institute	Das,S.R., Halpin,R.A., Shilts,M., Puri,V., Akopov,A., Fedorova,N., Stockwell,T., Amedeo,P., Bishop,B., Katzel,D., Schobel,S., Shrivastava,S. and Hartert,T.
EPI_ISL_2585205	J. Craig Venter Institute	J. Craig Venter Institute	Lorenzi,H., Town,C., Halpin,R., Bera,J., Ransier,A., Fedorova,N., Stockwell,T., Amedeo,P., Appalla,L., Bishop,B., Edworthy,P., Gupta,N., Hoover,J., Katzel,D., Li,K., Schobel,S., Shrivastava,S., Thovarai,V., Wang,S., Rebuffo-Scheer,C., Fan,J., He,J., Kehl,S.C., Lederboer,N., Jurgens,L.A., Bose,M.E., Beck,E.T., Kumar,S., Noyola,D.E., Wentworth,D.E. and Henrickson,K.J.
EPI_ISL_2585247	J. Craig Venter Institute	J. Craig Venter Institute	Das,S.R., Halpin,R.A., Puri,V., Akopov,A., Fedorova,N., Stockwell,T., Amedeo,P., Bishop,B., Katzel,D., Schobel,S., Shrivastava,S., Hall,C.B., Tesini,B.L., Schnabel,K.C., Walsh,E.E. and Caserta,M.
EPI_ISL_2585256, EPI_ISL_2585258	J. Craig Venter Institute	J. Craig Venter Institute	Shabman,R., Das,S.R., Shilts,M., Fedorova,N., Puri,V., Shrivastava,S., Amedeo,P., Williams,M., Barratt,K., Mitchell,J. and Jennings,L.
EPI_ISL_2585259	J. Craig Venter Institute	J. Craig Venter Institute	Das,S., Halpin,R.A., Bera,J., Puri,V., Fedorova,N., Tsitrin,T., Stockwell,T., Amedeo,P., Bishop,B., Katzel,D., Schobel,S., Shrivastava,S., Hartert,T., Moore,M., Chappell,J., Larkin,E., Wentworth,D.E. and Anderson,L.J.
EPI_ISL_2585260	J. Craig Venter Institute	J. Craig Venter Institute	Das,S.R., Halpin,R.A., Shilts,M., Puri,V., Akopov,A., Fedorova,N., Stockwell,T., Amedeo,P., Bishop,B., Katzel,D., Schobel,S., Shrivastava,S. and Hartert,T.
EPI_ISL_2585261	J. Craig Venter Institute	J. Craig Venter Institute	Shabman,R., Das,S.R., Shilts,M., Fedorova,N., Puri,V., Shrivastava,S., Amedeo,P., Williams,M., Barratt,K., Mitchell,J. and Jennings,L.
EPI_ISL_2585265	J. Craig Venter Institute	J. Craig Venter Institute	Das,S.R., Halpin,R.A., Shilts,M., Puri,V., Akopov,A., Fedorova,N., Stockwell,T., Amedeo,P., Bishop,B., Katzel,D., Schobel,S., Shrivastava,S. and Hartert,T.
EPI_ISL_2585267, EPI_ISL_2585268, EPI_ISL_2585269, EPI_ISL_2585275, EPI_ISL_2585276, EPI_ISL_2585277, EPI_ISL_2585279	J. Craig Venter Institute	J. Craig Venter Institute	Shabman,R., Das,S.R., Shilts,M., Fedorova,N., Puri,V., Shrivastava,S., Amedeo,P., Williams,M., Barratt,K., Mitchell,J. and Jennings,L.
EPI_ISL_2585280	J. Craig Venter Institute	J. Craig Venter Institute	Das,S., Halpin,R.A., Bera,J., Fedorova,N., Tsitrin,T., Stockwell,T., Amedeo,P., Bishop,B., Gupta,N., Hoover,J., Katzel,D., Schobel,S., Shrivastava,S., Hartert,T., Moore,M., Chappell,J., Larkin,E., Wentworth,D.E. and Anderson,L.J.
EPI_ISL_2585281	J. Craig Venter Institute	J. Craig Venter Institute	Das,S.R., Halpin,R.A., Shilts,M., Puri,V., Akopov,A., Fedorova,N., Stockwell,T., Amedeo,P., Bishop,B., Katzel,D., Schobel,S., Shrivastava,S. and Hartert,T.
EPI_ISL_2585284	J. Craig Venter Institute	J. Craig Venter Institute	Shabman,R., Das,S.R., Puri,V., Fedorova,N., Amedeo,P., Williams,M., Shrivastava,S. and Halasa,N.
EPI_ISL_2585285	J. Craig Venter Institute	J. Craig Venter Institute	Das,S.R., Halpin,R.A., Shilts,M., Puri,V., Akopov,A., Fedorova,N., Stockwell,T., Amedeo,P., Bishop,B., Katzel,D., Schobel,S., Shrivastava,S. and Hartert,T.
EPI_ISL_2585286	J. Craig Venter Institute	J. Craig Venter Institute	Das,S., Halpin,R.A., Bera,J., Fedorova,N., Tsitrin,T., Stockwell,T., Amedeo,P., Bishop,B., Gupta,N., Hoover,J., Katzel,D., Schobel,S., Shrivastava,S., Hartert,T., Moore,M., Chappell,J., Larkin,E., Wentworth,D.E. and Anderson,L.J.
EPI_ISL_2585288, EPI_ISL_2585290	J. Craig Venter Institute	J. Craig Venter Institute	Das,S.R., Halpin,R.A., Shilts,M., Puri,V., Akopov,A., Fedorova,N., Stockwell,T., Amedeo,P., Bishop,B., Katzel,D., Schobel,S., Shrivastava,S. and Hartert,T.
EPI_ISL_2585291	J. Craig Venter Institute	J. Craig Venter Institute	Shabman,R., Das,S.R., Shilts,M., Fedorova,N., Puri,V., Shrivastava,S., Amedeo,P., Williams,M., Barratt,K., Mitchell,J. and Jennings,L.

EPI_ISL_2585292	Laboratory Medicine, UW Virology	Laboratory Medicine, UW Virology	Lin,M.J., Tait,A. and Greninger,A.L.
EPI_ISL_2585302	J. Craig Venter Institute	J. Craig Venter Institute	Das,S., Halpin,R.A., Bera,J., Puri,V., Fedorova,N., Tsitrin,T., Stockwell,T., Amedeo,P., Bishop,B., Katzel,D., Schobel,S., Shrivastava,S., Hartert,T., Moore,M., Chappell,J., Larkin,E., Wentworth,D.E. and Anderson,L.J.
EPI_ISL_2585307	J. Craig Venter Institute	J. Craig Venter Institute	Shabman,R., Das,S.R., Shilts,M., Fedorova,N., Puri,V., Shrivastava,S., Amedeo,P., Williams,M., Barratt,K., Mitchell,J. and Jennings,L.
EPI_ISL_2585309	J. Craig Venter Institute	J. Craig Venter Institute	Das,S.R., Halpin,R.A., Shilts,M., Puri,V., Akopov,A., Fedorova,N., Stockwell,T., Amedeo,P., Bishop,B., Katzel,D., Schobel,S., Shrivastava,S. and Hartert,T.
EPI_ISL_2585311, EPI_ISL_2585312, EPI_ISL_2585313, EPI_ISL_2585316, EPI_ISL_2585318	J. Craig Venter Institute	J. Craig Venter Institute	Shabman,R., Das,S.R., Shilts,M., Fedorova,N., Puri,V., Shrivastava,S., Amedeo,P., Williams,M., Barratt,K., Mitchell,J. and Jennings,L.
EPI_ISL_2585319	J. Craig Venter Institute	J. Craig Venter Institute	Das,S., Halpin,R.A., Bera,J., Fedorova,N., Tsitrin,T., Stockwell,T., Amedeo,P., Bishop,B., Gupta,N., Hoover,J., Katzel,D., Schobel,S., Shrivastava,S., Hartert,T., Moore,M., Chappell,J., Larkin,E., Wentworth,D.E. and Anderson,L.J.
EPI_ISL_2585320, EPI_ISL_2585323, EPI_ISL_2585325, EPI_ISL_2585327, EPI_ISL_2585329, EPI_ISL_2585332, EPI_ISL_2587159	J. Craig Venter Institute	J. Craig Venter Institute	Das,S.R., Halpin,R.A., Shilts,M., Puri,V., Akopov,A., Fedorova,N., Stockwell,T., Amedeo,P., Bishop,B., Katzel,D., Schobel,S., Shrivastava,S. and Hartert,T.
EPI_ISL_2587163	J. Craig Venter Institute	J. Craig Venter Institute	Shabman,R., Das,S.R., Puri,V., Fedorova,N., Amedeo,P., Williams,M., Shrivastava,S. and Halasa,N.
EPI_ISL_2587342	J. Craig Venter Institute	J. Craig Venter Institute	Shabman,R., Das,S.R., Shilts,M., Fedorova,N., Puri,V., Shrivastava,S., Amedeo,P., Williams,M., Barratt,K., Mitchell,J. and Jennings,L.
EPI_ISL_2587344	J. Craig Venter Institute	J. Craig Venter Institute	Shabman,R., Das,S.R., Shilts,M., Fedorova,N., Puri,V., Shrivastava,S., Amedeo,P., Hu,L., Durbin,A., Rocchi,I., Williams,T. and Hartert,T.
EPI_ISL_2587345	J. Craig Venter Institute	J. Craig Venter Institute	Das,S., Halpin,R.A., Bera,J., Fedorova,N., Tsitrin,T., Stockwell,T., Amedeo,P., Bishop,B., Gupta,N., Hoover,J., Katzel,D., Schobel,S., Shrivastava,S., Hartert,T., Moore,M., Chappell,J., Larkin,E., Wentworth,D.E. and Anderson,L.J.
EPI_ISL_2587349	J. Craig Venter Institute	J. Craig Venter Institute	Shabman,R., Das,S.R., Shilts,M., Fedorova,N., Puri,V., Shrivastava,S., Amedeo,P., Hu,L., Durbin,A., Rocchi,I., Williams,T. and Hartert,T.
EPI_ISL_2587351, EPI_ISL_2587353	J. Craig Venter Institute	J. Craig Venter Institute	Das,S., Halpin,R.A., Bera,J., Fedorova,N., Tsitrin,T., Stockwell,T., Amedeo,P., Bishop,B., Gupta,N., Hoover,J., Katzel,D., Schobel,S., Shrivastava,S., Hartert,T., Moore,M., Chappell,J., Larkin,E., Wentworth,D.E. and Anderson,L.J.
EPI_ISL_2587355, EPI_ISL_2587357	J. Craig Venter Institute	J. Craig Venter Institute	Das,S.R., Halpin,R.A., Shilts,M., Puri,V., Akopov,A., Fedorova,N., Stockwell,T., Amedeo,P., Bishop,B., Katzel,D., Schobel,S., Shrivastava,S. and Hartert,T.
EPI_ISL_2587358	J. Craig Venter Institute	J. Craig Venter Institute	Das,S., Halpin,R.A., Bera,J., Fedorova,N., Tsitrin,T., Stockwell,T., Amedeo,P., Bishop,B., Gupta,N., Hoover,J., Katzel,D., Schobel,S., Shrivastava,S., Hartert,T., Moore,M., Chappell,J., Larkin,E., Wentworth,D.E. and Anderson,L.J.
EPI_ISL_2587362, EPI_ISL_2587364	J. Craig Venter Institute	J. Craig Venter Institute	Das,S.R., Halpin,R.A., Shilts,M., Puri,V., Akopov,A., Fedorova,N., Stockwell,T., Amedeo,P., Bishop,B., Katzel,D., Schobel,S., Shrivastava,S. and Hartert,T.
EPI_ISL_2587366	J. Craig Venter Institute	J. Craig Venter Institute	Das,S., Halpin,R.A., Bera,J., Fedorova,N., Tsitrin,T., Stockwell,T., Amedeo,P., Bishop,B., Gupta,N., Hoover,J., Katzel,D., Schobel,S., Shrivastava,S., Hartert,T., Moore,M., Chappell,J., Larkin,E., Wentworth,D.E. and Anderson,L.J.
EPI_ISL_2587379, EPI_ISL_2587381, EPI_ISL_2587383, EPI_ISL_2587385, EPI_ISL_2587387	J. Craig Venter Institute	J. Craig Venter Institute	Shabman,R., Das,S.R., Shilts,M., Fedorova,N., Puri,V., Shrivastava,S., Amedeo,P., Williams,M., Barratt,K., Mitchell,J. and Jennings,L.
EPI_ISL_2587389	J. Craig Venter Institute	J. Craig Venter Institute	Shabman,R., Das,S.R., Puri,V., Fedorova,N., Amedeo,P., Williams,M., Shrivastava,S. and Halasa,N.
EPI_ISL_2587391, EPI_ISL_2587393, EPI_ISL_2587396, EPI_ISL_2587398, EPI_ISL_2587400, EPI_ISL_2587402, EPI_ISL_2587404	J. Craig Venter Institute	J. Craig Venter Institute	Shabman,R., Das,S.R., Shilts,M., Fedorova,N., Puri,V., Shrivastava,S., Amedeo,P., Williams,M., Barratt,K., Mitchell,J. and Jennings,L.
EPI_ISL_2587406	J. Craig Venter Institute	J. Craig Venter Institute	Das,S., Halpin,R.A., Bera,J., Puri,V., Fedorova,N., Tsitrin,T., Stockwell,T., Amedeo,P., Bishop,B., Katzel,D., Schobel,S., Shrivastava,S., Hartert,T., Moore,M., Chappell,J., Larkin,E., Wentworth,D.E. and Anderson,L.J.
EPI_ISL_2587408, EPI_ISL_2587410	J. Craig Venter Institute	J. Craig Venter Institute	Shabman,R., Das,S.R., Shilts,M., Fedorova,N., Puri,V., Shrivastava,S., Amedeo,P., Williams,M., Barratt,K., Mitchell,J. and Jennings,L.
EPI_ISL_2587412	J. Craig Venter Institute	J. Craig Venter Institute	Shabman,R., Das,S.R., Shilts,M., Fedorova,N., Puri,V., Shrivastava,S., Amedeo,P., Hu,L., Durbin,A., Rocchi,I., Williams,T. and Hartert,T.
EPI_ISL_2587414, EPI_ISL_2587415	J. Craig Venter Institute	J. Craig Venter Institute	Das,S., Halpin,R.A., Bera,J., Fedorova,N., Tsitrin,T., Stockwell,T., Amedeo,P., Bishop,B., Gupta,N., Hoover,J., Katzel,D., Schobel,S., Shrivastava,S., Hartert,T., Moore,M., Chappell,J., Larkin,E., Wentworth,D.E. and Anderson,L.J.
EPI_ISL_2587417, EPI_ISL_2587421, EPI_ISL_2587423, EPI_ISL_2587425, EPI_ISL_2587429, EPI_ISL_2587431, EPI_ISL_2587433	J. Craig Venter Institute	J. Craig Venter Institute	Das,S.R., Halpin,R.A., Shilts,M., Puri,V., Akopov,A., Fedorova,N., Stockwell,T., Amedeo,P., Bishop,B., Katzel,D., Schobel,S., Shrivastava,S. and Hartert,T.
EPI_ISL_2587434	J. Craig Venter Institute	J. Craig Venter Institute	Das,S., Halpin,R.A., Bera,J., Puri,V., Fedorova,N., Tsitrin,T., Stockwell,T., Amedeo,P., Bishop,B., Katzel,D., Schobel,S., Shrivastava,S., Hartert,T., Moore,M., Chappell,J., Larkin,E., Wentworth,D.E. and Anderson,L.J.
EPI_ISL_2587436, EPI_ISL_2587438	J. Craig Venter Institute	J. Craig Venter Institute	Das,S., Halpin,R.A., Bera,J., Fedorova,N., Tsitrin,T., Stockwell,T., Amedeo,P., Bishop,B., Gupta,N., Hoover,J., Katzel,D., Schobel,S., Shrivastava,S., Hartert,T., Moore,M., Chappell,J., Larkin,E., Wentworth,D.E. and Anderson,L.J.
EPI_ISL_2587440, EPI_ISL_2587442, EPI_ISL_2587444, EPI_ISL_2587446, EPI_ISL_2587447, EPI_ISL_2587451, EPI_ISL_2587452, EPI_ISL_2587454, EPI_ISL_2587456, EPI_ISL_2587458	J. Craig Venter Institute	J. Craig Venter Institute	Das,S.R., Halpin,R.A., Shilts,M., Puri,V., Akopov,A., Fedorova,N., Stockwell,T., Amedeo,P., Bishop,B., Katzel,D., Schobel,S., Shrivastava,S. and Hartert,T.
EPI_ISL_2587469	J. Craig Venter Institute	J. Craig Venter Institute	Das,S., Halpin,R.A., Bera,J., Fedorova,N., Tsitrin,T., Stockwell,T., Amedeo,P., Bishop,B., Gupta,N., Hoover,J., Katzel,D., Schobel,S., Shrivastava,S., Hartert,T., Moore,M., Chappell,J., Larkin,E., Wentworth,D.E. and Anderson,L.J.
EPI_ISL_2587471, EPI_ISL_2587473, EPI_ISL_2587494	J. Craig Venter Institute	J. Craig Venter Institute	Das,S.R., Halpin,R.A., Shilts,M., Puri,V., Akopov,A., Fedorova,N., Stockwell,T., Amedeo,P., Bishop,B., Katzel,D., Schobel,S., Shrivastava,S. and Hartert,T.
EPI_ISL_2588290, EPI_ISL_2588292	J. Craig Venter Institute	J. Craig Venter Institute	Das,S., Halpin,R.A., Bera,J., Fedorova,N., Tsitrin,T., Stockwell,T., Amedeo,P., Bishop,B., Gupta,N., Hoover,J., Katzel,D., Schobel,S., Shrivastava,S., Hartert,T., Moore,M., Chappell,J., Larkin,E., Wentworth,D.E. and Anderson,L.J.
EPI_ISL_2588301, EPI_ISL_2588302, EPI_ISL_2588303	J. Craig Venter Institute	J. Craig Venter Institute	Das,S.R., Halpin,R.A., Shilts,M., Puri,V., Akopov,A., Fedorova,N., Stockwell,T., Amedeo,P., Bishop,B., Katzel,D., Schobel,S., Shrivastava,S. and Hartert,T.
EPI_ISL_2588304	J. Craig Venter Institute	J. Craig Venter Institute	Das,S., Halpin,R.A., Bera,J., Fedorova,N., Tsitrin,T., Stockwell,T., Amedeo,P., Bishop,B., Gupta,N., Hoover,J., Katzel,D., Schobel,S., Shrivastava,S., Hartert,T., Moore,M., Chappell,J., Larkin,E., Wentworth,D.E. and Anderson,L.J.
EPI_ISL_2588305, EPI_ISL_2588306	J. Craig Venter Institute	J. Craig Venter Institute	Das,S.R., Halpin,R.A., Shilts,M., Puri,V., Akopov,A., Fedorova,N., Stockwell,T., Amedeo,P., Bishop,B., Katzel,D., Schobel,S., Shrivastava,S. and Hartert,T.
EPI_ISL_2588347	J. Craig Venter Institute	J. Craig Venter Institute	Das,S., Halpin,R.A., Bera,J., Fedorova,N., Tsitrin,T., Stockwell,T., Amedeo,P., Bishop,B., Gupta,N., Hoover,J., Katzel,D., Schobel,S., Shrivastava,S., Hartert,T., Moore,M., Chappell,J., Larkin,E., Wentworth,D.E. and Anderson,L.J.
EPI_ISL_2588348	J. Craig Venter Institute	J. Craig Venter Institute	Das,S., Halpin,R.A., Bera,J., Puri,V., Fedorova,N., Tsitrin,T., Stockwell,T., Amedeo,P., Bishop,B., Katzel,D., Schobel,S., Shrivastava,S., Hartert,T., Moore,M., Chappell,J., Larkin,E., Wentworth,D.E. and Anderson,L.J.

EPI_ISL_2588349, EPI_ISL_2588350, EPI_ISL_2588351	J. Craig Venter Institute	J. Craig Venter Institute	Das,S., Halpin,R.A., Bera,J., Fedorova,N., Tsitrin,T., Stockwell,T., Amedeo,P., Bishop,B., Gupta,N., Hoover,J., Katzel,D., Schobel,S., Shrivastava,S., Hartert,T., Moore,M., Chappell,J., Larkin,E., Wentworth,D.E. and Anderson,L.J.
EPI_ISL_2588352	J. Craig Venter Institute	J. Craig Venter Institute	Das,S., Halpin,R.A., Bera,J., Puri,V., Fedorova,N., Tsitrin,T., Stockwell,T., Amedeo,P., Bishop,B., Katzel,D., Schobel,S., Shrivastava,S., Hartert,T., Moore,M., Chappell,J., Larkin,E., Wentworth,D.E. and Anderson,L.J.
EPI_ISL_2588353, EPI_ISL_2588354, EPI_ISL_2588355	J. Craig Venter Institute	J. Craig Venter Institute	Das,S.R., Halpin,R.A., Shilts,M., Puri,V., Akopov,A., Fedorova,N., Stockwell,T., Amedeo,P., Bishop,B., Katzel,D., Schobel,S., Shrivastava,S. and Hartert,T.
EPI_ISL_2588356	J. Craig Venter Institute	J. Craig Venter Institute	Shabman,R., Das,S.R., Shilts,M., Fedorova,N., Puri,V., Shrivastava,S., Amedeo,P., Williams,M., Barratt,K., Mitchell,J. and Jennings,L.
EPI_ISL_2588357, EPI_ISL_2588358, EPI_ISL_2588359, EPI_ISL_2588360, EPI_ISL_2588361, EPI_ISL_2588362	J. Craig Venter Institute	J. Craig Venter Institute	Das,S., Halpin,R.A., Bera,J., Fedorova,N., Tsitrin,T., Stockwell,T., Amedeo,P., Bishop,B., Gupta,N., Hoover,J., Katzel,D., Schobel,S., Shrivastava,S., Hartert,T., Moore,M., Chappell,J., Larkin,E., Wentworth,D.E. and Anderson,L.J.
EPI_ISL_2588363, EPI_ISL_2588364, EPI_ISL_2588365, EPI_ISL_2588366, EPI_ISL_2588367, EPI_ISL_2588368, EPI_ISL_2588369, EPI_ISL_2588370, EPI_ISL_2588371, EPI_ISL_2588372, EPI_ISL_2588373, EPI_ISL_2588374, EPI_ISL_2588375			
see above	J. Craig Venter Institute	J. Craig Venter Institute	Das,S.R., Halpin,R.A., Shilts,M., Puri,V., Akopov,A., Fedorova,N., Stockwell,T., Amedeo,P., Bishop,B., Katzel,D., Schobel,S., Shrivastava,S. and Hartert,T.
EPI_ISL_2588376	J. Craig Venter Institute	J. Craig Venter Institute	Shabman,R., Das,S.R., Puri,V., Fedorova,N., Amedeo,P., Williams,M., Shrivastava,S. and Halasa,N.
EPI_ISL_2588377, EPI_ISL_2588378	J. Craig Venter Institute	J. Craig Venter Institute	Das,S.R., Halpin,R.A., Shilts,M., Puri,V., Akopov,A., Fedorova,N., Stockwell,T., Amedeo,P., Bishop,B., Katzel,D., Schobel,S., Shrivastava,S. and Hartert,T.
EPI_ISL_2588379	J. Craig Venter Institute	J. Craig Venter Institute	Wentworth,D.E., Halpin,R.A., Bera,J., Lin,X., Fedorova,N., Tsitrin,T., McLellan,M., Stockwell,T., Amedeo,P., Bishop,B., Gupta,N., Hoover,J., Katzel,D., Schobel,S., Shrivastava,S., Garcia,J., Laguna-Torres,V.A., Leguia,M., Benavides,J.G. and Halsey,E.
EPI_ISL_2588380	J. Craig Venter Institute	J. Craig Venter Institute	Das,S.R., Halpin,R.A., Shilts,M., Puri,V., Akopov,A., Fedorova,N., Stockwell,T., Amedeo,P., Bishop,B., Katzel,D., Schobel,S., Shrivastava,S. and Hartert,T.
EPI_ISL_2588437	J. Craig Venter Institute	J. Craig Venter Institute	Shabman,R., Das,S.R., Puri,V., Fedorova,N., Amedeo,P., Williams,M., Shrivastava,S. and Halasa,N.
EPI_ISL_2588597	J. Craig Venter Institute	J. Craig Venter Institute	Das,S., Halpin,R.A., Bera,J., Fedorova,N., Tsitrin,T., Stockwell,T., Amedeo,P., Bishop,B., Gupta,N., Hoover,J., Katzel,D., Schobel,S., Shrivastava,S., Hartert,T., Moore,M., Chappell,J., Larkin,E., Wentworth,D.E. and Anderson,L.J.
EPI_ISL_2588598, EPI_ISL_2588599, EPI_ISL_2588600	J. Craig Venter Institute	J. Craig Venter Institute	Das,S.R., Halpin,R.A., Shilts,M., Puri,V., Akopov,A., Fedorova,N., Stockwell,T., Amedeo,P., Bishop,B., Katzel,D., Schobel,S., Shrivastava,S. and Hartert,T.
EPI_ISL_2588651	Virology Laboratory, Dr. Ricardo Gutierrez Children Hospital	Virology Laboratory, Dr. Ricardo Gutierrez Children Hospital	Goya,S., Rojo,G.L., Valinotto,L.E., Mistchenko,A.S. and Viegas,M.
EPI_ISL_2588653	J. Craig Venter Institute	J. Craig Venter Institute	Lorenzi,H., Town,C., Halpin,R., Bera,J., Ransier,A., Fedorova,N., Stockwell,T., Amedeo,P., Appalla,L., Bishop,B., Edworthy,P., Gupta,N., Hoover,J., Katzel,D., Li,K., Schobel,S., Shrivastava,S., Thovarai,V., Wang,S., Rebuffo-Scheer,C., Fan,J., He,J., Kehl,S.C., Lederboer,N., Jurgens,L.A., Bose,M.E., Beck,E.T., Kumar,S., Gerna,G., Wentworth,D.E. and Henrickson,K.J.
EPI_ISL_2588654	Virology Laboratory, Dr. Ricardo Gutierrez Children Hospital	Virology Laboratory, Dr. Ricardo Gutierrez Children Hospital	Goya,S., Valinotto,L.E., Tittarelli,E., Rojo,G.L., Greninger,A., Luso,S., Natale,M., Mistchenko,A.S. and Viegas,M.
EPI_ISL_2588655	Virology Laboratory, Dr. Ricardo Gutierrez Children Hospital	Virology Laboratory, Dr. Ricardo Gutierrez Children Hospital	Goya,S., Rojo,G.L., Valinotto,L.E., Mistchenko,A.S. and Viegas,M.
EPI_ISL_2588656, EPI_ISL_2588657, EPI_ISL_2588658	J. Craig Venter Institute	J. Craig Venter Institute	Lorenzi,H., Town,C., Halpin,R., Bera,J., Ransier,A., Fedorova,N., Stockwell,T., Amedeo,P., Appalla,L., Bishop,B., Edworthy,P., Gupta,N., Hoover,J., Katzel,D., Li,K., Schobel,S., Shrivastava,S., Thovarai,V., Wang,S., Rebuffo-Scheer,C., Fan,J., He,J., Kehl,S.C., Lederboer,N., Jurgens,L.A., Bose,M.E., Beck,E.T., Kumar,S., Gerna,G., Wentworth,D.E. and Henrickson,K.J.
EPI_ISL_2588659	Key Laboratory of Emergency Detection for Public Health of Zhejiang Province, Zhejiang Provincial Centre for Disease Control and Prevention	Key Laboratory of Emergency Detection for Public Health of Zhejiang Province, Zhejiang Provincial Centre for Disease Control and Prevention	Li,C.-X., Li,W., Zhou,J., Zhang,B., Feng,Y., Xu,C.-P., Lu,Y.-Y., Holmes,E.C. and Shi,M.
EPI_ISL_2588660	Medical Microbiology, University Medical Center Utrecht	Medical Microbiology, University Medical Center Utrecht	Tan,L., Viveen,M.C., Lemey,P. and Coenjaerts,F.E.
EPI_ISL_2588661	J. Craig Venter Institute	J. Craig Venter Institute	Lorenzi,H., Town,C., Halpin,R., Bera,J., Ransier,A., Fedorova,N., Stockwell,T., Amedeo,P., Appalla,L., Bishop,B., Edworthy,P., Gupta,N., Hoover,J., Katzel,D., Schobel,S., Shrivastava,S., Thovarai,V., Wang,S., Rebuffo-Scheer,C., Fan,J., He,J., Kehl,S.C., Lederboer,N., Jurgens,L.A., Bose,M.E., Beck,E.T., Kumar,S., Gerna,G., Wentworth,D.E. and Henrickson,K.J.
EPI_ISL_2588662	Medical Microbiology, University Medical Center Utrecht	Medical Microbiology, University Medical Center Utrecht	Tan,L., Viveen,M.C., Lemey,P. and Coenjaerts,F.E.
EPI_ISL_2588663	Microbiology, Institute of Biological Sciences, Universeity of Sao Paulo	Microbiology, Institute of Biological Sciences, Universeity of Sao Paulo	Di Paola,N., Cunha,M.P., Oliveira,D.B.L., Durigon,E., Durigon,G.S. and Zanotto,P.M.A.
EPI_ISL_2588664	Key Laboratory of Emergency Detection for Public Health of Zhejiang Province, Zhejiang Provincial Centre for Disease Control and Prevention	Key Laboratory of Emergency Detection for Public Health of Zhejiang Province, Zhejiang Provincial Centre for Disease Control and Prevention	Li,C.-X., Li,W., Zhou,J., Zhang,B., Feng,Y., Xu,C.-P., Lu,Y.-Y., Holmes,E.C. and Shi,M.
EPI_ISL_2588666	Virology Laboratory, Dr. Ricardo Gutierrez Children Hospital	Virology Laboratory, Dr. Ricardo Gutierrez Children Hospital	Goya,S., Valinotto,L.E., Tittarelli,E., Rojo,G.L., Greninger,A., Luso,S., Natale,M., Mistchenko,A.S. and Viegas,M.
EPI_ISL_2588667	Mami Nagashima Tokyo Metropolitan Institute of Public Health, Microbiology	Mami Nagashima Tokyo Metropolitan Institute of Public Health, Microbiology	Hasegawa,M., Okazaki,T., Sakamoto,T., Murata,R., Nagashima,M., Shinkai,T. and Sadamasu,K.
EPI_ISL_2588668	J. Craig Venter Institute	J. Craig Venter Institute	Lorenzi,H., Town,C., Halpin,R., Bera,J., Ransier,A., Fedorova,N., Stockwell,T., Amedeo,P., Appalla,L., Bishop,B., Edworthy,P., Gupta,N., Hoover,J., Katzel,D., Li,K., Schobel,S., Shrivastava,S., Thovarai,V., Wang,S., Rebuffo-Scheer,C., Fan,J., He,J., Kehl,S.C., Lederboer,N., Jurgens,L.A., Bose,M.E., Beck,E.T., Kumar,S., Gerna,G., Wentworth,D.E. and Henrickson,K.J.
EPI_ISL_2588669	Key Laboratory of Emergency Detection for Public Health of Zhejiang Province, Zhejiang Provincial Centre for Disease Control and Prevention	Key Laboratory of Emergency Detection for Public Health of Zhejiang Province, Zhejiang Provincial Centre for Disease Control and Prevention	Li,C.-X., Li,W., Zhou,J., Zhang,B., Feng,Y., Xu,C.-P., Lu,Y.-Y., Holmes,E.C. and Shi,M.
EPI_ISL_2588684	J. Craig Venter Institute	J. Craig Venter Institute	Lorenzi,H., Town,C., Halpin,R., Bera,J., Ransier,A., Fedorova,N., Stockwell,T., Amedeo,P., Appalla,L., Bishop,B., Edworthy,P., Gupta,N., Hoover,J., Katzel,D., Li,K., Schobel,S., Shrivastava,S., Thovarai,V., Wang,S., Rebuffo-Scheer,C., Fan,J., He,J., Kehl,S.C., Lederboer,N., Jurgens,L.A., Bose,M.E., Beck,E.T., Kumar,S., Gerna,G., Wentworth,D.E. and Henrickson,K.J.
EPI_ISL_2588685	J. Craig Venter Institute	J. Craig Venter Institute	Lorenzi,H., Town,C., Halpin,R., Bera,J., Ransier,A., Fedorova,N., Stockwell,T., Amedeo,P., Appalla,L., Bishop,B., Edworthy,P., Gupta,N., Hoover,J., Katzel,D., Li,K., Schobel,S., Shrivastava,S., Thovarai,V., Wang,S., Rebuffo-Scheer,C., Fan,J., He,J., Kehl,S.C., Lederboer,N., Jurgens,L.A., Bose,M.E., Beck,E.T., Kumar,S., Videla,C., Wentworth,D.E. and Henrickson,K.J.
EPI_ISL_2588688	J. Craig Venter Institute	J. Craig Venter Institute	Lorenzi,H., Town,C., Halpin,R., Bera,J., Ransier,A., Fedorova,N., Stockwell,T., Amedeo,P., Appalla,L., Bishop,B., Edworthy,P., Gupta,N., Hoover,J., Katzel,D., Schobel,S., Shrivastava,S., Thovarai,V., Wang,S., Rebuffo-Scheer,C., Fan,J., He,J., Kehl,S.C., Lederboer,N., Jurgens,L.A., Bose,M.E., Beck,E.T., Kumar,S., Neumann-Haefelin,D., Wentworth,D.E. and Henrickson,K.J.
EPI_ISL_2588720	Virology Laboratory, Dr. Ricardo Gutierrez Children Hospital	Virology Laboratory, Dr. Ricardo Gutierrez Children Hospital	Goya,S., Valinotto,L.E., Tittarelli,E., Rojo,G.L., Greninger,A., Luso,S., Natale,M., Mistchenko,A.S. and Viegas,M.
EPI_ISL_2588721, EPI_ISL_2588722	Virology Laboratory, Dr. Ricardo Gutierrez Children Hospital	Virology Laboratory, Dr. Ricardo Gutierrez Children Hospital	Goya,S., Valinotto,L.E., Tittarelli,E., Rojo,G.L., Greninger,A., Zaiat,J., Marti,M., Mistchenko,A.S. and Viegas,M.
EPI_ISL_2588782	J. Craig Venter Institute	J. Craig Venter Institute	Lorenzi,H., Town,C., Halpin,R., Bera,J., Ransier,A., Fedorova,N., Stockwell,T., Amedeo,P., Appalla,L., Bishop,B., Edworthy,P., Gupta,N., Hoover,J., Katzel,D., Li,K., Schobel,S., Shrivastava,S., Thovarai,V., Wang,S., Rebuffo-Scheer,C., Fan,J., He,J., Kehl,S.C., Lederboer,N., Jurgens,L.A., Bose,M.E., Beck,E.T., Kumar,S., Gerna,G., Wentworth,D.E. and Henrickson,K.J.
EPI_ISL_2588785	Mami Nagashima Tokyo Metropolitan Institute of Public Health, Microbiology	Mami Nagashima Tokyo Metropolitan Institute of Public Health, Microbiology	Hasegawa,M., Okazaki,T., Sakamoto,T., Murata,R., Nagashima,M., Shinkai,T. and Sadamasu,K.
EPI_ISL_2588794	Virology Laboratory, Dr. Ricardo Gutierrez Children Hospital	Virology Laboratory, Dr. Ricardo Gutierrez Children Hospital	Goya,S., Rojo,G.L., Valinotto,L.E., Mistchenko,A.S. and Viegas,M.

EPI_ISL_2588795	Virology Laboratory, Dr. Ricardo Gutierrez Children Hospital	Virology Laboratory, Dr. Ricardo Gutierrez Children Hospital	Goya,S., Valinotto,L.E., Tittarelli,E., Rojo,G.L., Greninger,A., Zaiat,J., Marti,M., Mistchenko,A.S. and Viegas,M.
EPI_ISL_2588796, EPI_ISL_2588818	Virology Laboratory, Dr. Ricardo Gutierrez Children Hospital	Virology Laboratory, Dr. Ricardo Gutierrez Children Hospital	Goya,S., Valinotto,L.E., Tittarelli,E., Rojo,G.L., Greninger,A., Luso,S., Natale,M., Mistchenko,A.S. and Viegas,M.
EPI_ISL_2588827	Virology Laboratory, Dr. Ricardo Gutierrez Children Hospital	Virology Laboratory, Dr. Ricardo Gutierrez Children Hospital	Goya,S., Valinotto,L.E., Tittarelli,E., Rojo,G.L., Greninger,A., Zaiat,J., Marti,M., Mistchenko,A.S. and Viegas,M.
EPI_ISL_2588828	Virology Laboratory, Dr. Ricardo Gutierrez Children Hospital	Virology Laboratory, Dr. Ricardo Gutierrez Children Hospital	Goya,S., Rojo,G.L., Valinotto,L.E., Mistchenko,A.S. and Viegas,M.
EPI_ISL_2592492	J. Craig Venter Institute	J. Craig Venter Institute	Shabman,R., Fedorova,N., Puri,V., Shrivastava,S., Amedeo,P., Isom,R., Hu,L., Pickett,B., Novotny,M., Durbin,A., Rocchi,I., Williams,T., Hall,C.B., Tesini,B.L., Schnabel,K.C., Walsh,E.E. and Caserta,M.
EPI_ISL_2592529	Pediatrics - Infectious Diseases, Medical College of Wisconsin	Pediatrics - Infectious Diseases, Medical College of Wisconsin	Rebuffo-Scheer,C., Bose,M.E., He,J., Khaja,S., Ulatowski,M., Beck,E.T., Fan,J., Kumar,S., Nelson,M.I. and Henrickson,K.J.
EPI_ISL_2592567, EPI_ISL_2592568, EPI_ISL_2592569, EPI_ISL_2592570, EPI_ISL_2592571, EPI_ISL_2592572	Broad Institute of MIT & Harvard	Broad Institute of MIT & Harvard	Newman,R.M., Zody,M.C., DeVincenzo,J.P., Grad,Y., Lipsitch,M., Murphy,R., Fitzgerald,M., Young,S., Gargeya,S., Poon,T.W., Charlebois,P., Weiner,B., Yang,X., Piper,M.E., McCowan,C., Ireland,A., Levin,J., Malboeuf,C., Qu,J., Chapman,S.B., Murphy,C., Wortman,J., Nusbaum,C. and Birren,B.
EPI_ISL_2592783	Pediatrics - Infectious Diseases, Medical College of Wisconsin	Pediatrics - Infectious Diseases, Medical College of Wisconsin	Rebuffo-Scheer,C., Bose,M.E., He,J., Khaja,S., Ulatowski,M., Beck,E.T., Fan,J., Kumar,S., Nelson,M.I. and Henrickson,K.J.
EPI_ISL_2593173	Virology, University of Washington	Virology, University of Washington	Greninger,A.L., Shean,R.C. and Makhsous,N.
EPI_ISL_2594878, EPI_ISL_2594879, EPI_ISL_2595159, EPI_ISL_2595160	Center for Infectious Diseases, School of Public Health, University of Texas Health Science Center	Center for Infectious Diseases, School of Public Health, University of Texas Health Science Center	Bahl,J., Hixson,J., Kim,D.-K., Qiu,X., Piedra,P.A., Piedra,F.-A., Avadhanula,V. and Machado,A.A.
EPI_ISL_2595173	Beijing Key Laboratory of Etiology of Viral Diseases in Children; Laboratory of Virology, Capital Institute of Pediatrics	Beijing Key Laboratory of Etiology of Viral Diseases in Children; Laboratory of Virology, Capital Institute of Pediatrics	Cui,G., Zhu,R., Deng,J., Zhao,L., Sun,Y., Wang,F. and Qian,Y.
EPI_ISL_2595181, EPI_ISL_2595182	Center for Infectious Diseases, School of Public Health, University of Texas Health Science Center	Center for Infectious Diseases, School of Public Health, University of Texas Health Science Center	Bahl,J., Hixson,J., Kim,D.-K., Qiu,X., Piedra,P.A., Piedra,F.-A., Avadhanula,V. and Machado,A.A.
EPI_ISL_2595188, EPI_ISL_2595192, EPI_ISL_2595196, EPI_ISL_2595197, EPI_ISL_2595198, EPI_ISL_2595205	Epidemiology and Demography Department, KEMRI-Wellcome Trust Research Programme	Epidemiology and Demography Department, KEMRI-Wellcome Trust Research Programme	Otieno,J.R., Kamau,E.M., Oketch,J.W., Ngoi,J.M., Agoti,C.N., Gichuki,A.M., Otieno,G.P., Ngama,M., Cane,P.A., Kellam,P., Cotten,M., Lemey,P. and Nokes,D.J.
EPI_ISL_2595236, EPI_ISL_2595277, EPI_ISL_2595278, EPI_ISL_2595279	Broad Institute of MIT & Harvard	Broad Institute of MIT & Harvard	Newman,R.M., Zody,M.C., DeVincenzo,J.P., Grad,Y., Lipsitch,M., Murphy,R., Fitzgerald,M., Young,S., Gargeya,S., Poon,T.W., Charlebois,P., Weiner,B., Yang,X., Piper,M.E., McCowan,C., Ireland,A., Levin,J., Malboeuf,C., Qu,J., Chapman,S.B., Murphy,C., Wortman,J., Nusbaum,C. and Birren,B.
EPI_ISL_2595315	Epidemiology and Demography Department, KEMRI-Wellcome Trust Research Programme	Epidemiology and Demography Department, KEMRI-Wellcome Trust Research Programme	Otieno,J.R., Kamau,E.M., Oketch,J.W., Ngoi,J.M., Agoti,C.N., Gichuki,A.M., Otieno,G.P., Ngama,M., Cane,P.A., Kellam,P., Cotten,M., Lemey,P. and Nokes,D.J.
EPI_ISL_2595321	J. Craig Venter Institute	J. Craig Venter Institute	Shrivastava,S., Halpin,R.A., Puri,V., Fedorova,N.B., Stockwell,T., Amedeo,P., Katzel,D., Schobel,S., Pickett,B.E., Moore,M., Chappell,J., Larkin,E., Wentworth,D.E., Anderson,L.J. and Hartert,T.
EPI_ISL_2595357	Epidemiology and Demography Department, KEMRI-Wellcome Trust Research Programme	Epidemiology and Demography Department, KEMRI-Wellcome Trust Research Programme	Otieno,J.R., Kamau,E.M., Oketch,J.W., Ngoi,J.M., Agoti,C.N., Gichuki,A.M., Otieno,G.P., Ngama,M., Cane,P.A., Kellam,P., Cotten,M., Lemey,P. and Nokes,D.J.
EPI_ISL_2595477	Marie Bashir Institute for Infectious Diseases and Biosecurity & Sydney Medical School, The University of Sydney, Westmead Institute for Medical Research	Marie Bashir Institute for Infectious Diseases and Biosecurity & Sydney Medical School, The University of Sydney, Westmead Institute for Medical Research	Eden,J.-S., Kok,J., Dwyer,D.E., Fernandez,M., Carter,I. and Holmes,E.C.
EPI_ISL_2595540, EPI_ISL_2595541	Epidemiology and Demography Department, KEMRI-Wellcome Trust Research Programme	Epidemiology and Demography Department, KEMRI-Wellcome Trust Research Programme	Otieno,J.R., Kamau,E.M., Oketch,J.W., Ngoi,J.M., Agoti,C.N., Gichuki,A.M., Otieno,G.P., Ngama,M., Cane,P.A., Kellam,P., Cotten,M., Lemey,P. and Nokes,D.J.
EPI_ISL_2595546	Department of Pediatrics, Center of Excellence in Clinical Virology, Chulalongkorn	Department of Pediatrics, Center of Excellence in Clinical Virology, Chulalongkorn	Thongpan,I.
EPI_ISL_2595548, EPI_ISL_2595550	Epidemiology and Demography Department, KEMRI-Wellcome Trust Research Programme	Epidemiology and Demography Department, KEMRI-Wellcome Trust Research Programme	Otieno,J.R., Kamau,E.M., Oketch,J.W., Ngoi,J.M., Agoti,C.N., Gichuki,A.M., Otieno,G.P., Ngama,M., Cane,P.A., Kellam,P., Cotten,M., Lemey,P. and Nokes,D.J.
EPI_ISL_2595591, EPI_ISL_2595592, EPI_ISL_2595604	Kazuya Shirato National Institute of Infectious Diseases, Virology III	Kazuya Shirato National Institute of Infectious Diseases, Virology III	Shirato,K., Sato,K., Dapat,I., Nao,N., Omiya,S., Matsuyama,S., Takeda,M. and Nishimura,H.
EPI_ISL_2595605, EPI_ISL_2595607, EPI_ISL_2595608, EPI_ISL_2595609	Marie Bashir Institute for Infectious Diseases and Biosecurity & Sydney Medical School, The University of Sydney, Westmead Institute for Medical Research	Marie Bashir Institute for Infectious Diseases and Biosecurity & Sydney Medical School, The University of Sydney, Westmead Institute for Medical Research	Eden,J.-S., Kok,J., Dwyer,D.E., Fernandez,M., Carter,I. and Holmes,E.C.
EPI_ISL_2595626, EPI_ISL_2595627, EPI_ISL_2595628, EPI_ISL_2595631, EPI_ISL_2595633, EPI_ISL_2595634, EPI_ISL_2595636, EPI_ISL_2595638, EPI_ISL_2595639, EPI_ISL_2595640, EPI_ISL_2595641, EPI_ISL_2595642, EPI_ISL_2595644			
see above	Epidemiology and Demography Department, KEMRI-Wellcome Trust Research Programme	Epidemiology and Demography Department, KEMRI-Wellcome Trust Research Programme	Otieno,J.R., Kamau,E.M., Oketch,J.W., Ngoi,J.M., Agoti,C.N., Gichuki,A.M., Otieno,G.P., Ngama,M., Cane,P.A., Kellam,P., Cotten,M., Lemey,P. and Nokes,D.J.
EPI_ISL_2595651	Lab Medicine, UW	Lab Medicine, UW	Greninger,A.L., Makhsous,N., Kuypers,J.M., Shean,R.C. and Jerome,K.R.
EPI_ISL_2595653, EPI_ISL_2595654, EPI_ISL_2595655, EPI_ISL_2595657, EPI_ISL_2595658, EPI_ISL_2595660, EPI_ISL_2595661, EPI_ISL_2595663, EPI_ISL_2595665, EPI_ISL_2595672, EPI_ISL_2595673			
see above	Epidemiology and Demography Department, KEMRI-Wellcome Trust Research Programme	Epidemiology and Demography Department, KEMRI-Wellcome Trust Research Programme	Otieno,J.R., Kamau,E.M., Oketch,J.W., Ngoi,J.M., Agoti,C.N., Gichuki,A.M., Otieno,G.P., Ngama,M., Cane,P.A., Kellam,P., Cotten,M., Lemey,P. and Nokes,D.J.
EPI_ISL_2595681, EPI_ISL_2595684	Marie Bashir Institute for Infectious Diseases and Biosecurity & Sydney Medical School, The University of Sydney, Westmead Institute for Medical Research	Marie Bashir Institute for Infectious Diseases and Biosecurity & Sydney Medical School, The University of Sydney, Westmead Institute for Medical Research	Eden,J.-S., Kok,J., Dwyer,D.E., Fernandez,M., Carter,I. and Holmes,E.C.
EPI_ISL_2595691	J. Craig Venter Institute	J. Craig Venter Institute	Tan,G., Pickett,B., Fedorova,N., Amedeo,P., Hu,L., Christensen,J., Miller,J., Durbin,A., Williams,T., Arumemi,F., Cadiz,C., Alanis,R., Balmseda,A., Wiliams,T., Schiller,A., Patel,M., Kubale,J. and Gordon,A.
EPI_ISL_2595696	Pediatrics, University of New Mexico	Pediatrics, University of New Mexico	Kothari,A., Kennedy,J.L., Schwalm,K.C., Putt,C., Denson,J.L. and Dinwiddie,D.L.
EPI_ISL_2595697	Epidemiology and Demography Department, KEMRI-Wellcome Trust Research Programme	Epidemiology and Demography Department, KEMRI-Wellcome Trust Research Programme	Otieno,J.R., Kamau,E.M., Oketch,J.W., Ngoi,J.M., Agoti,C.N., Gichuki,A.M., Otieno,G.P., Ngama,M., Cane,P.A., Kellam,P., Cotten,M., Lemey,P. and Nokes,D.J.
EPI_ISL_2595699, EPI_ISL_2595700, EPI_ISL_2595701	Pediatrics, University of New Mexico	Pediatrics, University of New Mexico	Kothari,A., Kennedy,J.L., Schwalm,K.C., Putt,C., Denson,J.L. and Dinwiddie,D.L.
EPI_ISL_2595702	Epidemiology and Demography Department, KEMRI-Wellcome Trust Research Programme	Epidemiology and Demography Department, KEMRI-Wellcome Trust Research Programme	Otieno,J.R., Kamau,E.M., Oketch,J.W., Ngoi,J.M., Agoti,C.N., Gichuki,A.M., Otieno,G.P., Ngama,M., Cane,P.A., Kellam,P., Cotten,M., Lemey,P. and Nokes,D.J.
EPI_ISL_2595703, EPI_ISL_2595704, EPI_ISL_2595705	Pediatrics, University of New Mexico	Pediatrics, University of New Mexico	Kothari,A., Kennedy,J.L., Schwalm,K.C., Putt,C., Denson,J.L. and Dinwiddie,D.L.
EPI_ISL_2595706, EPI_ISL_2595707, EPI_ISL_2595708, EPI_ISL_2595709, EPI_ISL_2595710, EPI_ISL_2595711, EPI_ISL_2595712	Marie Bashir Institute for Infectious Diseases and Biosecurity & Sydney Medical School, The University of Sydney, Westmead Institute for Medical Research	Marie Bashir Institute for Infectious Diseases and Biosecurity & Sydney Medical School, The University of Sydney, Westmead Institute for Medical Research	Eden,J.-S., Kok,J., Dwyer,D.E., Fernandez,M., Carter,I. and Holmes,E.C.
EPI_ISL_2595713	Department of Pediatrics, Center of Excellence in Clinical	Department of Pediatrics, Center of Excellence in Clinical	Thongpan,I.

	Virology, Chulalongkorn	Virology, Chulalongkorn	
EPI_ISL_2595714, EPI_ISL_2595715, EPI_ISL_2595716	Marie Bashir Institute for Infectious Diseases and Biosecurity & Sydney Medical School, The University of Sydney, Westmead Institute for Medical Research	Marie Bashir Institute for Infectious Diseases and Biosecurity & Sydney Medical School, The University of Sydney, Westmead Institute for Medical Research	Eden,J.-S., Kok,J., Dwyer,D.E., Fernandez,M., Carter,I. and Holmes,E.C.
EPI_ISL_2595717	J. Craig Venter Institute	J. Craig Venter Institute	Tan,G., Pickett,B., Fedorova,N., Amedeo,P., Hu,L., Christensen,J., Miller,J., Durbin,A., Williams,T., Arumemi,F., Cadiz,C., Alanis,R., Balmseda,A., Wiliams,T., Schiller,A., Patel,M., Kubale,J. and Gordon,A.
EPI_ISL_2595718, EPI_ISL_2595719	Marie Bashir Institute for Infectious Diseases and Biosecurity & Sydney Medical School, The University of Sydney, Westmead Institute for Medical Research	Marie Bashir Institute for Infectious Diseases and Biosecurity & Sydney Medical School, The University of Sydney, Westmead Institute for Medical Research	Eden,J.-S., Kok,J., Dwyer,D.E., Fernandez,M., Carter,I. and Holmes,E.C.
EPI_ISL_2595722	Department of Pediatrics, Center of Excellence in Clinical Virology, Chulalongkorn	Department of Pediatrics, Center of Excellence in Clinical Virology, Chulalongkorn	Thongpan,I.
EPI_ISL_2595723	Gansu Center for Disease Control and Prevention, Pathogen Laboratory	Gansu Center for Disease Control and Prevention, Pathogen Laboratory	Qiao,R., Chen,J., Wu,H. and Yu,D.
EPI_ISL_2595724	Laboratory Medicine, UW Virology	Laboratory Medicine, UW Virology	Lin,M.J., Tait,A. and Greninger,A.L.
EPI_ISL_2811729	Royal Children's Hospital	WHO Collaborating Centre for Reference and Research on Influenza	Jean Moselen, Annette Alafaci, Yi-Mo Deng, Ammar Aziz, Naomi Komadina
EPI_ISL_2835616	PathWest Laboratory Medicine WA Microbial Surveillance Unit	PathWest Laboratory Medicine WA Microbial Surveillance Unit	Chisha Sikazwe, Avram Levy, David Smith, Chris Blyth, Alice Michie, Cara Minney-Smith, David Speers
EPI_ISL_2839170, EPI_ISL_2839171, EPI_ISL_2839172, EPI_ISL_2839173, EPI_ISL_2839176, EPI_ISL_2839177	PathWest Laboratory Medicine WA Microbial Surveillance Unit	PathWest Laboratory Medicine WA Microbial Surveillance Unit	"Chisha Sikazwe, Avram Levy, David Smith, Chris Blyth, Alice Michie, Cara Minney-Smith, David Speers"
EPI_ISL_2839185, EPI_ISL_2839186, EPI_ISL_2839187, EPI_ISL_2839188, EPI_ISL_2839189	Centre for Infectious Diseases and Microbiology Laboratory Services	Centre for Infectious Diseases and Microbiology Laboratory Services	"John-Sebastian Eden, Jen Kok, Dominic Dwyer, Edward Holmes, Philip Britton, Alison Kesson, Elena Cutmore, Rachel Tulloch, Bethany Horsburgh"
EPI_ISL_2839190	Departments of Clinical Microbiology and Infectious Diseases	Centre for Infectious Diseases and Microbiology Laboratory Services	"John-Sebastian Eden, Jen Kok, Dominic Dwyer, Edward Holmes, Philip Britton, Alison Kesson, Elena Cutmore, Rachel Tulloch, Bethany Horsburgh"
EPI_ISL_2839196	Centre for Infectious Diseases and Microbiology Laboratory Services	Centre for Infectious Diseases and Microbiology Laboratory Services	"John-Sebastian Eden, Jen Kok, Dominic Dwyer, Edward Holmes, Philip Britton, Alison Kesson, Elena Cutmore, Rachel Tulloch, Bethany Horsburgh"
EPI_ISL_2839197	Departments of Clinical Microbiology and Infectious Diseases	Centre for Infectious Diseases and Microbiology Laboratory Services	"John-Sebastian Eden, Jen Kok, Dominic Dwyer, Edward Holmes, Philip Britton, Alison Kesson, Elena Cutmore, Rachel Tulloch, Bethany Horsburgh"
EPI_ISL_2839198	Centre for Infectious Diseases and Microbiology Laboratory Services	Centre for Infectious Diseases and Microbiology Laboratory Services	"John-Sebastian Eden, Jen Kok, Dominic Dwyer, Edward Holmes, Philip Britton, Alison Kesson, Elena Cutmore, Rachel Tulloch, Bethany Horsburgh"
EPI_ISL_2839199	PathWest Laboratory Medicine WA Microbial Surveillance Unit	PathWest Laboratory Medicine WA Microbial Surveillance Unit	"Chisha Sikazwe, Avram Levy, David Smith, Chris Blyth, Alice Michie, Cara Minney-Smith, David Speers"
EPI_ISL_2839201, EPI_ISL_2839202, EPI_ISL_2839203, EPI_ISL_2839204, EPI_ISL_2839205, EPI_ISL_2839206, EPI_ISL_2839207, EPI_ISL_2839208, EPI_ISL_2839209, EPI_ISL_2839210, EPI_ISL_2839211			
see above	Centre for Infectious Diseases and Microbiology Laboratory Services	Centre for Infectious Diseases and Microbiology Laboratory Services	"John-Sebastian Eden, Jen Kok, Dominic Dwyer, Edward Holmes, Philip Britton, Alison Kesson, Elena Cutmore, Rachel Tulloch, Bethany Horsburgh"
EPI_ISL_2839213, EPI_ISL_2839214, EPI_ISL_2839215, EPI_ISL_2839216, EPI_ISL_2839217, EPI_ISL_2839218	Departments of Clinical Microbiology and Infectious Diseases	Centre for Infectious Diseases and Microbiology Laboratory Services	"John-Sebastian Eden, Jen Kok, Dominic Dwyer, Edward Holmes, Philip Britton, Alison Kesson, Elena Cutmore, Rachel Tulloch, Bethany Horsburgh"
EPI_ISL_2839219, EPI_ISL_2839220, EPI_ISL_2839221, EPI_ISL_2839222, EPI_ISL_2839223, EPI_ISL_2839224, EPI_ISL_2839225	Centre for Infectious Diseases and Microbiology Laboratory Services	Centre for Infectious Diseases and Microbiology Laboratory Services	"John-Sebastian Eden, Jen Kok, Dominic Dwyer, Edward Holmes, Philip Britton, Alison Kesson, Elena Cutmore, Rachel Tulloch, Bethany Horsburgh"
EPI_ISL_2839227, EPI_ISL_2839228, EPI_ISL_2839229, EPI_ISL_2839230, EPI_ISL_2839231, EPI_ISL_2839232, EPI_ISL_2839233, EPI_ISL_2839234	Departments of Clinical Microbiology and Infectious Diseases	Centre for Infectious Diseases and Microbiology Laboratory Services	"John-Sebastian Eden, Jen Kok, Dominic Dwyer, Edward Holmes, Philip Britton, Alison Kesson, Elena Cutmore, Rachel Tulloch, Bethany Horsburgh"
EPI_ISL_2839235, EPI_ISL_2839236, EPI_ISL_2839237, EPI_ISL_2839239, EPI_ISL_2839242, EPI_ISL_2839244, EPI_ISL_2839246, EPI_ISL_2839248, EPI_ISL_2839250, EPI_ISL_2839252, EPI_ISL_2839254, EPI_ISL_2839256, EPI_ISL_2839258, EPI_ISL_2839260, EPI_ISL_2839262, EPI_ISL_2839264, EPI_ISL_2839266, EPI_ISL_2839269, EPI_ISL_2839271, EPI_ISL_2839273, EPI_ISL_2839275, EPI_ISL_2839277, EPI_ISL_2839279, EPI_ISL_2839281, EPI_ISL_2839283, EPI_ISL_2839285, EPI_ISL_2839288, EPI_ISL_2839290, EPI_ISL_2839292			
see above	Centre for Infectious Diseases and Microbiology Laboratory Services	Centre for Infectious Diseases and Microbiology Laboratory Services	"John-Sebastian Eden, Jen Kok, Dominic Dwyer, Edward Holmes, Philip Britton, Alison Kesson, Elena Cutmore, Rachel Tulloch, Bethany Horsburgh"
EPI_ISL_2839294, EPI_ISL_2839296, EPI_ISL_2839298, EPI_ISL_2839300, EPI_ISL_2839302, EPI_ISL_2839304, EPI_ISL_2839306, EPI_ISL_2839308, EPI_ISL_2839310, EPI_ISL_2839312	Departments of Clinical Microbiology and Infectious Diseases	Centre for Infectious Diseases and Microbiology Laboratory Services	"John-Sebastian Eden, Jen Kok, Dominic Dwyer, Edward Holmes, Philip Britton, Alison Kesson, Elena Cutmore, Rachel Tulloch, Bethany Horsburgh"
EPI_ISL_2839313, EPI_ISL_2839316, EPI_ISL_2839318, EPI_ISL_2839320, EPI_ISL_2839322, EPI_ISL_2839324, EPI_ISL_2839326, EPI_ISL_2839328, EPI_ISL_2839330, EPI_ISL_2839332, EPI_ISL_2839334, EPI_ISL_2839337, EPI_ISL_2839339, EPI_ISL_2839341, EPI_ISL_2839343, EPI_ISL_2839345, EPI_ISL_2839347, EPI_ISL_2839349			
see above	Centre for Infectious Diseases and Microbiology Laboratory Services	Centre for Infectious Diseases and Microbiology Laboratory Services	"John-Sebastian Eden, Jen Kok, Dominic Dwyer, Edward Holmes, Philip Britton, Alison Kesson, Elena Cutmore, Rachel Tulloch, Bethany Horsburgh"
EPI_ISL_2839351, EPI_ISL_2839354, EPI_ISL_2839356	Departments of Clinical Microbiology and Infectious Diseases	Centre for Infectious Diseases and Microbiology Laboratory Services	"John-Sebastian Eden, Jen Kok, Dominic Dwyer, Edward Holmes, Philip Britton, Alison Kesson, Elena Cutmore, Rachel Tulloch, Bethany Horsburgh"
EPI_ISL_2839358, EPI_ISL_2839360, EPI_ISL_2839362	Centre for Infectious Diseases and Microbiology Laboratory Services	Centre for Infectious Diseases and Microbiology Laboratory Services	"John-Sebastian Eden, Jen Kok, Dominic Dwyer, Edward Holmes, Philip Britton, Alison Kesson, Elena Cutmore, Rachel Tulloch, Bethany Horsburgh"
EPI_ISL_2839364	PathWest Laboratory Medicine WA Microbial Surveillance Unit	PathWest Laboratory Medicine WA Microbial Surveillance Unit	"Chisha Sikazwe, Avram Levy, David Smith, Chris Blyth, Alice Michie, Cara Minney-Smith, David Speers"
EPI_ISL_2839366, EPI_ISL_2839368	Centre for Infectious Diseases and Microbiology Laboratory Services	Centre for Infectious Diseases and Microbiology Laboratory Services	"John-Sebastian Eden, Jen Kok, Dominic Dwyer, Edward Holmes, Philip Britton, Alison Kesson, Elena Cutmore, Rachel Tulloch, Bethany Horsburgh"
EPI_ISL_2839372	PathWest Laboratory Medicine WA Microbial Surveillance Unit	PathWest Laboratory Medicine WA Microbial Surveillance Unit	"Chisha Sikazwe, Avram Levy, David Smith, Chris Blyth, Alice Michie, Cara Minney-Smith, David Speers"
EPI_ISL_2839374	Centre for Infectious Diseases and Microbiology Laboratory Services	Centre for Infectious Diseases and Microbiology Laboratory Services	"John-Sebastian Eden, Jen Kok, Dominic Dwyer, Edward Holmes, Philip Britton, Alison Kesson, Elena Cutmore, Rachel Tulloch, Bethany Horsburgh"
EPI_ISL_2839376, EPI_ISL_2839397, EPI_ISL_2839398, EPI_ISL_2839399, EPI_ISL_2839400, EPI_ISL_2839401, EPI_ISL_2839402, EPI_ISL_2839403, EPI_ISL_2839404, EPI_ISL_2839405, EPI_ISL_2839406, EPI_ISL_2839407, EPI_ISL_2839408, EPI_ISL_2839409, EPI_ISL_2839410, EPI_ISL_2839411, EPI_ISL_2839412, EPI_ISL_2839413, EPI_ISL_2839414, EPI_ISL_2839415			

see above	PathWest Laboratory Medicine WA Microbial Surveillance Unit	PathWest Laboratory Medicine WA Microbial Surveillance Unit	"Chisha Sikazwe, Avram Levy, David Smith, Chris Blyth, Alice Michie, Cara Minney-Smith, David Speers"
EPI_ISL_2839416, EPI_ISL_2839417	Centre for Infectious Diseases and Microbiology Laboratory Services	Centre for Infectious Diseases and Microbiology Laboratory Services	"John-Sebastian Eden, Jen Kok, Dominic Dwyer, Edward Holmes, Philip Britton, Alison Kesson, Elena Cutmore, Rachel Tulloch, Bethany Horsburgh"
EPI_ISL_2839418, EPI_ISL_2839438, EPI_ISL_2839439, EPI_ISL_2839440, EPI_ISL_2839441	PathWest Laboratory Medicine WA Microbial Surveillance Unit	PathWest Laboratory Medicine WA Microbial Surveillance Unit	"Chisha Sikazwe, Avram Levy, David Smith, Chris Blyth, Alice Michie, Cara Minney-Smith, David Speers"
EPI_ISL_2839442	Centre for Infectious Diseases and Microbiology Laboratory Services	Centre for Infectious Diseases and Microbiology Laboratory Services	"John-Sebastian Eden, Jen Kok, Dominic Dwyer, Edward Holmes, Philip Britton, Alison Kesson, Elena Cutmore, Rachel Tulloch, Bethany Horsburgh"
EPI_ISL_2839443, EPI_ISL_2839444, EPI_ISL_2839445, EPI_ISL_2839446, EPI_ISL_2839447, EPI_ISL_2839448	PathWest Laboratory Medicine WA Microbial Surveillance Unit	PathWest Laboratory Medicine WA Microbial Surveillance Unit	"Chisha Sikazwe, Avram Levy, David Smith, Chris Blyth, Alice Michie, Cara Minney-Smith, David Speers"
EPI_ISL_2839449	Centre for Infectious Diseases and Microbiology Laboratory Services	Centre for Infectious Diseases and Microbiology Laboratory Services	"John-Sebastian Eden, Jen Kok, Dominic Dwyer, Edward Holmes, Philip Britton, Alison Kesson, Elena Cutmore, Rachel Tulloch, Bethany Horsburgh"
EPI_ISL_2839450	Departments of Clinical Microbiology and Infectious Diseases	Centre for Infectious Diseases and Microbiology Laboratory Services	"John-Sebastian Eden, Jen Kok, Dominic Dwyer, Edward Holmes, Philip Britton, Alison Kesson, Elena Cutmore, Rachel Tulloch, Bethany Horsburgh"
EPI_ISL_2839452	Centre for Infectious Diseases and Microbiology Laboratory Services	Centre for Infectious Diseases and Microbiology Laboratory Services	"John-Sebastian Eden, Jen Kok, Dominic Dwyer, Edward Holmes, Philip Britton, Alison Kesson, Elena Cutmore, Rachel Tulloch, Bethany Horsburgh"
EPI_ISL_2839453	PathWest Laboratory Medicine WA Microbial Surveillance Unit	PathWest Laboratory Medicine WA Microbial Surveillance Unit	"Chisha Sikazwe, Avram Levy, David Smith, Chris Blyth, Alice Michie, Cara Minney-Smith, David Speers"
EPI_ISL_2839454	Centre for Infectious Diseases and Microbiology Laboratory Services	Centre for Infectious Diseases and Microbiology Laboratory Services	"John-Sebastian Eden, Jen Kok, Dominic Dwyer, Edward Holmes, Philip Britton, Alison Kesson, Elena Cutmore, Rachel Tulloch, Bethany Horsburgh"
EPI_ISL_2839455, EPI_ISL_2839456	Departments of Clinical Microbiology and Infectious Diseases	Centre for Infectious Diseases and Microbiology Laboratory Services	"John-Sebastian Eden, Jen Kok, Dominic Dwyer, Edward Holmes, Philip Britton, Alison Kesson, Elena Cutmore, Rachel Tulloch, Bethany Horsburgh"
EPI_ISL_2839457	Centre for Infectious Diseases and Microbiology Laboratory Services	Centre for Infectious Diseases and Microbiology Laboratory Services	"John-Sebastian Eden, Jen Kok, Dominic Dwyer, Edward Holmes, Philip Britton, Alison Kesson, Elena Cutmore, Rachel Tulloch, Bethany Horsburgh"
EPI_ISL_2989613	Monash Medical Centre	WHO Collaborating Centre for Reference and Research on Influenza	Xiaomin Dong, Michelle Francis, Tony Korman, Yi-Mo Deng, Ammar Aziz, Naomi Komadina
EPI_ISL_2989828	Royal Children's Hospital	WHO Collaborating Centre for Reference and Research on Influenza	Xiaomin Dong, Annette Alafaci,Yi-Mo Deng, Ammar Aziz, Naomi Komadina
EPI_ISL_2991498	PathWest Laboratory Medicine WA Microbial Surveillance Unit	PathWest Laboratory Medicine WA Microbial Surveillance Unit	Chisha Sikazwe, Avram Levy, David Smith, Chris Blyth, Alice Michie, Cara Minney-Smith, David Speers
EPI_ISL_412458	Centro de Salud Sócrates Flores Vivas	University of Edinburgh	Gordon, A., Alanis,R., Balmseda,A., Schiller,A., Patel,M., Kubale,J., Tan,G., Pickett,B., Fedorova,N., Amedeo,P., Hu,L., Christensen,J., Miller,J., Durbin,A., Williams,T., Arumemi,F., Cadiz,C., Williams, T.
EPI_ISL_412460, EPI_ISL_412461	Virology Laboratory, Ricardo Gutiérrez Children's Hospital	Virology Laboratory, Ricardo Gutiérrez Children's Hospital / Vanderbilt University Medical Center	Goya, Stephanie; Lucion, Maria Florencia; Juarez, Maria del Valle; Shilts, Meghan; Gentile, Angela; Mistchenko, Alicia S.; Das, Suman & Viegas, Mariana
EPI_ISL_412643	Virology Laboratory Ricardo Gutiérrez Children's Hospital	Virology Laboratory Ricardo Gutiérrez Children's Hospital	Goya, Stephanie.; Nabaes Jodar, Mercedes S.; Valinotto, Laura E.; Rojo, Gabriel L.; Zaiat, Jonathan; Marti, Marcelo A.; Mistchenko, Alicia S.; Viegas, M.
EPI_ISL_412857, EPI_ISL_412858	Virology Laboratory, Ricardo Gutiérrez Children's Hospital	Virology Laboratory, Ricardo Gutiérrez Children's Hospital / Vanderbilt University Medical Center	Goya, Stephanie; Lucion, Maria Florencia; Juarez, Maria del Valle; Shilts, Meghan; Gentile, Angela; Mistchenko, Alicia S.; Das, Suman & Viegas, Mariana
EPI_ISL_412859	WHO National Influenza Centre Russian Federation	WHO National Influenza Centre Russian Federation	Komissarova K.,Fadeev A., Komissarov A., Krivitskaya V.
EPI_ISL_412863	Virology Laboratory, Ricardo Gutiérrez Children's Hospital	Virology Laboratory, Ricardo Gutiérrez Children's Hospital / Vanderbilt University Medical Center	Goya, Stephanie; Lucion, Maria Florencia; Juarez, Maria del Valle; Shilts, Meghan; Gentile, Angela; Mistchenko, Alicia S.; Das, Suman & Viegas, Mariana
EPI_ISL_412864	Virology Laboratory, Ricardo Gutiérrez Children's Hospital.	Virology Laboratory, Ricardo Gutiérrez Children's Hospital / Vanderbilt University Medical Center.	Goya, Stephanie; Lucion, Maria Florencia; Juarez, Maria del Valle; Shilts, Meghan; Gentile, Angela; Mistchenko, Alicia S.; Das, Suman & Viegas, Mariana
EPI_ISL_412865	Respiratory Virus Unit, Microbiology Services Colindale, Public Health England	Microbiology Services Colindale, Public Health England	Zambon M
EPI_ISL_412866	Respiratory Virus Unit, Microbiology Services Colindale, Public Health England	Microbiology Services Colindale, Public Health England	Zambon M.
EPI_ISL_412867, EPI_ISL_412868	Respiratory Virus Unit, Microbiology Services Colindale, Public Health England	Microbiology Services Colindale, Public Health England	Zambon M
EPI_ISL_413222, EPI_ISL_413293, EPI_ISL_413352, EPI_ISL_413353	Institut Pasteur de Madagascar	Institut Pasteur de Madagascar	Jean-Michel HERAUD
EPI_ISL_4569432, EPI_ISL_4602779	VIC, Victorian Infectious Diseases Reference Laboratory	WHO Collaborating Centre for Reference and Research on Influenza	Xiaomin Dong, Annette Alafaci,Yi-Mo Deng, Ammar Aziz, Naomi Komadina
EPI_ISL_732337, EPI_ISL_732338, EPI_ISL_732340, EPI_ISL_732341, EPI_ISL_732342, EPI_ISL_732343, EPI_ISL_732344, EPI_ISL_732345, EPI_ISL_732346, EPI_ISL_732347, EPI_ISL_732348, EPI_ISL_732349, EPI_ISL_732350, EPI_ISL_732351, EPI_ISL_732352, EPI_ISL_732353, EPI_ISL_732354, EPI_ISL_732355, EPI_ISL_732356, EPI_ISL_732358, EPI_ISL_732359, EPI_ISL_732360, EPI_ISL_732361, EPI_ISL_732362, EPI_ISL_732363, EPI_ISL_732364, EPI_ISL_732365, EPI_ISL_732366, EPI_ISL_732367, EPI_ISL_732368, EPI_ISL_732369, EPI_ISL_732371, EPI_ISL_732372, EPI_ISL_732373			
see above	Respiratory Virus Unit, National Infection Service, Public Health England	National Infection Service, Public Health England	Zambon M, Talts T, Ellis J, Miah S, Platt S
