# Supplementary material for: Off-season RSV epidemics in Australia after easing of COVID-19 restrictions
Source: Nat Commun. 2022 May 24;13:2884. doi: 10.1038/s41467-022-30485-3 (PMC9130497; doi:10.1038/s41467-022-30485-3)
Supplement: Supplementary file 7 — Reporting Summary [file 41467_2022_30485_MOESM7_ESM.pdf]

nature portfolio

Corresponding author(s): John-Sebastian Eden

Last updated by author(s): Mar 15, 2022

Reporting Summary

Nature Portfolio wishes to improve the reproducibility of the work that we publish. This form provides structure for consistency and transparency in reporting. For further information on Nature Portfolio policies, see our Editorial Policies and the Editorial Policy Checklist.

Statistics

For all statistical analyses, confirm that the following items are present in the figure legend, table legend, main text, or Methods section.

n/a	Confirmed	
☐	☒	The exact sample size (*n*) for each experimental group/condition, given as a discrete number and unit of measurement
☒	☐	A statement on whether measurements were taken from distinct samples or whether the same sample was measured repeatedly
☒	☐	The statistical test(s) used AND whether they are one- or two-sided<br>*Only common tests should be described solely by name; describe more complex techniques in the Methods section.*
☐	☒	A description of all covariates tested
☒	☐	A description of any assumptions or corrections, such as tests of normality and adjustment for multiple comparisons
☒	☐	A full description of the statistical parameters including central tendency (e.g. means) or other basic estimates (e.g. regression coefficient) AND variation (e.g. standard deviation) or associated estimates of uncertainty (e.g. confidence intervals)
☐	☒	For null hypothesis testing, the test statistic (e.g. *F*, *t*, *r*) with confidence intervals, effect sizes, degrees of freedom and *P* value noted<br>*Give P values as exact values whenever suitable.*
☒	☐	For Bayesian analysis, information on the choice of priors and Markov chain Monte Carlo settings
☒	☐	For hierarchical and complex designs, identification of the appropriate level for tests and full reporting of outcomes
☒	☐	Estimates of effect sizes (e.g. Cohen's *d*, Pearson's *r*), indicating how they were calculated

Our web collection on statistics for biologists contains articles on many of the points above.

Software and code

Policy information about availability of computer code

Data collection	No software was used for data collection.
Data analysis	All tools used are open-source unless otherwise stated. Sequence trimming was performed with BBDuk v37.98 before genome assembly with MEGAHIT v1.1.3 or IRMA v0.93. The full assembly pipeline can be found at https://github.com/jsede/virus_assembly/. The final assemblies were visually confirmed in the commercial software Geneious Prime v.2020.0.3. Sequence alignments were performed with MAFFT v.7. Temporal signal was explored using TempEst v.1.5. Phylogenetic analyses were performed using FastTree v.2.1, IQ-TREE v.2.0, RAxML v.8, BEAST.v.1.10. Estimates of selection strength were performed using HYPHY tool - SLAC, SNAP v.2.1.1, Contrast-FEL and aBSREL. Code use for analysis can be found at https://github.com/jsede/RSV_2020.

For manuscripts utilizing custom algorithms or software that are central to the research but not yet described in published literature, software must be made available to editors and reviewers. We strongly encourage code deposition in a community repository (e.g. GitHub). See the Nature Portfolio guidelines for submitting code & software for further information.

Data

Policy information about availability of data

All manuscripts must include a data availability statement. This statement should provide the following information, where applicable:

- Accession codes, unique identifiers, or web links for publicly available datasets
- A description of any restrictions on data availability
- For clinical datasets or third party data, please ensure that the statement adheres to our policy

The sequence data generated in this study have been deposited in the NCBI GenBank database under accession codes OM857140 - OM857397, and the GISAID

EpiRSV database with accession numbers EPI_ISL_1653938 to EPI_ISL_1653948, EPI_ISL_2543762 to EPI_ISL_2543853, and EPI_ISL_2839170 to EPI_ISL_2839457 (see Supplementary Table 1 and Supplementary Data 1). Reference sequences were downloaded from the NCBI GenBank and GISAID EpiRSV databases.

Field-specific reporting

Please select the one below that is the best fit for your research. If you are not sure, read the appropriate sections before making your selection.

☒ Life sciences ☐ Behavioural & social sciences ☐ Ecological, evolutionary & environmental sciences

For a reference copy of the document with all sections, see nature.com/documents/nr-reporting-summary-flat.pdf

Life sciences study design

All studies must disclose on these points even when the disclosure is negative.

Sample size	In this study, no sample size calculations were performed; however the RSV positive samples were collected from major diagnostic labs offering statewide testing to ensure sampling was geographically well-represented. Furthermore, >100 genomes were available for each period/site to ensure sufficient sampling. Samples were also intentionally selected to provide consistent temporal signal across the pre- and post-COVID-19 period.
Data exclusions	Low quality sequences were removed including those with excessive ambiguous nucleotides (>5% of genome/gene) or unexpected divergence relative to collection date based on alignment temporal signal. For the G gene analysis (Fig 3), sequences less than 300 nt were also excluded to based on pre-established criteria to ensure phylogenetic signal.
Replication	Phylogenetic analyses were conducted by two groups (Eden and Dhanasekaran) using either phylogenetic bootstrapping (1000 replicates) or multiple independent mcmc chains (at least 2) to ensure statistical convergence. Outputs were compared between analyses and groups to ensure successful replication and statistical convergence.
Randomization	Randomization was not required for our study as it contained no experimental groups or comparisons.
Blinding	This study used viral genome sequences from de-identified, residual diagnostic specimens therefore blinding was not relevant.

Reporting for specific materials, systems and methods

We require information from authors about some types of materials, experimental systems and methods used in many studies. Here, indicate whether each material, system or method listed is relevant to your study. If you are not sure if a list item applies to your research, read the appropriate section before selecting a response.

Materials & experimental systems

n/a	Involved in the study	
☒	☐	Antibodies
☒	☐	Eukaryotic cell lines
☒	☐	Palaeontology and archaeology
☒	☐	Animals and other organisms
☐	☒	Human research participants
☒	☐	Clinical data
☒	☐	Dual use research of concern

Methods

n/a	Involved in the study	
☒	☐	ChIP-seq
☒	☐	Flow cytometry
☒	☐	MRI-based neuroimaging

Human research participants

Policy information about studies involving human research participants

Population characteristics	Samples were collected from RSV-infected patients presenting for diagnostic testing without specific restrictions of age. All patient data was de-identified with only basic demographic collected (age, gender, postcode). Here, samples were mostly collected from young children (median ages between 0.78 - 2.34 years for the different states), although all age groups including adults and the elderly were represented (age range 0.03 to 91.72 years). Across the cohort the ratio of male to female patients was 0.97:1.
Recruitment	Samples were sequenced from cases collected for routine diagnostic purposes as part of public health responses.
Ethics oversight	The study was approved by the Western Sydney Local Health District Human Research Ethics Committee (WSHLD-HREC) with approval number LNR/17/WMEAD/128, and The Royal Children's Hospital Human Research Ethics Committee with approval number 37185.

Note that full information on the approval of the study protocol must also be provided in the manuscript.
